# Exploring apathy components and their relationship in cognitive decline: insights from a network cross-sectional study

**DOI:** 10.1186/s40359-024-02239-x

**Published:** 2025-02-17

**Authors:** Pierfrancesco Sarti, Simone Varrasi, Claudia Savia Guerrera, Giuseppe Alessio Platania, Giovanna Furneri, Vittoria Torre, Francesco Maria Boccaccio, Veronica Rivi, Sophie Tascedda, Concetta Pirrone, Mario Santagati, Johanna M. C. Blom, Sabrina Castellano, Filippo Caraci

**Affiliations:** 1 Department of Biomedical, Metabolic and Neural Sciences, University of Modena and Reggio Emilia, Via Giuseppe Campi 287, 41125 Modena, Italy; 2 Department of Educational Sciences, University of Catania, Via Biblioteca, 95124 Catania, Italy; 3 Department of Biomedical and Biotechnological Sciences, University of Catania, Via Santa Sofia 97, 95123 Catania, Italy; 4 Faculté de Biologie et de Médecine, Université de Lausanne, Lausanne, Switzerland; 5 ASP3 Catania, Department of Mental Health, Alzheimer Psychogeriatric Center, Corso Italia 234, 95127 Catania, Italy; 6 Center for Neuroscience and Neurotechnology, University of Modena and Reggio Emilia, Via Giuseppe Campi, 41125 Modena, Italy; 7 Department of Drug and Health Sciences, University of Catania, Viale Andrea Doria 6, 95124 Catania, Italy; 8 Oasi Research Institute-IRCCS, Via Conte Ruggero, 94018 Troina, Italy; 9 Plateforme de Bioinformatique, Centre Hospitalier Universitaire Vaudois (CHUV), Lausanne, Switzerland Service de Chimie Clinique CHUV, Lausanne, Switzerland

**Keywords:** Network analysis, Dementia, Alzheimer’s disease, MCI, Cognition, Intervention strategies, Cognitive apathy

## Abstract

**Background:**

Apathy worsens with age and cognitive decline, particularly in Alzheimer’s, leading to functional and cognitive deterioration. Comprehending its broad impact is vital for customized, preventive treatments.

**Methods:**

The study examined 214 adults divided in three groups—Mild Cognitive Impairment, mild Alzheimer’s, and controls—using neuropsychological tests and questionnaires, with statistical and network analysis to explore apathy’s links with other group variables related to demographics and treatment.

**Results:**

Notable differences were observed among the groups' performance of administered tests. While inferential statistics failed to return a predictive model of apathy in mild Alzheimer’s, networks and cluster analyses indicate that the demographic variables analysed have different importance at different times of disease progression and that cognitive apathy is particularly prominent in AD-related decline.

**Conclusions:**

Network analysis revealed insights into dementia risk differentiation, notably the impact of sex and demographic factors, beyond the scope of traditional statistics. It highlighted cognitive apathy as a key area for personalized intervention strategies more than behavioural and emotional, emphasizing the importance of short-term goals and not taking away the person's autonomy when not strictly necessary.

**Supplementary Information:**

The online version contains supplementary material available at 10.1186/s40359-024-02239-x.

## Background

Apathy is defined as a quantifiable reduction in goal-directed activity characterized by at least two of the following impaired dimensions: behaviour/cognition, emotion, and social interaction [1]. Furthermore, deficits should persist for four weeks and not be due to other factors [2–4]. Also, apathy intensifies with advancing age in individuals with normal and compromised cognitive abilities [5] and is prevalent in depressive states [6]. A lower educational background is associated with elevated apathy levels [7], although its prevalence across sexes remains to be defined [6]. In cognitive impairment, apathy is highly prevalent, persistent, burdening, and strongly linked to mortality [8].

Apathy stands out as a principal non-cognitive manifestation of Alzheimer’s Disease (AD) [9, 10] and is correlated with the Aβ pathology [11–13]. It also contributes to decline independently of depression [14]. Apathy alone explains 27% of the variance in ‘instrumental abilities’, and predicts faster cognitive and functional deterioration [15, 16].

Recent studies and comprehensive reviews indicate that apathy may contribute to the risk of cognitive decline, even among the general population, with a significant link to impairments in executive functions [17]. Higher levels of apathy in Mild Cognitive Impairment (MCI) double the likelihood of developing AD, independent of depression [14, 18, 19]. Moreover, longitudinal studies demonstrate that persistent apathy is associated with a rapid one-year functional decline in patients with either AD, dementia with Lewy bodies, Huntington’s Disease, or Parkinson’s Disease [20–23]. In this context, several researchers attempted to pinpoint specific aspects of apathy, such as diminished interest, reduced initiative, and emotional dullness, as components contributing to the progression of cognitive impairment [24].

Indeed, patients presenting a lack of interest show a higher conversion rate to AD, even after controlling for age, sex, and education [25]. A decrease in intellectual curiosity, defined as a reduction in daily productivity and lack of initiative, is also associated with poorer cognitive performance [26]. These observations align well with new ways of understanding apathy, viewing it as a misperception of available energy for behavioural, cognitive, and emotional tasks [27]. According to this perspective, effort-based decision making for reward is crucial to analyse motivated behaviour and its disturbances, promoting a neurobiological-driven transdiagnostic approach for understanding, assessing, and treating apathy with specific tools [28]. This may also explain the strong connection between apathetic symptoms and neuropathophysiological pathways.

However, experts differ in their views on how various aspects of apathy predict cognitive health conditions. This uncertainty could result from limitations in the research, such as small participant groups, the absence of follow-up studies, or the lack of comparisons between subjects with impairments and those without [29]. Furthermore, the unclear categorization of apathy complicates understanding which aspect most significantly impacts cognitive decline [30]. These shortcomings hinder the development of targeted strategies for prevention, early diagnosis, and pharmacological treatment of apathetic symptomatology.

Hence, it is crucial to examine the interactions between key variables across various stages of cognitive health to pinpoint the most critical factors for observation, evaluation, and intervention to foster healthy aging [31]. Employing Network Analysis, a technique that maps the interplay of symptoms to determine their significant effects on health outcomes, can facilitate this process [32–34]. For instance, van Wanrooij et al. utilized this methodology to investigate the interrelations among apathy, depression, dementia, and functional disability. Their research found significant links between apathy, mood disturbances, and functional challenges, with future dementia being linked to apathy, mediated by depression [35].

Our study aims to extend previous knowledge by comparing the network structures of individuals with healthy cognition, MCI, and various stages of AD. Network Analyses were conducted on corresponding samples of older adults (healthy control, MCI, and AD), incorporating demographic (age, sex, education) and clinical (general cognitive performance, executive functioning, depressive symptoms, apathy, comorbidity, pharmacological treatment, rehabilitation) variables. Specifically, apathy was explored by considering the items composing its psychometric measure, to identify possible critical roles played by single dimensions.

## Methods

### Participants

The sample included 214 adults divided into three groups: an amnesic MCI group (size = 77, 54 Females and 23 Males, mean age of 75.53 ± 7.3), a mild AD group (size = 30 patients, 19 females and 11 males, mean age of 76.33 ± 6.4), and a Control group (size = 107, 78 Females and 29 Males, with a mean age of 74.06 ± 6.8).

The MCI and mild AD groups were enrolled at a specialized public service for cognitive impairment, called “U.O.S. Centro Alzheimer e Psicogeriatria”, ASP3 Catania, Italy.

Cognitive diagnoses were clinical and not biological. Specific inclusion and exclusion criteria were used following the protocols of the National Institute of Aging (NIA) and the Alzheimer’s Association Work Group for amnesic MCI and AD [36]. In more detail, criteria [37] for clinical diagnosis of amnesic MCI were taken into account as follows:

- Cognitive concern reflecting a change in cognition reported by patient or informant or clinician.
- Objective evidence of impairment in one or more cognitive domains, typically including memory.
- Preservation of independence in functional abilities.
- Absence of dementia.

Criteria for clinical diagnosis of probable AD, instead, were the following [38]:

- Cognitive or behavioural symptoms that interfere with the ability to function at usual activities, represent a decline from previous levels of functioning and performing, and are not explained by delirium or major psychiatric disorders.
- Cognitive impairment detected through a combination of history-taking from the patient and a knowledgeable informant, and an objective cognitive assessment.
- Cognitive or behavioural impairment involving a minimum of two among impaired ability to acquire and remember new information, impaired reasoning and handling of complex tasks, impaired visuospatial abilities, impaired language functions, and changes in personality.
- Insidious onset.
- Clear-cut history of worsening of cognition by report or observation.
- Initial and most prominent cognitive deficits showing either an amnestic presentation (the most common), or non-amnestic presentation.
- Absence of cerebrovascular disease, dementia with Lewy bodies, prominent features of behavioural variant frontotemporal dementia, prominent features of semantic variant primary progressive aphasia or non-fluent/agrammatic variant primary progressive aphasia, and absence of other concurrent neurological, non-neurological, or medications that could interfere.

Patients were tested on their scheduled appointments at the service with the Italian standardized version of the Mini-Mental State Examination (MMSE) [39, 40]], which, according to the Italian AIFA (Agenzia Italiana del Farmaco) guidelines [41], is the recommended test for cognitive deterioration staging. Indeed, this test was specifically used only to screen prospective participants and preliminarily divide them into Control, MCI, and mild AD groups. Such division was then confirmed by considering both clinical history and performance at other neuropsychological tests, consistently with the guidelines cited previously.

Older individuals with a total MMSE age- and education-adjusted score ≥ 18 and < 28 were included, while patients with a recent history of cerebral ischemia and psychotic episodes were excluded. As a result, 107 amnesic MCI and mild AD subjects were enrolled in the study. The Control group, instead, was made up of 107 healthy volunteers with a MMSE score ≥ 28. Coherently with the cited guidelines, with DSM-5-TR [42] and with ICD-11 [43], their clinical history, cognitive performance, and daily functioning were considered for confirming their healthy status and excluding mild neurocognitive disorder. Information on their usual autonomy was collected from both the participant and a knowledgeable person. Moreover, MoCA total score above 26 was considered as an additional warranty for the absence of a concurrent pre-clinical cognitive decline, as a total score of 26 was more conservative than all the other cut-offs proposed for Italian population for discriminating healthy subjects from MCI [44, 45].

All participants signed an informed consent and were tested individually in a single session by expert clinical psychologists trained in dementia. After screening, more women than men were enrolled in the study, which reflects the Italian and global sex prevalence of cognitive impairment [46].

### Procedures and measures

Tests and questionnaires were administered to patients with MCI, and mild Alzheimer’s Disease and the Control group by trained psychologists.

The Mini-Mental State Examination (MMSE) [39, 40] is a widely used screening tool to assess cognitive functioning and detect cognitive impairment. The MMSE consists of a series of questions and tasks that measure different cognitive domains, including orientation, information recording and recall, attention and calculation, language skills, and visuo-constructive skills. The maximum possible score on the MMSE is 30 points, with higher age- and education-adjusted scores indicating better cognitive function.

The Montreal Cognitive Assessment (MoCA) [47, 48] is a rapid screening tool for assessing overall cognitive functioning, and specifically for MCI. It measures a range of cognitive domains, such as attention, concentration, executive functions, memory, language, visuospatial skills, abstraction, calculation, and orientation. The scoring system assigns a maximum of 30 points, with an age- and education-adjusted score of 26 or above considered within the normal range. However, in our study we took into account the specific cut-off scores of southern Italian population, which are lower than 26, as our participants belonged to that specific geographical area. In more detail, optimal cut-off for a diagnosis of probable AD was a MoCA score ≤ 14, while optimal cut-off for probable cognitive impairment was a MoCA score ≤ 17 [44].

Frontal Assessment Battery (FAB) [49] is a hetero-administered test useful for assessing specific frontal functions such as conceptualization (analogies), lexical fluency, motor series, interference sensitivity, inhibitory control, and environmental dependence. Scores from 0 (task failure) to 3 (no errors) are given. Once the scores are summed, an adjustment is made for age and schooling.

Hamilton Depression Rating Scale (HDRS) [50] is a 21-item hetero-administered scale in which different affective components are explored in assessing the subject’s depressive state. A score < 7 indicates no depression; between 8 and 17 indicates mild depression; between 18 and 24 moderate depression; > 24 severe depression.

The Apathy Evaluation Scale (AES-C) [2, 51] is a 4-point Likert scale featuring 18 items, requiring approximately 10–20 min for administration. The Clinician Version designates item categorization in the right column as B (behavioural item), C (cognitive item), or E (emotional item). Items are phrased with positive or negative syntax (+ or −), with most leaning towards the positive side. The Self-evaluation (SE) and quantifiable (Q) items are identified in the right column of the AES-C. Scores on the AES-C span from 18 to 72, and the cut-off score varies from 39 to 41, depending on the AES version used. The clinical correlation suggests that these cut-offs may be slightly conservative. In this study, the AES-C was compiled by a clinician after a semi-structured interview with the patient to ensure accurate symptom reporting, especially in cases of depression or apathy.

The English version and the recent Italian version (used here) [51] are provided as Supplementary Material.

In addition to these, other variables were also included in the network models for all three groups. Those in common to all three groups were the sex of the participants/patients, age, and level of schooling. Only for the MCI and mild AD groups, also the presence of comorbidities, whether they were undergoing pharmacological treatment, and whether they were doing neurocognitive rehabilitation was considered. Comorbidities were related to depressive and anxious symptomatology only in six MCI patients, who were treated with second-generation antidepressants for more than three months (vortioxetine 5–10 mg/die or escitalopram 5–10 mg/die). Only 26 MCI patients out of 77 were treated with low dosages of cholinesterase inhibitors (donepezil 2.5–5 mg/die or rivastigmine patch 4.6 mg/die) administered for more than three months. The cohort of 30 mild AD patients was recruited in the absence of or just before the starting of any treatment with cholinesterase inhibitors. Rehabilitation was based on weekly sessions of cognitive stimulation. Sex, comorbidities, pharmacological treatment, and rehabilitation were treated as dichotomous categorical variables.

The network models discussed below serve two main purposes. First, they begin with what are termed ‘generic’ models, which use the Apathy Evaluation Scale based on a total score — this score is the sum of individual item scores. This approach enables the integration of findings from conventional statistical methods with network analysis insights, particularly regarding how demographic and neurocognitive factors relate to apathy. Second, more detailed models were developed, breaking down the Apathy Evaluation Scale into individual items, along with the other previously mentioned variables. This allows for a more nuanced understanding of how specific aspects of apathy — cognitive, emotional, and behavioural — differentially impact cognitive decline. By creating a more complex network model that maps these detailed interactions, it is possible to highlight the distinct roles these components play at different stages of disease progression, even if the cross-sectional design of our study did not allow to draw any inference on their specific role over time.

### Statistical analysis

The data were analysed quantitatively to extrapolate descriptive information from the sample using mean, standard deviation, and percentages. The raw scores of the three neuropsychological tests (MoCA, MMSE, and FAB), unadjusted for age and schooling, and the overall and individual item scores of the AES were compared between the control group and the other two groups with independent samples t-tests. The decision to use test scores unadjusted for age and schooling stems from the fact that statistical and network analyses would already have included age, gender, and schooling as stand-alone variables. Ignoring this detail could have led to multicollinearity effects.

Secondly, analysis of variance (ANOVA) was also performed. The normal distribution of the variables was checked using the Shapiro–Wilk test (*p* > 0.05 for the assumption of normality). Homogeneity of variance was established using Levene’s test (*p* > 0.05 for the assumption of equal variances). The correlation analysis between the variables was carried out according to the results of the demographic and distribution analyses.

A regression analysis was conducted to identify in each group whether the overall apathy score could be predicted by the other variables considered. Individual items were not included in the model in order not to create multicollinearity effects given the nature of the factorial model already identified in the Apathy Evaluation Scale.

Then, we analysed whether the errors/residuals were normally distributed and independent to assess the option of applying robust regression methods. Finally, the best model was chosen using Akaike’s Criterion. The effects of the predictors are considered significant at a *p* < 0.05 level.

In the end, a Clustering Analysis Algorithm and a Principal Component Analysis were performed to evaluate if there were any characteristics or a combination of those that could express the differences between the three groups.

All statistical analyses were conducted using SPSS version 28.0 (SPSS Inc., Chicago, IL, United States) and RStudio software (Version 4.3.2, 2023–10-10).

### Network analysis

Two network models were estimated for each group, six in total. The structure of the networks represented by the models was compared both visually and by comparing the differences in the amount of variance explained for each variable and in the significance of the edges. The networks represent graphical mixed models (GMMs) consisting of a parameterized joint probability density given by the combination of continuous and categorical discrete variables [52]. The resulting network structure reveals direct associations between variables and allows questions to be asked about the selection, classification, and influence of and between nodes (variables).

All relations represented are pairwise interactions (k = 2). This means that the edges (links) are the estimated relationships between the variables taken two by two (partial correlations) controlling for all other variables in the network. The absence of an edge between two variables is a sign that they are conditionally independent given all the others.

The networks in their estimation were subjected to two different types of regularisations. For the control group, a LASSO regularisation was used as the sample was larger and it was decided to control for possible spurious connections [53].

For the group with Mild Cognitive Impairment and mild Alzheimer’s Disease, having a smaller number of subjects, k-fold cross-validation (10 folds) was used to limit Lq-penalisation in the estimation of false positives. This method divides the dataset into 10 random parts. Nine of these are used for learning and one-tenth for testing. The procedure is then repeated 10 times, each time using a different tenth for testing [54].

The “qgraph” package was used to represent the networks [55]. Two layouts were used: (i) the first in which the Apathy Evaluation Scale is represented with its total score, place all nodes in a circle (R code: layout = “circle”); (ii) the second, in which the AES was represented with each of its items, is determined by the Fruchterman and Reingold algorithm [56], which transforms the network into a system of particles with mass (R code: layout = “spring”).

A predictability representation (i.e., nodes predictability) was added to each node in the network. This measures how much variance of each node is explained by the variables with which it has connections [57]. High predictability values indicate that this variable is well explained by the variance of others. For continuous variables, R2 was used (i.e., the proportion of variance explained), and for dichotomous variables, accuracy/corrected classification (“CC”) was used as a form of predictability along with normalized accuracy (“nCC”).

Next, centrality measures were calculated for each network: the “Strength of centrality” (SC) which consists of the number and weight of connections of each node. If a variable has more connections or ‘thicker’ connections (with a very positive or very negative value), it means that it will influence and/or be influenced by many other variables or few but strongly. “Betweenness centrality” (BC) is the parameter that indicates how much a node is included in the ‘shortest paths’ between other nodes and indicates which nodes bridge, thus facilitating the connection between others in the network. “Expected Influence” (EI) seeks to evaluate the character and extent of a node's overall influence within the network, and consequently, the role it is anticipated to play in the network's activation, persistence, and resolution [58].

To interpret these measures and assess stability, bootstraps were performed for each network using the "bootnet" package by simulating a sample size of *n* = 500. The indices of the bootstrapped sample and its subgroups were then correlated with those of the actual sample of subjects. Correlation values above 0.5 are considered acceptable.

Finally, after transforming the networks into igraph objects [59], we ran a clustering algorithm to explore the presence of different structures in the connectivity between the three networks. Clusters of nodes represent groups of variables (sub-networks) that are more connected to each other and can therefore be affected more quickly when factors that change the network equilibrium intervene. We used the “walktrap” algorithm [60] that identifies clusters through random walks between connections in the network. Several estimations were performed with increasing steps and then the number that resulted in the first stable number of clusters was chosen.

## Results

### Descriptive and inferential analysis

The demographic analyses of the sample are reported in Table 1. The numerosity of the sample of the mild Alzheimer’s group respects the prevalence of this disease in the Italian population [61]. Specifically reported, is the finding that in the three groups, from the Controls to subjects diagnosed with mild AD, the average education decreases in a statistically significant manner (from 10.29 ± 5.8 to 6.87 ± 4.2 years of schooling, *p* = 0.004). Moreover, the MCI and mild AD groups displayed comorbidity with anxiety and/or depression-related symptoms, 24% and 30% respectively. More than half of both groups (58.4% MCI and 73.3% mild Alzheimer’s) were enrolled in neurocognitive rehabilitation and 33% of the subjects in the MCI group were treated pharmacologically.

**Table 1:** Table with demographic data of the three different groups recruited in the study

Demographics	Control Group		Mild Cognitive Impairment		Mild Alzheimer’s Disease	
	**Sample**	**Percentage**	**Sample**	**Percentage**	**Sample**	**Percentage**
Sample size	107	100	77	100	30	100
Females	78	72.9	54	70.1	19	63.3
Males	29	27.1	23	29.9	11	36.7
Mean Age	74.06 ± 6.8		75.53 ± 7.3		76.33 ± 6.4	
Years of Education						
- Mean Education	10.29 ± 5.7		8.56 ± 4.4		6.87 ± 4.2	
- Primary school	42	39.1	35	45.5	17	56.5
- Secondary school	14	13.1	18	23.4	7	23.4
- High School	22	20.5	15	19.5	4	13.3
- University and more	29	27.1	9	11.7	2	6.6
Comorbidity	0	0	19	24.7	9	30
Under Treatment	0	0	26	33.8	0	0
Doing Rehabilitation	0	0	45	58.4	22	73.3

Particularly in the MCI and mild AD groups, most of the variables were not normally distributed.

The non-parametric ANOVA and the Kruskal–Wallis post-hoc test conducted showed significant differences between all three groups for all the variables analysed. In particular, the three groups displayed statistical differences related to the cognitive tests (MMSE: Control vs. MCI, F = 4.39 *p* < 0.0001, MCI vs. mild Alzheimer, F = 2.94 *p* < 0.0001; MoCA: Control vs. MCI, F = 10.43 *p* < 0.0001, MCI vs. mild Alzheimer, F = 4.77 *p* < 0.0001; FAB: Control vs. MCI, F = 4.85 *p* < 0.0001). FAB mean scores were not significantly different between MCI and mild Alzheimer’s patients (F = 1.73 *p* = 0.059).

**Fig. 1:**
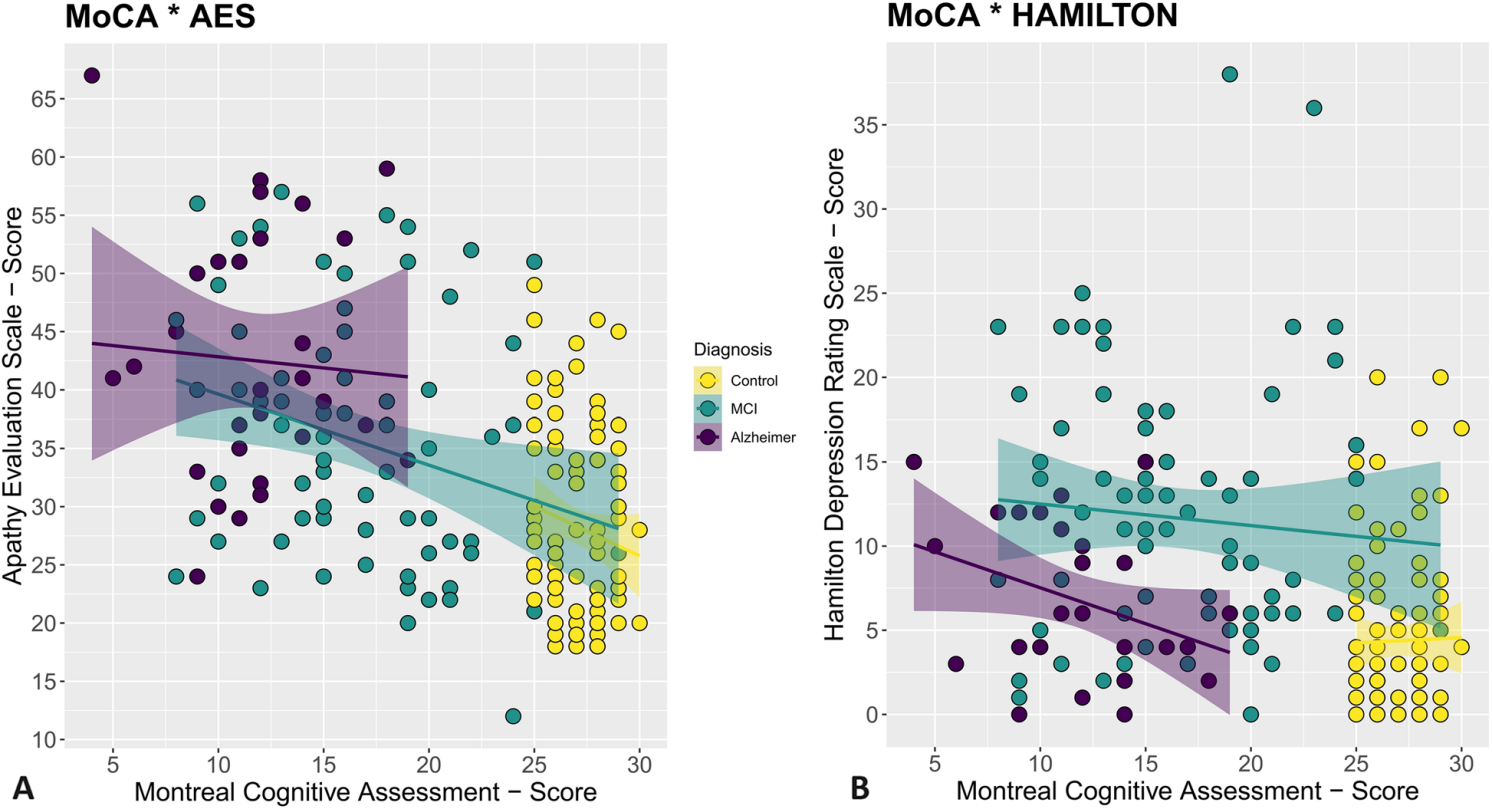
part A representation of the three groups showing the relationship between apathy (x-axis) and cognitive decline using the MoCA test (y-axis). The size of the dots indicates a higher or lower score on the Hamilton scale (severity of depressive symptoms). The trend line is also shown for each of the groups

The Hamilton Depression Rating Scale revealed significant score differences across the groups: the MCI group scored 7.28 points higher (*p* < 0.0001), and the mild Alzheimer’s group scored 2.39 points higher (*p* = 0.034) compared to the Control group. Finally, the Apathy Evaluation Scale score increased compared to the Control group by 7.31 points for MCI and 14.1 points for patients with mild Alzheimer’s Disease (both *p* < 0.0001). In Fig. 1 we report the chart representing, at the same time, the sample of controls and patients highlighting both the relationships between MoCA and the Apathy Evaluation Scale and between MoCA and the Hamilton Depression Rating Scale.

**Fig. 2:**
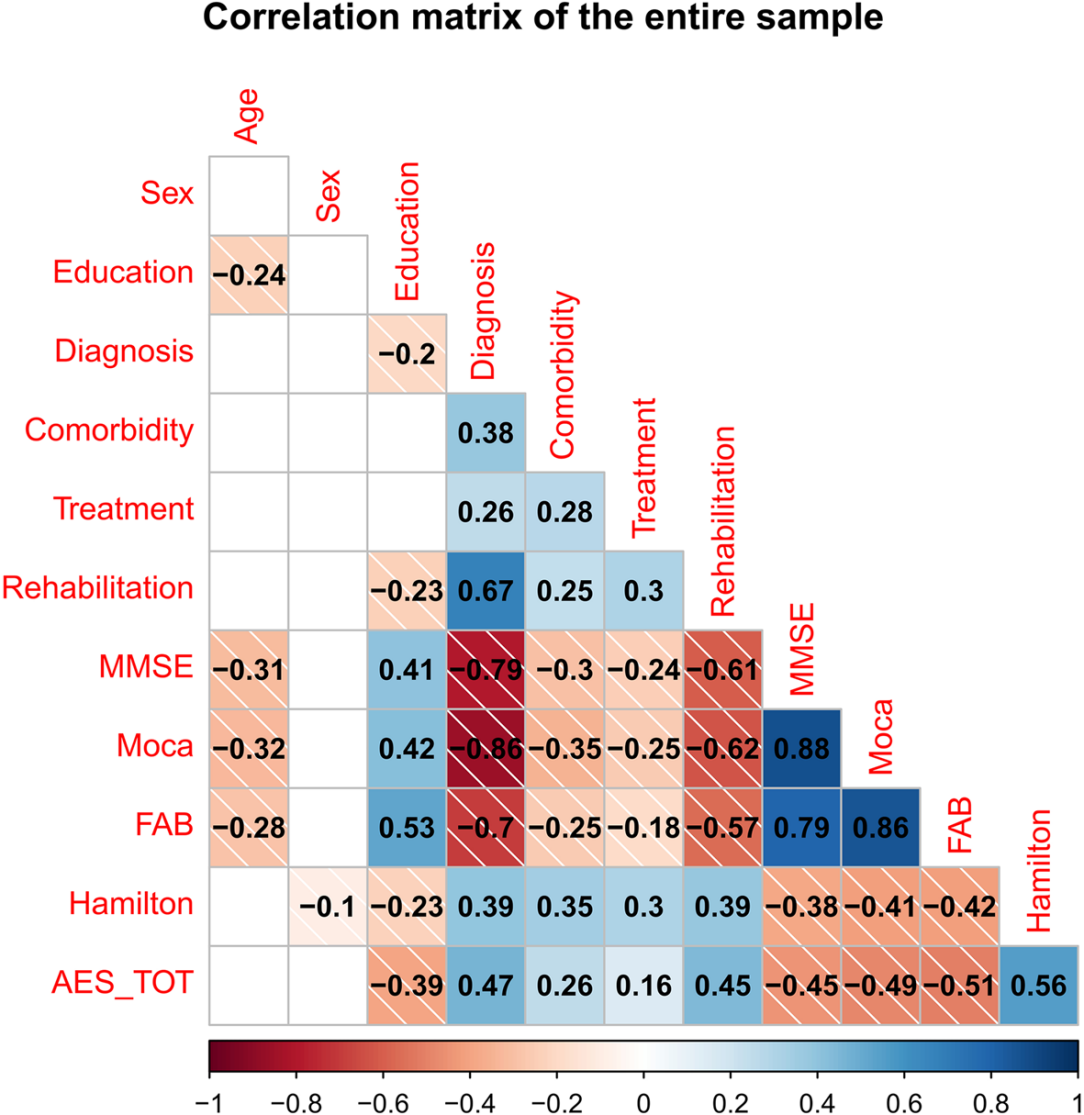
Spearman correlation tables of all sample. The absence of a circle in the grid indicates that the correlation is not statistically significant and was therefore not represented. Values tending towards dark red indicate negative correlations; values tending towards dark blue indicate a positive correlation. The specific value of the correlation is written inside every circle and that value is proportionate with the size of the circle

Correlation analysis was first performed on the entire study population confirming the literature on apathy and neurocognitive tests in Alzheimer-type degenerative dementia. The correlation matrix with only the significant values can be found in Fig. 2.

The total Apathy scale score tends to decrease with increasing global cognitive level and education. High Apathy scores, on the other hand, correlate with patients who have a more severe diagnosis, and comorbidities, follow drug treatment, do neurocognitive rehabilitation, and have higher Hamilton Scale scores.

**Fig. 3:**
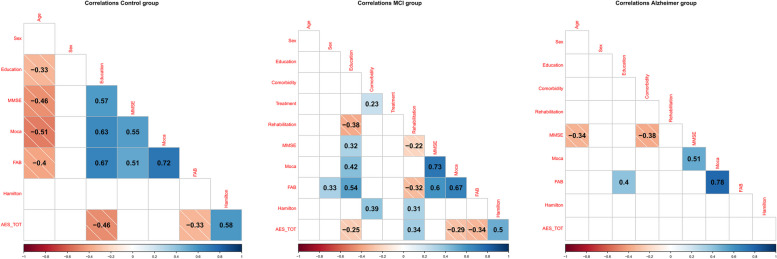
Spearman correlation tables dividing the three experimental groups. The absence of a circle in the grid indicates that the correlation is not statistically significant and was therefore not represented. Values tending towards dark red indicate negative correlations; values tending towards dark blue indicate a positive correlation. The specific value of the correlation is written inside every circle and that value is proportionate with the size of the circle

Analysing the correlations by separating the three conditions (Fig. 3), important differences can be noticed: the Control group and the MCI group maintain the trend observed globally in which apathy increases with increasing depressive symptomatology and decreases with increasing education, and particularly the FAB score (rather than the MoCA and MMSE). In the group with mild AD, on the other hand, the correlation analysis does not provide any information on apathy symptomatology.

The same was observed for the regression analysis. A robust regression method was used in R as the residuals were not normally distributed. The best-predicting Apathy model in each group was then chosen using Akaike’s criterion. In the Control group, the model (AIC = 371.22) predicted the construct of Apathy with an accuracy of 50% (R^2^ = 0.495; *p* < 0.001) using “Hamilton”, “Education”, and “MMSE” as predictors. On the other hand, in the MCI group, the model selected (AIC = 343.04) had a lower predicting strength of 26% (R^2^ = 0.263; *p* < 0.001) with “Hamilton”, “MMSE”, and “Age” as the principal predictors. The accuracies of the two models are considered acceptable in the field of psychological sciences [62].

The robust regression applied to the mild Alzheimer's group failed in returning a predictive model for the Apathy construct. A further analysis performed only in the mild Alzheimer’s group involved a K-MEANS unsupervised classification/clustering technique which was unable to give a satisfactory breakdown reflecting the division of the three diagnoses and a Principal Component Analysis which, in turn, showed much overlap between most of the components. These results did not allow for more specific differentiating information and finding ‘unique’ patterns of the Apathy construct in the three groups. Network models were run precisely to overcome these limitations.

Complete information can be found in the Supplementary Material where all the R-codes used for the analyses and the results with graphs are provided.

### Network analysis results

**Fig. 4:**
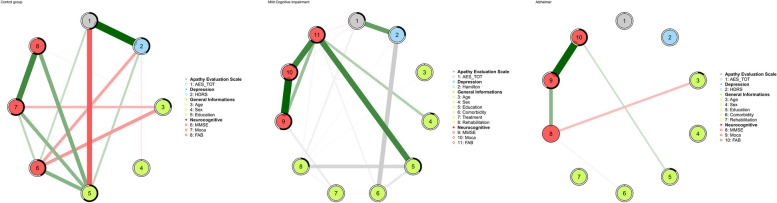
network analysis of the three groups (Control on the left, MCI in the middle and mild Alzheimer on the right) keeping the Total Score of Apathy Evaluation Scale. Nodes represent the variables used to calculate the network model; Lines between nodes are the edges: partial correlations between variables (thicker the edge, greater the correlation value); green edges: positive correlations; red edges: negative correlations; black-coloured edges: partial correlations between dichotomous and continuous variables. Predictability value was represented by a ring around each node. The blacker the ring, the more variance of the variable is explained by the other connected nodes

The Network analysis conducted in the three different groups showed differences in the way that the Apathy construct was related to the other demographic and neurocognitive variables. The Apathy Evaluation Scale has been represented item-per-item in the network analysis and the total score was not included to avoid the problem of spurious correlations. Before doing so, three more models were represented (Fig. 4) in which all the variables were positioned in a “circle” layout. The control group network in Fig. 4 (left) illustrates well-established associations between demographic variables and neurocognitive tests as documented in the literature. For the Control group, we observe that education level is positively associated with scores on global cognitive function assessments and the FAB, while age is negatively related to these scores, with a stronger effect on the MoCA and MMSE than on the FAB. This set of associations shifts significantly within the MCI patient network, where the FAB remains the only neurocognitive test positively associated with both education level and sex Male patients tended to perform better than female patients, consistent with recent FAB recalibration data [63]. As observed in other studies [64] using different scales to evaluate apathy, this construct shows a negative association with education level. In our study, this relationship is noted only in the Control group. This first analysis confirms the robust regression for the Control, MCI, and mild Alzheimer group because, for the last one, no pair-wise correlation was found between the Apathy Evaluation Scale node and the other variables. Below are the results of the network analysis for each group investigated.

Since after bootstrapping each model, the Betweenness centrality index was the least stable, Strength Centrality and Expected Influence were used as parameters to assess the greater importance of one node over another in the network models.

#### Control group network

**Fig. 5:**
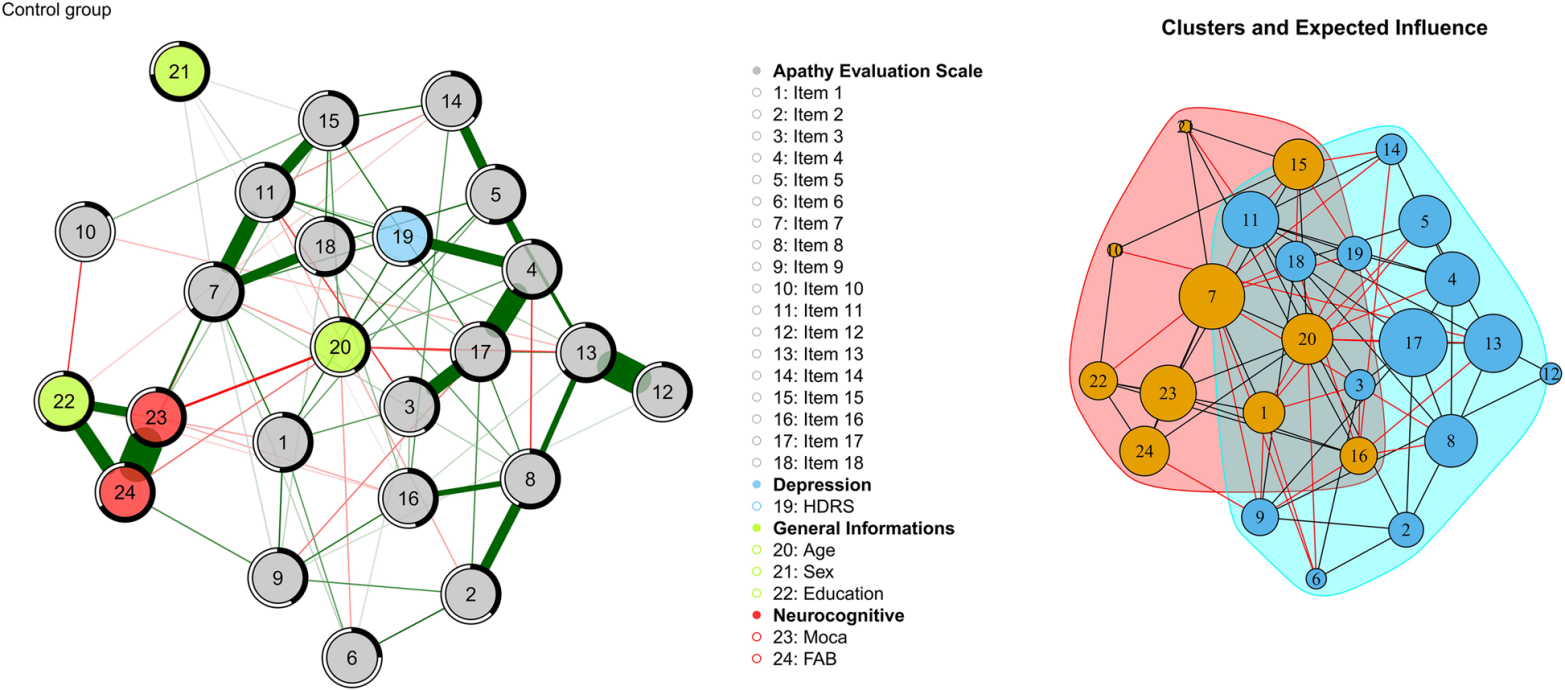
Part A: Network of the Control group. Nodes represent the variables used to calculate the network model; Lines between nodes are the edges: partial correlations between variables (thicker the edge, greater the correlation value); green edges: positive correlations; red edges: negative correlations; black-coloured edges: partial correlations between dichotomous and continuous variables. Predictability value was represented by a ring around each node. The blacker the ring, the more variance of the variable is explained by the other connected nodes. Part B: representation of the clusters identified through the walktrap algorithm. Black edges: intra-cluster connections; red edges: inter-cluster connections. The size of the nodes reflects the value of “Expected influence”. The bigger the higher

The Network of the Control Group (Fig. 5) resulted in the densest (density index = 0.278, number of edges = 77) between the three groups and with the lowest number of clusters (*n* = 2) detected by the *walktrap* algorithm. It is therefore a network that can adapt and change; not static, but one in which disturbances (positive and negative) spread faster. Furthermore, there is no parcellation of information due to many clusters; this leads to nodes being very much in contact and interacting with each other from multiple paths at the same time. Graphically the nodes representing the Apathy Evaluation Scale (nodes from 1 to 18) occupied the right part of the network, leaving neurocognitive variables and the general information (demographics) in the left part. The nodes with the highest Expected Influence centrality are 7 (item 7: S/he approaches life with intensity; EI = 0.963) and 17 (item 17: S/he has initiative; EI = 1), followed by node 13 (item 13: Getting together with friends is important to him/her; EI = 0.885), node 11 (item 11: S/he is less concerned about her/his problems than s/he should be; EI = 0.830 and node 20 (Age) which is the node in the most central position.

Additionally, nodes 7 and 17 have no edges between them and belong to two different clusters (Fig. 2, part B). Node 19 (HDRS) has really few connections with all the other variables of the Network and is one of the less important, meaning that the construct of Apathy depression symptomatology has a low impact in this group. Previous statistical analyses have indeed shown that there is a clear difference between Apathy and Depression in their prevalence among the three groups.

#### MCI group network

**Fig. 6:**
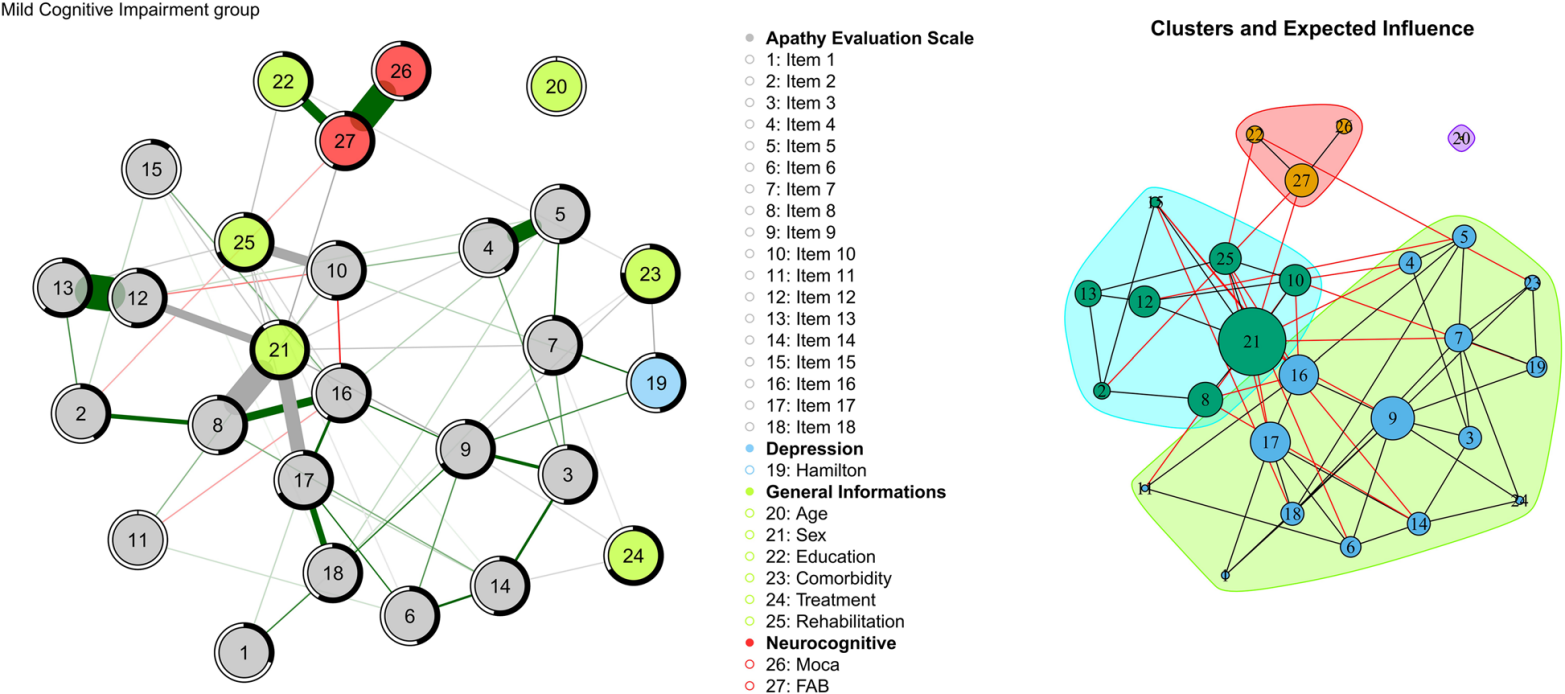
Part A: Network of the MCI group. Nodes represent the variables used to calculate the network model; Lines between nodes are the edges: partial correlations between variables (thicker the edge, greater the correlation value); green edges: positive correlations; red edges: negative correlations; black-coloured edges: partial correlations between dichotomous and continuous variables. Predictability value was represented by a ring around each node. The blacker the ring, the more variance of the variable is explained by the other connected nodes. Part B: representation of the clusters identified through the walktrap algorithm. Black edges: intra-cluster connections; red edges: inter-cluster connections. The size of the nodes reflects the value of “Expected influence”. The bigger the higher

MCI patients Network (Fig. 6) had a smaller number of edges, 65, and a lower density index: of 0.185. Increased compared to the control group was also the number of clusters identified: 3 and 1 node (20-Age) isolated from the network that forms a cluster itself (Fig. 3, part B). The nodes of the AES scale are still compact although several changes were seen possibly due to the inclusion of new dichotomous variables that were not present in the control group: Comorbidity (node 23), Treatment (node 24), and Rehabilitation (node 25). The two neurocognitive nodes (nodes 27–28) and the depression scale (node 19) retain a minimal influence on the network dynamics. Compared to the previous network, node 20 (Age) also loses importance completely and is replaced by node 21 (Sex). The nodes with the highest values of centrality measures are: 21 (Sex, EI = 1), 9 (item 9: S/he spends time doing things that interest her/him.; EI = 0.640), 16 (item 16: Getting things done during the day is important to her/him, BC = 0.584, and 17 (item 17: S/he has initiative, EI = 0.592).

#### Mild Alzheimer Disease’s group network

**Fig. 7:**
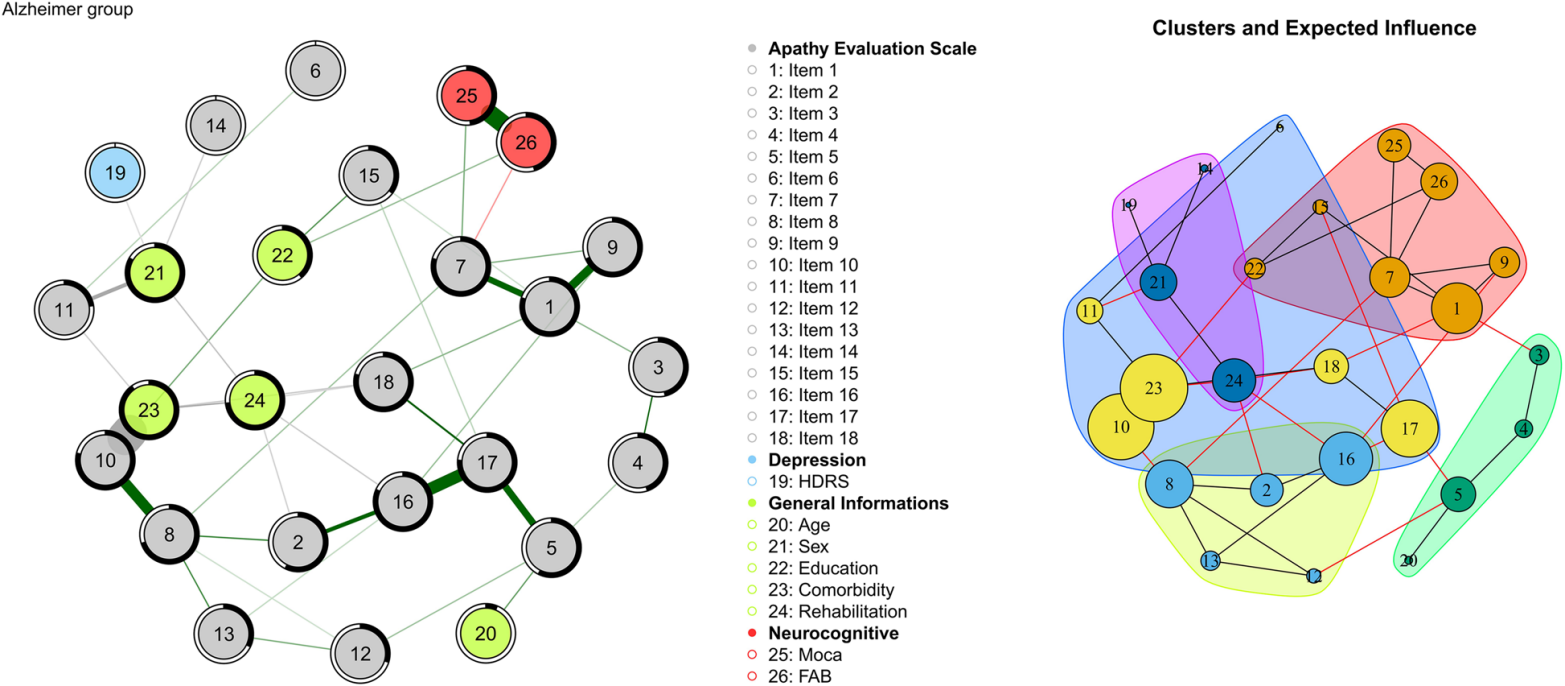
Part A: Network of the Mild Alzheimer group. Nodes represent the variables used to calculate the network model; Lines between nodes are the edges: partial correlations between variables (thicker the edge, greater the correlation value); green edges: positive correlations; red edges: negative correlations; black-coloured edges: partial correlations between dichotomous and continuous variables. Predictability value was represented by a ring around each node. The blacker the ring, the more variance of the variable is explained by the other connected nodes. Part B: representation of the clusters identified through the walktrap algorithm. Black edges: intra-cluster connections; red edges: inter-cluster connections. The size of the nodes reflects the value of “Expected influence”. The bigger the higher

Mild Alzheimer group’s Network (Fig. 7) had the lowest number of edges, 41, with a density index of 0.126. The network is less diffuse and more specific; all the various groups (AES items, demographic information, and neurocognitive variables) are separated from each other into small worlds. Cluster analysis now shows 6 clusters but only two of them (light blue and red) contain variables with high scores for centrality (Fig. 7, part B). In this model, the centrality analyses had a correlation index greater than 0.5 up to a 40% reduction. In contrast, betweenness centrality was highly unstable.

The two nodes that are more important are: node 23, Comorbidity (EI = 1) and node 10 (item 10: Someone has to tell her/him what to do each day; EI = 0.979) which together with node 24 (Rehabilitation) form a separate cluster. In this cluster, the aspect of abulia takes shape and becomes central, a characteristic feature of apathy in Alzheimer's disease and here linked to the presence of comorbidity and node 8 (item 8: Seeing a job through to the end is important to her/him).

Apart from these, other nodes that share comparable centrality values are 17, 16, 1 and 8. All these AES Scale items share the common characteristic of belonging to the Cognitive Apathy score.

## Discussion

This study sought to deepen our understanding of how symptoms and demographic factors interact across various stages of cognitive decline, focusing especially on the role of apathy. Due to the complexity of this subject, we conducted a variety of analyses.

Firstly, most variables in the MCI and mild AD groups were not normally distributed. This can be explained both because the control group is the largest, and because patients with complex diseases tend to have greater symptomatic variability. Our results are in line with existing studies and highlight that as individuals approach AD, they exhibit increased severity in terms of lower educational levels, reduced cognitive test performance, and greater occurrence of comorbid conditions, depression, and apathy. Notably, individuals with MCI showed higher levels of depression compared to those with mild AD, who, conversely, demonstrated greater apathy. This reinforces the understanding that depression and apathy are two separate clinical aspects of cognitive impairment, necessitating independent measurement and treatment. Careful consideration is required as one condition can mimic the effects of the other in such disease [65]. Also, executive dysfunction did not differ significantly between the MCI and mild AD groups. Our MCI group’s average score on the FAB was below the threshold, indicating a possible sampling bias. This bias might have led to the inclusion of MCI patients with non-memory-based initial symptoms, who may not necessarily progress to AD. Nonetheless, symptoms of executive dysfunction are crucial and must be closely monitored to lower the risk of progression to dementia due to the reduction of the patient’s independence [66].

Our findings align with existing research and show a significant increase in apathy levels linearly correlated with clinical deterioration. However, when examining the three groups separately, both controls and older individuals with MCI displayed similar patterns of apathy. This supports the theory that there is a critical period, from healthy cognitive function to MCI, during which the risk of progressing to more severe conditions can be mitigated through targeted interventions [67]. Conversely, within the mild Alzheimer’s Disease group, we observed a breakdown in relationships and information, with no predictive models for apathy emerging. Additionally, attempts to identify patterns in the data using automatic search algorithms did not yield distinct behavioural differences among the variables in the mild Alzheimer’s group.

Thus, we conducted a Network Analysis to gain a deeper understanding of the clinical characteristics of participants in the Control, MCI, and mild AD groups. Initially, we evaluated apathy using a single overall score. Through this approach, our network analysis validated the findings of regression analyses, identifying Hamilton (depression scale), Education, and MMSE as significant predictors of apathy in the control group; Hamilton, MMSE, and Age in the MCI group; with no significant predictors found in the mild AD group. These insights highlight a notable shift in the demographic factors influencing apathy from healthy individuals to those with MCI. Education emerges as a modifiable risk factor that can be targeted to mitigate apathy before cognitive decline begins, while age, an unmodifiable factor, underscores the progressive nature of these conditions. For individuals with healthy cognition, there is greater potential to prevent apathy by focusing on mood, education, and cognitive engagement. In contrast, for those in the initial stages of cognitive decline, interventions might need to concentrate more on mood and cognitive engagement. Nevertheless, independent of cognitive status, monitoring for depressive symptoms remains a critical aspect of care. Given the absence of predictive models in mild AD, individual items of the AES scale were integrated into subsequent networks. The network of the control group (Fig. 5) emerged as the densest among the three groups, with the lower number of clusters, therefore the most non-specific in which there is a high degree of integration between psychological constructs, apathy components, and demographic variables that may affect these. Consequently, this predicts greater adaptability and responsiveness to environmental factors and heightened susceptibility to both positive and negative triggers. The close graphical positioning of the AES nodes in relation to neurocognitive and demographic variables was particularly striking. The strong link between apathy and cognitive performance is consistent with previous research [26, 68], which has established a solid relationship between the two, especially concerning intellectual curiosity in healthy older adults. Furthermore, in the control group, it was found that the most important nodes belonged to all three components of apathy. This implies that in the healthy population, a worsening of this symptomatology or changes in the network can result from both emotional, cognitive, and behavioural/social aspects.

The most central AES nodes were the desire to learn, acquire new information, take initiative, and live intensely, while the least central, only acquiring some importance in the Alzheimer network, was node 10: “Someone has to tell me what to do each day”. In fact, in AD, apathy also occurs in the form of abulia [69]. More importantly, by moving from one group to the other, node 10 moves from a completely peripheral position to being one of the most important nodes in the Alzheimer's Disease Network. Moreover, this node is extremely dependent on node 23 (Comorbidity) and node 8. Interestingly, item 8 of the Apathy Evaluation Scale refers to an interest in following a job/objective all the way through and seeing it through to completion. Increasingly, in people with Alzheimer's disease, the environment tends to remove the ability to perform tasks independently sooner rather than later [70]. While this leads to greater peace of mind for the caregiver and institutions, it also makes the patient's apathy and abulia progress faster.

The analysis of the MCI network (Fig. 6) revealed notable differences compared to the control group. Despite adding new binary variables, the network for MCI showed a lower density index, indicating weaker interconnectivity and a reduced propensity for change. Moreover, the centrality of age decreased, while sex took a more prominent role becoming a key element of the network. This observation highlights the importance of factors related to sex in the risk of progressing to dementia. Identifying sex as a key factor could serve as an early marker for stratifying dementia risk [71, 72], which in turn could help create more personalized and effective treatment strategies. By including sex-specific variables in the assessment of dementia risk, we can enhance the precision of risk evaluations and design interventions that more accurately target individual risk profiles.

In comparison, the network analysis for mild AD patients (referenced in Fig. 7) showed a marked decrease in both network density and the number of clusters. This reduction signifies a loss of functional specificity and adaptability within the network. As a result, there are fewer options for targeted therapeutic interventions for AD patients. As comorbidity plays a predominant role in the network, the presence of depressive and anxious symptoms or other pathologies must be closely monitored and treated effectively, as they can affect rehabilitation, which in turn is the main means by which one can influence apathetic symptoms in this group. However, certain aspects, such as approaching life with intensity, and having initiative and motivation remain consistent and vital. This could provide a fundamental basis and a clear indication for clinicians to establish short-term daily goals.

The most interesting aspect remains the transformation and increase of clusters between the three stages, almost signifying an increase in the specificity and compartmentalization of information requiring more variables and information in each cluster to bring out the underlying latent factors. Compared to the study by Tosi et al., 2024 [73], this article found a higher number of network communities as cognitive impairment increased, along with a decrease in connection density, suggesting fragmentation rather than non-specificity. This phenomenon may be due to two main factors: first, the inclusion of demographic, medical, and treatment-related variables in the network for both clinically diagnosed groups [74, 75], reflecting their significant influence on apathy. Second, apathy appears to manifest greater complexity and individual variation in the Alzheimer’s disease group compared to those with mild impairment or healthy individuals.

This study suffered from some limitations, notably the lack of a longitudinal design. Even if this shortcoming was addressed by including three different samples with distinct and progressive cognitive status, it was not possible to infer any role played by specific variables over time, as not all Control developed MCI, and not all MCI developed AD. Future research should emphasize the inclusion of retesting the same participants to verify changes in clinical manifestations over time. The sample size should be mentioned as well, especially for the mild AD group. However, this limitation was addressed by implementing analyses that took this number into account, especially regarding network analyses that are sensitive to sample size. Cross-validation techniques were used and the stability of the centrality analyses of each network model was checked with multiple models from bootstrap samples. The indices were then correlated with those of the original sample.

## Conclusions

In sum, our research underscores the critical role of apathy in cognitive impairment, enriching our comprehension of its interaction with clinical dimensions across healthy individuals, those with MCI, and mild AD patients. Through Network Analysis, we have brought fresh insights related to the crucial importance of internal factors such as initiative and interest, especially in healthy and MCI subjects, and their continued significance in mild AD patients. These findings advocate for behavioural treatments aimed at enhancing motivational engagement, assisting individuals in engaging with daily activities [76], and not taking away the possibility for patients to demonstrate that they can perform certain activities on their own. Additionally, our observations in the MCI cohort suggest that apathy could serve as an early indicator of the transition to dementia, highlighting the need for future research to focus on disease-modifying drug interventions to prevent the transition from MCI to AD [77]. In this context, longitudinal studies will clarify the specific role played by each variable across different stages of deterioration, as this article suffered from the limitations of cross-sectional studies and did not allow to draw this kind of conclusion.

Network Analysis further illuminates the criticality of certain symptoms in forming the apathetic pattern, paving the way for future research to identify the most informative psychometric tool items. This approach promises to refine screening and diagnostic protocols, optimizing the balance between administration time, participant fatigue, and clinical efficacy.

Moreover, our study underlines the importance of demographic factors. This points to the necessity for future studies to delve into sex differences in cognitive impairment dynamics within MCI [78]. This approach aims to suggest a new frontier for research in understanding MCI’s clinical dimensions and then help to select MCI patients with biological markers of preclinical AD for future clinical trials with disease-modifying drugs (e.g. immunotherapy) [79]. Further analysis will combine this with new Network Intervention Analysis approaches to map via networks the interactions of drugs with variables describing the neurocognitive, affective, and environmental interaction aspects of the disease [80].

Moreover, additional studies should be conducted to elucidate the specific role of apathy in AD in mild AD patients before starting any treatment with cholinesterase inhibitors (as planned in this study with a small sample). This will also help to understand whether they may affect apathetic symptoms, as recent studies have not shown a definite correlation [81, 82].

## Supplementary Information

Supplementary Material 1.

Supplementary Material 2.

Supplementary Material 3.

Supplementary Material 4.

## Data Availability

The datasets used and/or analyzed during the current study are available from the corresponding author on reasonable request. The materials, including the R code used for statistical and network analyses and image generation, are provided as supplementary material. Additionally, the English and Italian versions of the Apathy Evaluation Scale—Clinician version used are also supplied.

## Abbreviations

MCI: Mild Cognitive Impairment
AD: Alzheimer’s Disease
NIA: National Institute of Aging
MMSE: Mini Mental State Examination
MoCA: Montreal Cognitive Assessment
FAB: Frontal Assessment Battery
HDRS: Hamilton Depression Rating Scale
AES-C: Apathy Evaluation Rating Scale – Clinician version
GMM: Graphical mixed model
SC: Strength Centrality
BC: Betweenness Centrality
EI: Expected Influence
AIC: Akaike’s Information Criterion

## References

[1] Fahed M, Steffens DC. Apathy: Neurobiology. Assessment and Treatment Clin Psychopharmacol Neurosci. 2021;19:181–9. 10.9758/cpn.2021.19.2.181. 33888648 10.9758/cpn.2021.19.2.181 PMC8077060

[2] Marin RS. Apathy: A neuropsychiatric syndrome. J Neuropsychiatry Clin Neurosci. 1991;3. 10.1176/jnp.3.3.243. 10.1176/jnp.3.3.243 1821241

[3] Robert P, Onyike CU, Leentjens AFG. Proposed diagnostic criteria for apathy in Alzheimer’s disease and other neuropsychiatric disorders. Eur Psychiatry. 2009;24. 10.1016/j.eurpsy.2008.09.001. 10.1016/j.eurpsy.2008.09.001 19201579

[4] P Robert. KL Lanctôt, L Agüera-Ortiz. Is it time to revise the diagnostic criteria for apathy in brain disorders? The 2018 international consensus group. Eur Psychiatry. 2018;5410.1016/j.eurpsy.2018.07.008 10.1016/j.eurpsy.2018.07.008 30125783

[5] Guercio BJ, Donovan NJ, Munro CE. The Apathy Evaluation Scale: A Comparison of Subject, Informant, and Clinician Report in Cognitively Normal Elderly and Mild Cognitive Impairment. J Alzheimer’s Dis 2015;47. 10.3233/JAD-150146. 10.3233/JAD-150146 PMC4602169 26401564

[6] Groeneweg-Koolhoven I, Ploeg M, Comijs HC. Apathy in early and late-life depression. J Affect Disord 2017;223. 10.1016/j.jad.2017.07.022. 10.1016/j.jad.2017.07.022 28734148

[7] Brown DS, Barrett MJ, Flanigan JL, Sperling SA. Clinical and demographic correlates of apathy in Parkinson’s disease. J Neurol 2019;266. 10.1007/s00415-018-9166-3. 10.1007/s00415-018-9166-3 30604055

[8] RM L, FE M, T D, C B. Patterns and persistence of behavioural and psychological symptoms in those with cognitive impairment: the importance of apathy. Int J Geriatr Psychiatry 2017;32. 10.1002/gps.4464. 10.1002/gps.4464 27017917

[9] Landes AM, Sperry SD, Strauss ME. Prevalence of Apathy, Dysphoria, and Depression in Relation to Dementia Severity in Alzheimer’s Disease. J Neuropsychiatry Clin Neurosci. 2005;17:342–9. 10.1176/jnp.17.3.342. 16179656 10.1176/jnp.17.3.342

[10] Nobis L, Husain M. Apathy in Alzheimer’s disease. Curr Opin Behav Sci. 2018;22:7–13. 10.1016/j.cobeha.2017.12.007. 30123816 10.1016/j.cobeha.2017.12.007 PMC6095925

[11] Johansson M, Stomrud E, Lindberg O. Apathy and anxiety are early markers of Alzheimer’s disease. Neurobiol Aging 2020;85. 10.1016/j.neurobiolaging.2019.10.008. 10.1016/j.neurobiolaging.2019.10.008 31735378

[12] Johansson M, Stomrud E, Johansson PM. Development of Apathy, Anxiety, and Depression in Cognitively Unimpaired Older Adults: Effects of Alzheimer’s Disease Pathology and Cognitive Decline. Biol Psychiatry 2022;92. 10.1016/j.biopsych.2022.01.012. 10.1016/j.biopsych.2022.01.012 35346458

[13] Sun L, Li W, Li G, Xiao S. Prefrontal Aβ pathology influencing the pathway from apathy to cognitive decline in non-dementia elderly. Transl Psychiatry 2021;11. 10.1038/s41398-021-01653-8. 10.1038/s41398-021-01653-8 PMC8523745 34663799

[14] Richard E, Schmand B, Eikelenboom P. Symptoms of apathy are associated with progression from mild cognitive impairment to Alzheimer’s disease in non-depressed subjects for the Alzheimer’s disease neuroimaging initiative. Dement Geriatr Cogn Disord 2012;33. 10.1159/000338239. 10.1159/000338239 22722671

[15] Boyle PA, Malloy PF, Salloway S, Cahn-Weiner DA, Cohen R, Cummings JL. Executive dysfunction and apathy, predict functional impairment in Alzheimer disease. Am J Geriatr Psychiatry 2003;11. 10.1097/00019442-200303000-00012. 12611751

[16] Starkstein SE, Jorge R, Mizrahi R, Robinson RG. A prospective longitudinal study of apathy in Alzheimer’s disease. J Neurol Neurosurg Psychiatry 2006;77. 10.1136/jnnp.2005.069575. 10.1136/jnnp.2005.069575 PMC2117400 16361584

[17] Fan Z, Wang L, Zhang H. Apathy as a Risky Neuropsychiatric Syndrome of Progression From Normal Aging to Mild Cognitive Impairment and Dementia: A Systematic Review and Meta-Analysis. Front Psychiatry 2021;12. 10.3389/fpsyt.2021.792168. 10.3389/fpsyt.2021.792168 PMC8721876 34987434

[18] van Dalen JW, van Wanrooij LL, Moll van Charante EP, Brayne C, van Gool WA, Richard E. Association of Apathy With Risk of Incident Dementia: A Systematic Review and Meta-analysis. JAMA Psychiatry 2018;75:1012–21. 10.1001/jamapsychiatry.2018.1877. 10.1001/jamapsychiatry.2018.1877 PMC6233800 30027214

[19] Ma L. Depression, Anxiety, and Apathy in Mild Cognitive Impairment: Current Perspectives. Front Aging Neurosci. 2020;12:9. 10.3389/fnagi.2020.00009. 32082139 10.3389/fnagi.2020.00009 PMC7002324

[20] Lechowski L, Benoit M, Chassagne P. Persistent apathy in Alzheimer’s disease as an independent factor of rapid functional decline: The REAL longitudinal cohort study. Int J Geriatr Psychiatry 2009;24. 10.1002/gps.2125. 10.1002/gps.2125 18814198

[21] García-Ramos R, Villanueva C, Val J, Matías-Guíu J. Apatía en la enfermedad de Parkinson. Neurologia 2010;25. 10.1016/S0213-4853(10)70021-9. 20388460

[22] Breitve MH, Brønnick K, Chwiszczuk LJ, Hynninen MJ, Aarsland D, Rongve A. Apathy is associated with faster global cognitive decline and early nursing home admission in dementia with Lewy bodies. Alzheimers Res Ther 2018;10. 10.1186/s13195-018-0416-5. 10.1186/s13195-018-0416-5 PMC6098842 30121084

[23] Andrews SC, Langbehn DR, Craufurd D. Apathy predicts rate of cognitive decline over 24 months in premanifest Huntington’s disease. Psychol Med 2021;51. 10.1017/S0033291720000094. 10.1017/S0033291720000094 32063235

[24] Mulin E, Leone E, Dujardin K. Diagnostic criteria for apathy in clinical practice. Int J Geriatr Psychiatry 2011;26. 10.1002/gps.2508. 10.1002/gps.2508 20690145

[25] Robert PH, Berr C, Volteau M. Importance of lack of interest in patients with mild cognitive impairment. Am J Geriatr Psychiatry 2008;16. 10.1097/JGP.0b013e31817e73db. 10.1097/JGP.0b013e31817e73db 18757769

[26] Montoya-Murillo G, Ibarretxe-Bilbao N, Peña J, Ojeda N. The impact of apathy on cognitive performance in the elderly. Int J Geriatr Psychiatry 2019;34. 10.1002/gps.5062. 10.1002/gps.5062 PMC6594084 30672026

[27] Husain M, Roiser JP. Neuroscience of apathy and anhedonia: a transdiagnostic approach. Nat Rev Neurosci. 2018;19:470–84. 10.1038/s41583-018-0029-9. 29946157 10.1038/s41583-018-0029-9

[28] Ilardi CR, Sannino M, Federico G, Cirillo MA, Cavaliere C, Iavarone A, et al. The Starkstein Apathy Scale-Italian Version: An Update. J Geriatr Psychiatry Neurol. 2024;37:379–86. 10.1177/08919887241227404. 38233366 10.1177/08919887241227404

[29] Lechowski L, Stampa M, Tortrat D. Predictive factors of rate of loss of autonomy in Alzheimer’s disease patients. A prospective study of the REAL.FR cohort. J Nutr Health Aging. 2005;9:100–4. 15791353

[30] Bock MA, Bahorik A, Brenowitz WD, Yaffe K. Apathy and risk of probable incident dementia among community-dwelling older adults. Neurology 2020;95. 10.1212/WNL.0000000000010951. 10.1212/WNL.0000000000010951 PMC7836653 33055276

[31] Esposito F, Rochat L, Linden AC, Lekeu F, Charnallet A, Linden M. Apathy in aging: Are lack of interest and lack of initiative dissociable? Arch Gerontol Geriatr 2014;58. 10.1016/j.archger.2013.09.002. 10.1016/j.archger.2013.09.002 24135627

[32] Sarti P, Colliva C, Varrasi S, Guerrera CS, Platania GA, Boccaccio FM, et al. A network study to differentiate suicide attempt risk profiles in male and female patients with major depressive disorder. Clin Psychol Psychother. 2023. 10.1002/cpp.2924. 37922512 10.1002/cpp.2924

[33] Platania GA, Guerrera CS, Sarti P, Varrasi S, Pirrone C, Popovic D, et al. Predictors of functional outcome in patients with major depression and bipolar disorder: A dynamic network approach to identify distinct patterns of interacting symptoms. PLoS ONE. 2023;18:e0276822. 10.1371/journal.pone.0276822. 36791083 10.1371/journal.pone.0276822 PMC9931103

[34] Scarponi D, Sarti P, Rivi V, Colliva C, Marconi E, Pession A, et al. Emotional, Behavioral, and Physical Health Consequences in Caregivers of Children with Cancer: A Network Analysis Differentiation in Mothers’ and Fathers’ Reactivity. Cancers. 2023;15:3496. 10.3390/cancers15133496. 37444606 10.3390/cancers15133496 PMC10340596

[35] Van Wanrooij LL, Borsboom D, Moll Van Charante EP, Richard E, Van Gool WA. A network approach on the relation between apathy and depression symptoms with dementia and functional disability. Int Psychogeriatr 2019;31:1655–63. 10.1017/S1041610218002387. 10.1017/S1041610218002387 30782219

[36] Montine TJ, Phelps CH, Beach TG, Bigio EH, Cairns NJ, Dickson DW, et al. National Institute on Aging–Alzheimer’s Association guidelines for the neuropathologic assessment of Alzheimer’s disease: a practical approach. Acta Neuropathol (Berl). 2012;123:1–11. 10.1007/s00401-011-0910-3. 22101365 10.1007/s00401-011-0910-3 PMC3268003

[37] Albert MS, DeKosky ST, Dickson D, Dubois B, Feldman HH, Fox NC, et al. The diagnosis of mild cognitive impairment due to Alzheimer’s disease: recommendations from the National Institute on Aging-Alzheimer’s Association workgroups on diagnostic guidelines for Alzheimer’s disease. Alzheimers Dement J Alzheimers Assoc. 2011;7:270–9. 10.1016/j.jalz.2011.03.008. 10.1016/j.jalz.2011.03.008 PMC3312027 21514249

[38] McKhann GM, Knopman DS, Chertkow H, Hyman BT, Jack CR, Kawas CH, et al. The diagnosis of dementia due to Alzheimer’s disease: recommendations from the National Institute on Aging-Alzheimer’s Association workgroups on diagnostic guidelines for Alzheimer’s disease. Alzheimers Dement J Alzheimers Assoc. 2011;7:263–9. 10.1016/j.jalz.2011.03.005. 10.1016/j.jalz.2011.03.005 PMC3312024 21514250

[39] Magni E, Binetti G, Bianchetti A, Rozzini R, Trabucchi M. Mini-Mental State Examination: a normative study in Italian elderly population. Eur J Neurol. 1996;3:198–202. 10.1111/j.1468-1331.1996.tb00423.x. 21284770 10.1111/j.1468-1331.1996.tb00423.x

[40] Folstein MF, Folstein SE, McHugh PR. Mini-mental state”. A practical method for grading the cognitive state of patients for the clinician. J Psychiatr Res 1975;12:189–98. 10.1016/0022-3956(75)90026-6. 10.1016/0022-3956(75)90026-6 1202204

[41] Nota 85 n.d. https://www.aifa.gov.it/nota-85 (accessed July 24, 2024).

[42] Diagnostic and Statistical Manual of Mental Disorders. DSM Libr n.d. https://dsm.psychiatryonline.org/doi/book/10.1176/appi.books.9780890425787 (accessed June 17, 2024).

[43] ICD-11 for Mortality and Morbidity Statistics n.d. https://icd.who.int/browse/2024-01/mms/en (accessed June 6, 2024).

[44] Bosco A, Spano G, Caffò AO, Lopez A, Grattagliano I, Saracino G, et al. Italians do it worse. Montreal Cognitive Assessment (MoCA) optimal cut-off scores for people with probable Alzheimer’s disease and with probable cognitive impairment. Aging Clin Exp Res 2017;29:1113–20. 10.1007/s40520-017-0727-6. 10.1007/s40520-017-0727-6 28155182

[45] Ilardi CR, Menichelli A, Michelutti M, Cattaruzza T, Manganotti P. Optimal MoCA cutoffs for detecting biologically-defined patients with MCI and early dementia. Neurol Sci. 2023;44:159–70. 10.1007/s10072-022-06422-z. 36169756 10.1007/s10072-022-06422-z PMC9816212

[46] Salute M della. Dati epidemiologici n.d. https://www.salute.gov.it/portale/demenze/dettaglioContenutiDemenze.jsp?id=2402&area=demenze&menu=vuoto (accessed June 6, 2024).

[47] Nasreddine ZS, Phillips NA, Bédirian V, Charbonneau S, Whitehead V, Collin I, et al. The Montreal Cognitive Assessment, MoCA: a brief screening tool for mild cognitive impairment. J Am Geriatr Soc. 2005;53:695–9. 10.1111/j.1532-5415.2005.53221.x. 15817019 10.1111/j.1532-5415.2005.53221.x

[48] Santangelo G, Siciliano M, Pedone R, Vitale C, Falco F, Bisogno R, et al. Normative data for the Montreal Cognitive Assessment in an Italian population sample. Neurol Sci Off J Ital Neurol Soc Ital Soc Clin Neurophysiol. 2015;36:585–91. 10.1007/s10072-014-1995-y. 10.1007/s10072-014-1995-y 25380622

[49] Aiello EN, Esposito A, Gramegna C, Gazzaniga V, Zago S, Difonzo T, et al. The Frontal Assessment Battery (FAB) and its sub-scales: validation and updated normative data in an Italian population sample. Neurol Sci. 2022;43:979–84. 10.1007/s10072-021-05392-y. 34184168 10.1007/s10072-021-05392-y PMC8789707

[50] Fava G, Kellner R, Munari F, Pavan L. The Hamilton Depression Rating Scale in normals and depressives. Acta Psychiatr Scand. 1982;66:26–32. 10.1111/J.1600-0447.1982.TB00911.X. 7124430 10.1111/j.1600-0447.1982.tb00911.x

[51] Furneri G, Platania S, Privitera A, Martelli F, Smeriglio R, Razza G, et al. The Apathy Evaluation Scale (AES-C): Psychometric Properties and Invariance of Italian Version in Mild Cognitive Impairment and Alzheimer’s Disease. Int J Env Res Public Health. 2021;18:9597. 10.3390/ijerph18189597. 34574524 10.3390/ijerph18189597 PMC8467636

[52] Sedgewick AJ, Shi I, Donovan RM, Benos PV. Learning mixed graphical models with separate sparsity parameters and stability-based model selection. BMC Bioinformatics. 2016;17:S175. 10.1186/s12859-016-1039-0. 10.1186/s12859-016-1039-0 PMC4905606 27294886

[53] Epskamp S, Fried EI. A tutorial on regularized partial correlation networks. Psychol Methods. 2018;23:617–34. 10.1037/met0000167. 29595293 10.1037/met0000167

[54] Jung Y, Hu J. A K-fold averaging cross-validation procedure. J Nonparametric Stat. 2015;27:167–79. 10.1080/10485252.2015.1010532. 10.1080/10485252.2015.1010532 PMC5019184 27630515

[55] Epskamp S, Cramer AOJ, Waldorp LJ, Schmittmann VD, Borsboom D. qgraph: Network Visualizations of Relationships in Psychometric Data. J Stat Softw 2012;48:1–18. 10.18637/jss.v048.i04.

[56] Fruchterman TMJ, Reingold EM. Graph drawing by force-directed placement. Softw Pract Exp. 1991;21:1129–64. 10.1002/spe.4380211102.

[57] Haslbeck JMB, Waldorp LJ. How well do network models predict observations? On the importance of predictability in network models. Behav Res Methods. 2018;50:853–61. 10.3758/s13428-017-0910-x. 28718088 10.3758/s13428-017-0910-x PMC5880858

[58] Robinaugh DJ, Millner AJ, McNally RJ. Identifying Highly Influential Nodes in the Complicated Grief Network. J Abnorm Psychol. 2016;125:747–57. 10.1037/abn0000181. 27505622 10.1037/abn0000181 PMC5060093

[59] Csardi G, Nepusz T. The Igraph Software Package for Complex Network Research. Int J Complex Syst. 2006;1695:1-9. http://igraph.org.

[60] Pons P, Latapy M. Computing Communities in Large Networks Using Random Walks. In: Yolum pInar, Güngör T, Gürgen F, Özturan C, editors. Comput. Inf. Sci. - ISCIS 2005, Berlin, Heidelberg: Springer; 2005, p. 284–93. 10.1007/11569596_31.

[61] EpiCentro. Alzheimer’s disease n.d. https://www.epicentro.iss.it/en/alzheimer/ (accessed January 19, 2024).

[62] Ozili PK. The Acceptable R-Square in Empirical Modelling for. Soc Sci Res. 2022. 10.2139/ssrn.4128165.

[63] Garofalo E, Iavarone A, Chieffi S, Carpinelli Mazzi M, Gamboz N, Ambra FI, et al. Italian version of the Starkstein Apathy Scale (SAS-I) and a shortened version (SAS-6) to assess “pure apathy” symptoms: normative study on 392 individuals. Neurol Sci Off J Ital Neurol Soc Ital Soc Clin Neurophysiol. 2021;42:1065–72. 10.1007/s10072-020-04631-y. 10.1007/s10072-020-04631-y 32729011

[64] Ilardi CR, Chieffi S, Scuotto C, Gamboz N, Galeone F, Sannino M, et al. The Frontal Assessment Battery 20 years later: normative data for a shortened version (FAB15). Neurol Sci Off J Ital Neurol Soc Ital Soc Clin Neurophysiol. 2022;43:1709–19. 10.1007/s10072-021-05544-0. 10.1007/s10072-021-05544-0 34410549

[65] D’Iorio A, Santangelo G. Apathy and depression in amnestic and non-amnestic mild cognitive impairment. J Clin Exp Neuropsychol. 2022;44:103–8. 10.1080/13803395.2022.2074967. 35603512 10.1080/13803395.2022.2074967

[66] Junquera A, Garcia-Zamora E, Olazaran J, Parra MA, Fernandez-Guinea S. Role of Executive Functions in the Conversion from Mild Cognitive Impairment to Dementia. J Alzheimer’s Dis 2020;77. 10.3233/JAD-200586. 10.3233/JAD-200586 32741835

[67] Lissek V, Suchan B. Preventing dementia? Interventional approaches in mild cognitive impairment. Neurosci Biobehav Rev. 2021;122:143–64. 10.1016/j.neubiorev.2020.12.022. 33440197 10.1016/j.neubiorev.2020.12.022

[68] Lanctôt KL, Agüera-Ortiz L, Brodaty H, Francis PT, Geda YE, Ismail Z, et al. Apathy associated with neurocognitive disorders: Recent progress and future directions. Alzheimer’s Dement 2017;13. 10.1016/j.jalz.2016.05.008. 10.1016/j.jalz.2016.05.008 27362291

[69] Kesserwani H. Apathy Antedating and Evolving With Dementia: A Case Report and Insights Into Apathy as a Network Dysfunction. Cureus 2021;13. 10.7759/cureus.13802. 10.7759/cureus.13802 PMC8034504 33850671

[70] Wolfe SE, Greenhill B, Butchard S, Day J. The meaning of autonomy when living with dementia: A Q-method investigation. Dement Lond Engl. 2021;20:1875–90. 10.1177/1471301220973067. 10.1177/1471301220973067 PMC8369913 33372553

[71] Stephan BCM, Savva GM, Brayne C, Bond J, McKeith IG, Matthews FE. Optimizing mild cognitive impairment for discriminating dementia risk in the general older population. Am J Geriatr Psychiatry 2010;18. 10.1097/JGP.0b013e3181e0450d. 10.1097/jgp.0b013e3181e0450d 21491627

[72] Rosenberg PB, Mielke MM, Appleby BS, Oh ES, Geda YE, Lyketsos CG. The association of neuropsychiatric symptoms in MCI with incident dementia and alzheimer disease. Am J Geriatr Psychiatry 2013;21. 10.1016/j.jagp.2013.01.006. 10.1016/j.jagp.2013.01.006 PMC3428504 23567400

[73] Tosi G, Nigro S, Urso D, Spinosa V, Gnoni V, Filardi M, et al. The Network Structure of Cognitive Impairment: From Subjective Cognitive Decline to Alzheimer’s Disease. J Neurosci Off J Soc Neurosci. 2024;44: e1344232023. 10.1523/JNEUROSCI.1344-23.2023. 10.1523/JNEUROSCI.1344-23.2023 PMC11223460 38830757

[74] Ferguson C, Alzheimer’s Disease Neuroimaging Initiative. A network psychometric approach to neurocognition in early Alzheimer’s disease. Cortex J Devoted Study Nerv Syst Behav 2021;137:61–73. 10.1016/j.cortex.2021.01.002. 10.1016/j.cortex.2021.01.002 33607345

[75] Ferguson C. Supplementary analyses to “A network psychometric approach to neurocognition in early Alzheimer’s disease”: Differential variability, community structure, and statistical test 2023. 10.31234/osf.io/eshgr.

[76] Manera V, Abrahams S, Agüera-Ortiz L, Bremond F, David R, Fairchild K, et al. Recommendations for the Nonpharmacological Treatment of Apathy in Brain Disorders. Am J Geriatr Psychiatry. 2020;28:410–20. 10.1016/j.jagp.2019.07.014. 31495772 10.1016/j.jagp.2019.07.014

[77] Bogdan A, Manera V, Koenig A, David R. Pharmacologic Approaches for the Management of Apathy in Neurodegenerative Disorders. Front Pharmacol. 2020;10:1581. 10.3389/fphar.2019.01581 PMC6989486 32038253

[78] Ferretti MT, Iulita MF, Cavedo E, Chiesa PA, Dimech AS, Chadha AS, et al. Sex differences in Alzheimer disease — The gateway to precision medicine. Nat Rev Neurol 2018;14. 10.1038/s41582-018-0032-9. 10.1038/s41582-018-0032-9 29985474

[79] Bradshaw AC, Georges J. Anti-Amyloid Therapies for Alzheimer’s Disease: An Alzheimer Europe Position Paper and Call to Action. J Prev Alzheimers Dis 2024;11:265–73. 10.14283/jpad.2024.37. 10.14283/jpad.2024.37 38374732

[80] Guerrera CS, Platania GA, Boccaccio FM, Sarti P, Varrasi S, Colliva C, et al. The dynamic interaction between symptoms and pharmacological treatment in patients with major depressive disorder: the role of network intervention analysis. BMC Psychiatry. 2023;23:885. 10.1186/s12888-023-05300-y. 38017462 10.1186/s12888-023-05300-y PMC10683186

[81] Steffens DC, Fahed M, Manning KJ, Wang L. The neurobiology of apathy in depression and neurocognitive impairment in older adults: a review of epidemiological, clinical, neuropsychological and biological research. Transl Psychiatry. 2022;12:525. 10.1038/s41398-022-02292-3. 36572691 10.1038/s41398-022-02292-3 PMC9792580

[82] Andrade C. Methylphenidate and Other Pharmacologic Treatments for Apathy in Alzheimer’s Disease. J Clin Psychiatry 2022;83:22f14398. 10.4088/JCP.22f14398. 10.4088/JCP.22f14398 35120284

