# Supplementary material for: Exploring apathy components and their relationship in cognitive decline: insights from a network cross-sectional study
Source: BMC Psychol. 2025 Feb 17;13:129. doi: 10.1186/s40359-024-02239-x (PMC11834197; doi:10.1186/s40359-024-02239-x)
Supplement: Supplementary file 1 — Supplementary Material 1. [file 40359_2024_2239_MOESM1_ESM.pdf]

Apathy Evaluation Scale (Marin, 1991)

Somministrazione:

1. Le farò alcune domande riguardo i suoi pensieri, le sue emozioni e le sue attività. Risponda sulla base di ciò che ha pensato, sentito e fatto nelle ultime 4 settimane. Per iniziare, mi parli dei suoi attuali interessi. Mi parli di qualsiasi cosa sia di suo interesse. Per esempio, hobby o il lavoro; attività in cui è coinvolto o che le piace svolgere; interessi coltivati a casa o fuori casa, da solo o con altre persone; anche cose che in questo momento non è in grado di coltivare, ma che le interessano, ad esempio nuotare in mare, anche se è inverno (sciare anche se è estate) o leggere anche se la sua vista non glielo permette più bene come prima. (Riportare nelle note anche il numero degli interessi riportati; il grado di dettagli per ogni interesse; aspetti affettivi verbali e non verbali.)

2. Adesso vorrei che mi parlasse della sua giornata tipo, inizi da quando si sveglia e mi descriva la giornata e come passa il tempo finché non va a dormire. (Riportare nelle note anche il numero degli interessi riportati; il grado di dettagli per ogni interesse; aspetti affettivi verbali e non verbali.)

3. Proseguire con le domande della scala.

	**Per niente**		**Poco**		**Abbastanza**		**Molto**		
1. Si sente interessato alle cose?	4		3		2		1		+ C Q
2. Porta le cose a termine durante la giornata?	4		3		2		1		+ B Q
3. Sente che per lei è importante prendere l'iniziativa?	4		3		2		1		+ C SE
4. Si sente interessato a fare nuove esperienze?	4		3		2		1		+ C Q
5. Ha voglia di imparare cose nuove?	4		3		2		1		+ C Q
6. Sente che si impegna poco nel fare qualunque cosa?	1		2		3		4		- B
7. Sente di affrontare la vita con energia?	4		3		2		1		+ E
8. Sente che per lei è importante portare a termine un lavoro fino alla fine?	4		3		2		1		+ C SE
9. Trascorre il suo tempo facendo le cose che la interessano?	4		3		2		1		+ B
10. Sente che è meglio se qualcuno le dica cosa fare ogni giorno?	1		2		3		4		- B
11. Sente di essere interessato ai suoi problemi meno di quanto dovrebbe?	1		2		3		4		- C
12. Sente il bisogno di avere degli amici?	4		3		2		1		+ B Q
13. Sente che per lei è importante ritrovarsi e stare con gli amici?	4		3		2		1		+ C SE
14. Quando succede qualcosa di positivo si sente eccitato?	4		3		2		1		+ E

15. Sente di riuscire a capire bene i suoi problemi?	4		3		2		1		+ O
16. Sente che per lei è importante fare delle cose durante il giorno?	4		3		2		1		+ C SE
17. Sente di avere iniziativa?	4		3		2		1		+ O
18. Si sente motivato?	4		3		2		1		+ O
**TOTALE**	**/72**								

Punteggio 18-72.

Note a seguito di consegna, punti 1 e 2:

....
....
....
....
....
....
....
....
....
....
....
....
....
....
....
....
....
....
....
....
....
....
....
....
....
....
....

C: Cognitive item
B: Behavioral item
E: Emotional item
O: Other item
SE: Self-evaluation item
Q: Quantifiable item
