# Supplementary material for: Exploring apathy components and their relationship in cognitive decline: insights from a network cross-sectional study
Source: BMC Psychol. 2025 Feb 17;13:129. doi: 10.1186/s40359-024-02239-x (PMC11834197; doi:10.1186/s40359-024-02239-x)
Supplement: Supplementary file 2 — Supplementary Material 2. [file 40359_2024_2239_MOESM2_ESM.pdf]

Apathy Evaluation Scale (Clinician Version)

Name: ________________________________ Date:____/____/____

Rater: _________________________________

Rate each item based on an interview of the subject. The interview should begin with a description of the subject=s interests, activities and daily routine. Base your ratings on both verbal and non-verbal information. Ratings should be based on the past 4 weeks. For each item ratings should be judged:

Not at All Characteristic	Slightly Characteristic	Somewhat Characteristic	A Lot Characteristic
1	2	3	4

__	1.	S/he is interested in things.	+ C Q*
__	2.	S/he gets things done during the day.	+ B Q
__	3.	Getting things started on his/her own is important to him/her.	+ C SE
__	4.	S/he is interested in having new experiences.	+ C Q
__	5.	S/he is interested in learning new things.	+ C Q
__	6.	S/he puts little effort into anything	- B
__	7.	S/he approaches life with intensity.	+ E
__	8.	Seeing a job through to the end is important to her/him.	+ C SE
__	9.	S/he spends time doing things that interest her/him.	+ B
___	10.	Someone has to tell her/him what to do each day.	- B
__	11.	S/he is less concerned about her/his problems than s/he should be	- C
__	12.	S/he has friends.	+ B Q
__	13.	Getting together with friends is important to him/her.	+ C SE
__	14.	When something good happens, s/he gets excited.	+ E
__	15.	S/he has an accurate understanding of her/his problems.	+ O
__	16.	Getting things done during the day is important to her/him.	+ C SE
__	17.	S/he has initiative.	+ O
__	18.	S/he has motivation.	+ O

The Apathy Evaluation scale was developed by Robert S. Marin, M.D. Development and validation studies are described in Marin, R.S., Biedrzycki, RC., Firinciogullari, S. AReliability and Validity of the Apathy Evaluation Scale,@ Psychiatry Research, 38:143-162. 1991

*Note: Items that have positive versus negative syntax are identified by +/-. Type of item: C = cognitive; B = behavior; E = emotional; O = other. The definitions of self-evaluation (SE) items and quantifiable items (Q) are discussed in the administrations guidelines (3).
