# Supplementary material for: Exploring apathy components and their relationship in cognitive decline: insights from a network cross-sectional study
Source: BMC Psychol. 2025 Feb 17;13:129. doi: 10.1186/s40359-024-02239-x (PMC11834197; doi:10.1186/s40359-024-02239-x)

Rcode of Statistical analysis - Exploring apathy components and their relationship in cognitive decline: insights from a network analysis study -

Pierfrancesco Sarti and Sophie Tascedda

```r
# Uploading the dataset
# load(file = "mark_statistics.RData")

library(readxl)
data <- read_excel("data_all.xlsx")

# Exploring the dataset and checking for NAs
str(data)
```

```
## tibble [214 x 30] (S3: tbl_df/tbl/data.frame)
##  $ Age           : num [1:214] 80 67 75 88 71 70 77 78 83 66 ...
##  $ Sex           : num [1:214] 0 1 0 1 0 0 0 0 0 0 ...
##  $ Education     : num [1:214] 8 4 5 18 15 13 5 13 8 8 ...
##  $ Diagnosis     : num [1:214] 1 1 1 1 1 1 1 1 1 1 ...
##  $ Comorbidity   : num [1:214] 0 1 1 1 0 0 0 0 0 0 ...
##  $ Treatment     : num [1:214] 0 1 0 0 0 0 0 1 1 1 ...
##  $ Rehabilitation: num [1:214] 1 1 1 0 0 0 0 0 1 1 ...
##  $ MMSE          : num [1:214] 20 26 21 24 26 27 18 23 22 20 ...
##  $ Moca          : num [1:214] 14 25 14 15 20 15 9 13 11 12 ...
##  $ FAB           : num [1:214] 8 17 12 13 14 13 6 11 10 4 ...
##  $ Hamilton      : num [1:214] 3 14 11 17 0 14 2 23 17 23 ...
##  $ AES_TOT       : num [1:214] 29 51 36 29 22 38 29 37 37 39 ...
##  $ Item 1        : num [1:214] 1 3 2 2 2 3 2 1 2 3 ...
##  $ Item 2        : num [1:214] 1 2 2 1 1 2 1 3 2 3 ...
##  $ Item 3        : num [1:214] 1 4 2 1 1 2 2 1 2 3 ...
##  $ Item 4        : num [1:214] 2 4 4 3 2 4 3 1 2 3 ...
##  $ Item 5        : num [1:214] 3 3 3 2 2 4 3 4 2 1 ...
##  $ Item 6        : num [1:214] 1 4 1 1 1 3 2 2 2 2 ...
##  $ Item 7        : num [1:214] 2 3 2 2 2 2 2 4 2 2 ...
##  $ Item 8        : num [1:214] 1 2 2 1 1 2 1 2 2 2 ...
##  $ Item 9        : num [1:214] 1 3 2 2 1 1 1 2 2 2 ...
##  $ Item 10       : num [1:214] 1 3 2 1 1 1 1 3 2 2 ...
##  $ Item 11       : num [1:214] 3 2 1 3 1 2 1 3 1 1 ...
##  $ Item 12       : num [1:214] 4 1 4 2 1 3 1 1 2 3 ...
##  $ Item 13       : num [1:214] 3 2 3 1 1 1 1 1 2 3 ...
##  $ Item 14       : num [1:214] 1 4 1 1 1 1 1 1 2 3 ...
##  $ Item 15       : num [1:214] 1 3 1 1 1 1 2 3 3 2 ...
##  $ Item 16       : num [1:214] 1 1 1 2 1 1 1 1 2 1 ...
##  $ Item 17       : num [1:214] 1 4 1 2 1 3 2 2 3 1 ...
##  $ Item 18       : num [1:214] 1 3 2 1 1 2 2 2 2 2 ...
```

```r
summary(data)
```

```
##       Age             Sex           Education        Diagnosis     
##  Min.   :57.00   Min.   :0.0000   Min.   : 0.000   Min.   :0.0000  
##  1st Qu.:70.00   1st Qu.:0.0000   1st Qu.: 5.000   1st Qu.:0.0000  
##  Median :74.00   Median :0.0000   Median : 8.000   Median :0.5000  
##  Mean   :74.91   Mean   :0.2944   Mean   : 9.187   Mean   :0.6402  
##  3rd Qu.:80.00   3rd Qu.:1.0000   3rd Qu.:13.000   3rd Qu.:1.0000  
##  Max.   :92.00   Max.   :1.0000   Max.   :18.000   Max.   :2.0000  
##   Comorbidity       Treatment      Rehabilitation        MMSE      
##  Min.   :0.0000   Min.   :0.0000   Min.   :0.0000   Min.   :16.00  
##  1st Qu.:0.0000   1st Qu.:0.0000   1st Qu.:0.0000   1st Qu.:24.25  
```

```
##  Median :0.0000   Median :0.0000   Median :0.0000   Median :28.00
##  Mean   :0.1308   Mean   :0.1215   Mean   :0.3131   Mean   :26.73
##  3rd Qu.:0.0000   3rd Qu.:0.0000   3rd Qu.:1.0000   3rd Qu.:30.00
##  Max.   :1.0000   Max.   :1.0000   Max.   :1.0000   Max.   :30.00
##       Moca            FAB            Hamilton         AES_TOT
##  Min.   : 4.00   Min.   : 3.00   Min.   : 0.000   Min.   :12.00
##  1st Qu.:15.00   1st Qu.:10.25   1st Qu.: 2.000   1st Qu.:24.00
##  Median :25.00   Median :13.00   Median : 6.000   Median :32.00
##  Mean   :21.09   Mean   :12.89   Mean   : 7.332   Mean   :32.96
##  3rd Qu.:27.00   3rd Qu.:16.00   3rd Qu.:11.000   3rd Qu.:39.00
##  Max.   :30.00   Max.   :18.00   Max.   :38.000   Max.   :67.00
##      Item 1          Item 2          Item 3          Item 4
##  Min.   :1.000   Min.   :1.000   Min.   :1.000   Min.   :1.000
##  1st Qu.:1.000   1st Qu.:1.000   1st Qu.:1.000   1st Qu.:1.000
##  Median :2.000   Median :1.000   Median :2.000   Median :2.000
##  Mean   :1.897   Mean   :1.659   Mean   :1.916   Mean   :2.453
##  3rd Qu.:2.000   3rd Qu.:2.000   3rd Qu.:3.000   3rd Qu.:3.000
##  Max.   :4.000   Max.   :4.000   Max.   :4.000   Max.   :4.000
##      Item 5          Item 6         Item 7      Item 8          Item 9
##  Min.   :1.000   Min.   :1.00   Min.   :1   Min.   :1.000   Min.   :1.000
##  1st Qu.:1.000   1st Qu.:1.00   1st Qu.:1   1st Qu.:1.000   1st Qu.:1.000
##  Median :2.000   Median :2.00   Median :2   Median :1.000   Median :2.000
##  Mean   :2.393   Mean   :1.85   Mean   :2   Mean   :1.449   Mean   :1.841
##  3rd Qu.:3.000   3rd Qu.:2.75   3rd Qu.:3   3rd Qu.:2.000   3rd Qu.:2.000
##  Max.   :4.000   Max.   :4.00   Max.   :4   Max.   :4.000   Max.   :4.000
##     Item 10        Item 11          Item 12          Item 13          Item 14
##  Min.   :1.00   Min.   :1.000   Min.   :1.000   Min.   :1.000   Min.   :1.000
##  1st Qu.:1.00   1st Qu.:1.000   1st Qu.:1.000   1st Qu.:1.000   1st Qu.:1.000
##  Median :1.00   Median :2.000   Median :1.500   Median :1.000   Median :1.000
##  Mean   :1.71   Mean   :1.986   Mean   :1.785   Mean   :1.621   Mean   :1.435
##  3rd Qu.:2.00   3rd Qu.:3.000   3rd Qu.:2.000   3rd Qu.:2.000   3rd Qu.:2.000
##  Max.   :4.00   Max.   :4.000   Max.   :4.000   Max.   :4.000   Max.   :4.000
##     Item 15        Item 16          Item 17          Item 18
##  Min.   :1.00   Min.   :1.000   Min.   :1.000   Min.   :1.000
##  1st Qu.:1.00   1st Qu.:1.000   1st Qu.:1.000   1st Qu.:1.000
##  Median :2.00   Median :1.000   Median :2.000   Median :2.000
##  Mean   :1.72   Mean   :1.528   Mean   :1.916   Mean   :1.949
##  3rd Qu.:2.00   3rd Qu.:2.000   3rd Qu.:3.000   3rd Qu.:3.000
##  Max.   :4.00   Max.   :4.000   Max.   :4.000   Max.   :4.000
```

```
head(data)
```

```
## # A tibble: 6 x 30
##     Age   Sex Education Diagnosis Comorbidity Treatment Rehabilitation  MMSE
##   <dbl> <dbl>     <dbl>     <dbl>       <dbl>     <dbl>          <dbl> <dbl>
## 1    80     0         8         1           0         0              1    20
## 2    67     1         4         1           1         1              1    26
## 3    75     0         5         1           1         0              1    21
## 4    88     1        18         1           1         0              0    24
## 5    71     0        15         1           0         0              0    26
## 6    70     0        13         1           0         0              0    27
## # i 22 more variables: Moca <dbl>, FAB <dbl>, Hamilton <dbl>, AES_TOT <dbl>,
## #   ‘Item 1‘ <dbl>, ‘Item 2‘ <dbl>, ‘Item 3‘ <dbl>, ‘Item 4‘ <dbl>,
## #   ‘Item 5‘ <dbl>, ‘Item 6‘ <dbl>, ‘Item 7‘ <dbl>, ‘Item 8‘ <dbl>,
```

```
## #   ‘Item 9‘ <dbl>, ‘Item 10‘ <dbl>, ‘Item 11‘ <dbl>, ‘Item 12‘ <dbl>,
## #   ‘Item 13‘ <dbl>, ‘Item 14‘ <dbl>, ‘Item 15‘ <dbl>, ‘Item 16‘ <dbl>,
## #   ‘Item 17‘ <dbl>, ‘Item 18‘ <dbl>
```

```
sum(is.na(data)) # There are no NAs in the dataset
```

```
## [1] 0
```

```
# Subsetting the dataframe based on the diagnosis

# data$Sex <- factor(data$Sex, levels = c(0,1),
#                                      labels = c("Female", "Male"))
# data$Diagnosis <- factor(data$Diagnosis, levels = c(0,1,2),
#                                     labels = c("Control", "MCI", "Alzheimer"))
# data$Comorbidity <- factor(data$Comorbidity, levels = c(0,1),
#                                 labels = c("Absence of Comorbidity",
#                                            "Presence of Comorbidity"))
# data$Treatment <- factor(data$Treatment, levels = c(0,1),
#                                labels = c("No treatment", "Under treatment"))
# data$Rehabilitation <- factor(data$Rehabilitation, levels = c(0,1),
#                               labels = c("No rehab", "Under rehab"))



control_data <- subset(data, data$Diagnosis == 0)
mci_data <- subset(data, data$Diagnosis == 1)
alzheimer_data <- subset(data, data$Diagnosis == 2)
```

```
# Representations of subjects; MoCA/Hamilton and AES_TOT devided by DIAGNOSIS
library(ggplot2)

data$Diagnosis <- factor(data$Diagnosis, levels = c(0,1,2),
                         labels = c("Control", "MCI", "Alzheimer"))
colnames(data)[9] <- "MoCA"

ggplot(data = data, aes(MoCA, AES_TOT, fill = Diagnosis)) +
  geom_point(size = 4, shape = 21) +
  geom_smooth(aes(color = Diagnosis, fill = Diagnosis), method = "lm") +
  scale_color_manual(values=c("#fde725","#21918c", "#440154")) +
  scale_fill_manual(values=c("#fde725","#21918c",  "#440154")) +
  scale_x_continuous(breaks = seq(0,30,5)) +
  scale_y_continuous(breaks = seq(0,70,5)) +
  xlab("Montreal Cognitive Assessment - Score") +
  ylab("Apathy Evaluation Scale - Score") +
  ggtitle("MoCA * AES") +
  theme(axis.text = element_text(size = 14),
        axis.title = element_text(size = 16),
        plot.title = element_text(size = 20, face = "bold"))
```

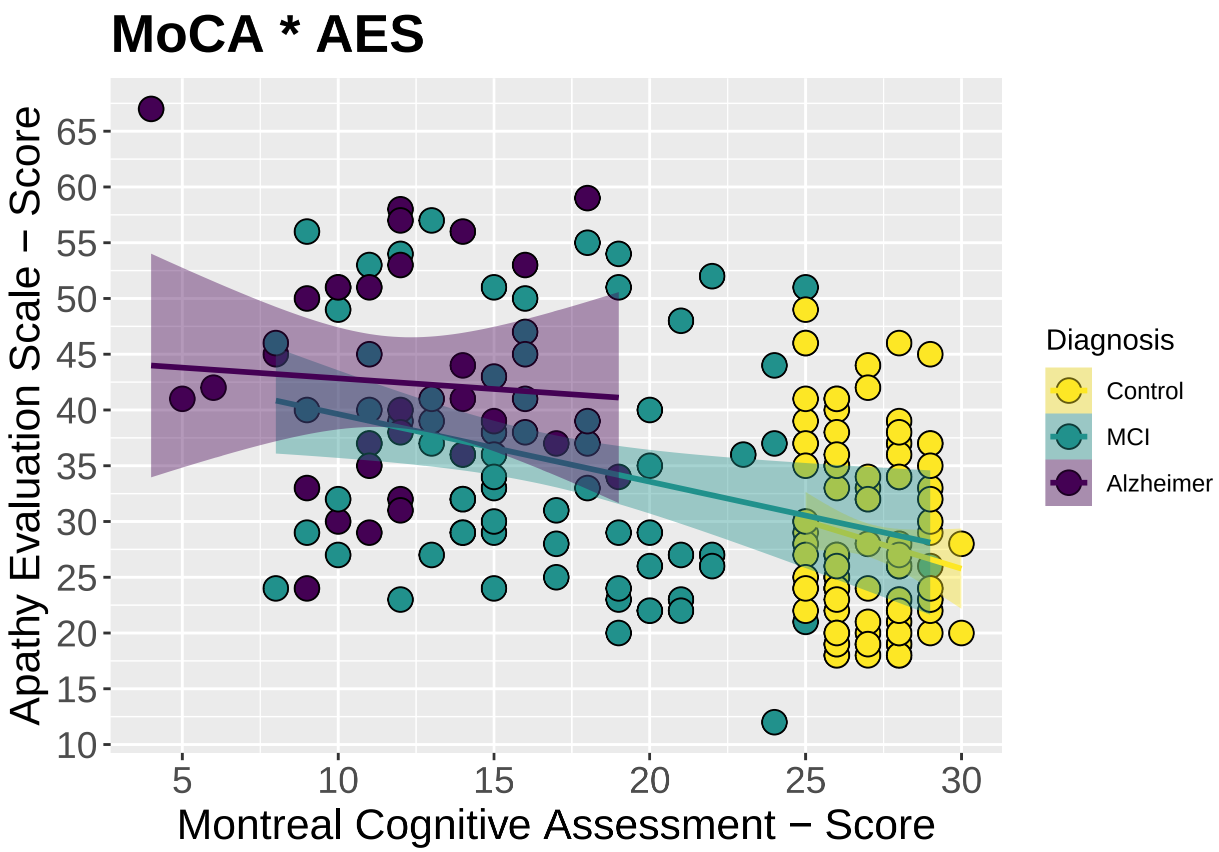


```r
ggplot(data = data, aes(MoCA, Hamilton, fill = Diagnosis)) +
  geom_point(size = 4, shape = 21) +
  geom_smooth(aes(color = Diagnosis, fill = Diagnosis), method = "lm") +
  scale_color_manual(values=c("#fde725","#21918c", "#440154")) +
  scale_fill_manual(values=c("#fde725","#21918c",  "#440154")) +
  scale_x_continuous(breaks = seq(0,30,5)) +
  scale_y_continuous(breaks = seq(0,40,5)) +
  xlab("Montreal Cognitive Assessment - Score") +
  ylab("Hamilton Depression Rating Scale - Score") +
  ggtitle("MoCA * HAMILTON") +
  theme(axis.text = element_text(size = 14),
        axis.title = element_text(size = 16),
        plot.title = element_text(size = 20, face = "bold"))
```

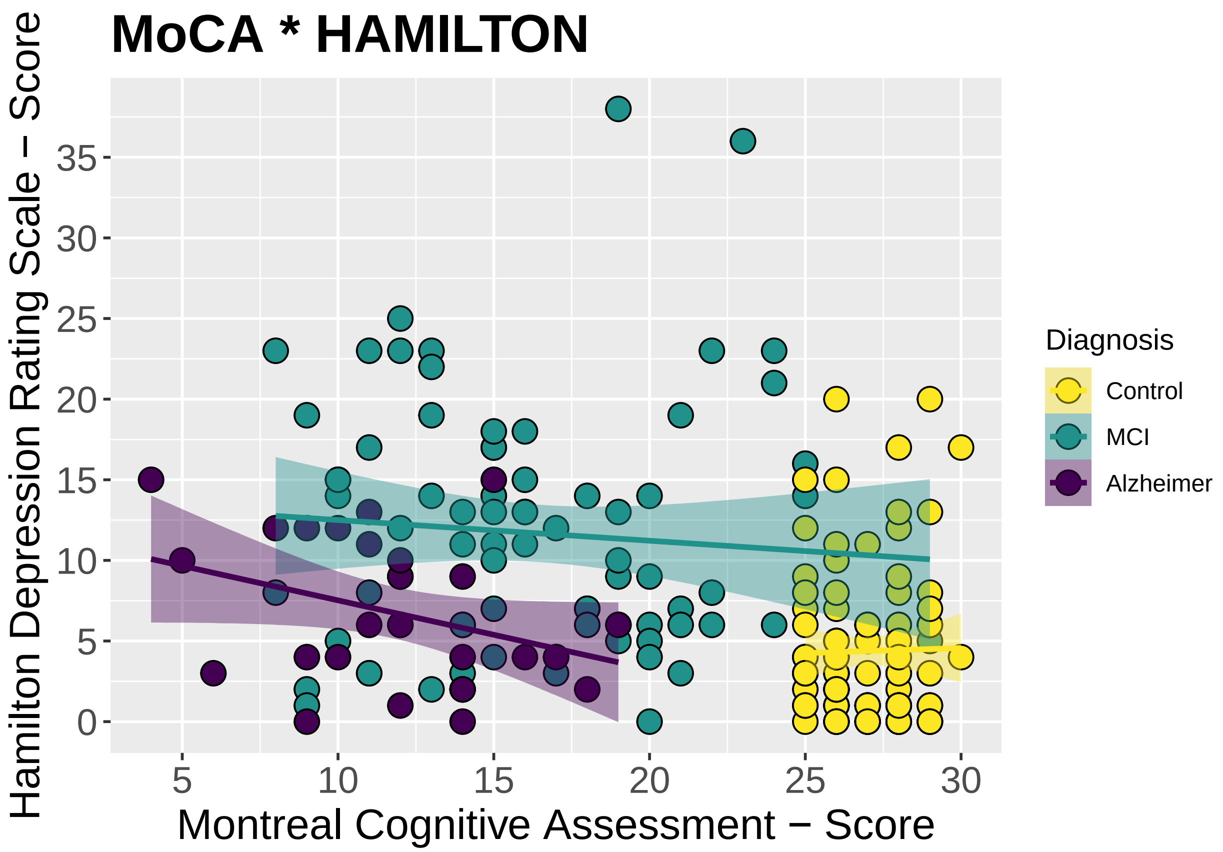


```
# Correlation in all the sample and relative correlation plot
library(corrplot)
data <- read_excel("data_all.xlsx")

matrix_data <- as.matrix(data)
matrix_data <- matrix_data[,-c(13:30)]
corr_all <- cor(matrix_data, method = "spearman")
corr_all
```

```
##                        Age          Sex    Education   Diagnosis Comorbidity
## Age             1.00000000  0.042793830 -0.238834131  0.17114147  0.12488261
## Sex             0.04279383  1.000000000  0.029111377  0.06319652  0.02301552
## Education      -0.23883413  0.029111377  1.000000000 -0.20104069 -0.13034363
## Diagnosis       0.17114147  0.063196518 -0.201040689  1.00000000  0.38496894
## Comorbidity     0.12488261  0.023015519 -0.130343629  0.38496894  1.00000000
## Treatment      -0.02237301 -0.051913333 -0.004037464  0.25506113  0.27985535
## Rehabilitation  0.05504229  0.050312297 -0.230891742  0.67431547  0.24602223
## MMSE           -0.31380118 -0.028354201  0.409153183 -0.79179822 -0.29813089
## Moca           -0.32080233 -0.002416027  0.423796929 -0.85628867 -0.34747453
## FAB            -0.28109920  0.025489933  0.529306680 -0.69780266 -0.25319783
## Hamilton        0.08258042 -0.095431539 -0.229637582  0.39052840  0.34667721
## AES_TOT         0.06459900 -0.012787746 -0.390456448  0.46917038  0.25643821
##                   Treatment Rehabilitation       MMSE         Moca         FAB
## Age            -0.022373009     0.05504229 -0.3138012 -0.320802335 -0.28109920
## Sex            -0.051913333     0.05031230 -0.0283542 -0.002416027  0.02548993
## Education      -0.004037464    -0.23089174  0.4091532  0.423796929  0.52930668
```

```
## Diagnosis       0.255061129     0.67431547 -0.7917982 -0.856288671 -0.69780266
## Comorbidity     0.279855348     0.24602223 -0.2981309 -0.347474534 -0.25319783
## Treatment       1.000000000     0.30410306 -0.2420440 -0.251858064 -0.18454017
## Rehabilitation  0.304103058     1.00000000 -0.6058443 -0.623172386 -0.56717596
## MMSE           -0.242044042    -0.60584432  1.0000000  0.875196815  0.79242655
## Moca           -0.251858064    -0.62317239  0.8751968  1.000000000  0.85846064
## FAB            -0.184540166    -0.56717596  0.7924265  0.858460643  1.00000000
## Hamilton        0.304684924     0.38815968 -0.3802591 -0.410970632 -0.41772811
## AES_TOT         0.157313574     0.44835768 -0.4484445 -0.488596447 -0.50863223
##                   Hamilton     AES_TOT
## Age             0.08258042  0.06459900
## Sex            -0.09543154 -0.01278775
## Education      -0.22963758 -0.39045645
## Diagnosis       0.39052840  0.46917038
## Comorbidity     0.34667721  0.25643821
## Treatment       0.30468492  0.15731357
## Rehabilitation  0.38815968  0.44835768
## MMSE           -0.38025907 -0.44844454
## Moca           -0.41097063 -0.48859645
## FAB            -0.41772811 -0.50863223
## Hamilton        1.00000000  0.55928798
## AES_TOT         0.55928798  1.00000000
```

```
testRes_all = cor.mtest(matrix_data, conf.level = 0.95)

corrplot(corr_all, p.mat = testRes_all$p, method = 'shade', type = 'lower',
         insig='blank', addgrid.col = "grey",
         addCoef.col ='black', number.cex=7/ncol(matrix_data),
         order = 'original', mar = c(0,0,3,0),
         diag=FALSE, title = "Correlation matrix of the entire sample")
```

Correlation matrix of the entire sample

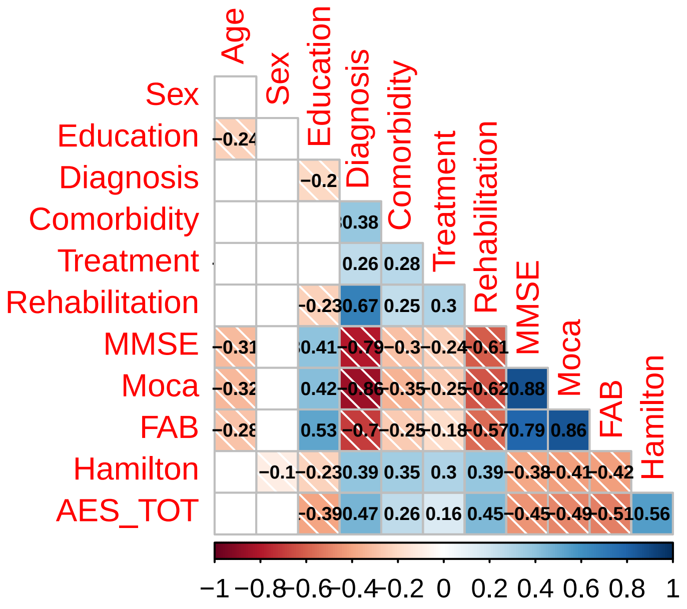


```r
# pdf("Correlation_All")
# dev.off()

# CORRELATION MATRICES devided for each group
matrix_c <- as.matrix(control_data)
matrix_m <- as.matrix(mci_data)
matrix_a <- as.matrix(alzheimer_data)

matrix_c <- matrix_c[,-c(4:7,13:30)]
matrix_m <- matrix_m[,-c(4,13:30)]
matrix_a <- matrix_a[,-c(4,6,13:30)]

# Correlation Controls

corr_c <- cor(matrix_c, method = "spearman")
corr_c
```

```
##                    Age          Sex    Education        MMSE         Moca
## Age        1.000000000  0.097871256 -0.331493347 -0.45933149 -0.510831988
## Sex        0.097871256  1.000000000  0.003143483 -0.06147292  0.002447881
## Education -0.331493347  0.003143483  1.000000000  0.56690295  0.633892814
## MMSE      -0.459331491 -0.061472917  0.566902949  1.00000000  0.554012037
## Moca      -0.510831988  0.002447881  0.633892814  0.55401204  1.000000000
## FAB       -0.397907726 -0.054850100  0.667528827  0.51265664  0.723279200
## Hamilton  -0.055725557 -0.042185389 -0.248649924 -0.24137640 -0.067740082
```

```
## AES_TOT   -0.005700163 -0.007838717 -0.460952337 -0.17224083 -0.154109116
##                  FAB    Hamilton      AES_TOT
## Age       -0.3979077 -0.05572556 -0.005700163
## Sex       -0.0548501 -0.04218539 -0.007838717
## Education  0.6675288 -0.24864992 -0.460952337
## MMSE       0.5126566 -0.24137640 -0.172240833
## Moca       0.7232792 -0.06774008 -0.154109116
## FAB        1.0000000 -0.17956018 -0.330747253
## Hamilton  -0.1795602  1.00000000  0.576028344
## AES_TOT   -0.3307473  0.57602834  1.000000000
```

```r
testRes_c = cor.mtest(matrix_c, conf.level = 0.95)

corrplot(corr_c, p.mat = testRes_c$p, method = 'shade', type = 'lower',
         insig='blank', addgrid.col = "grey",
         addCoef.col ='black', number.cex=7/ncol(matrix_c),
         order = 'original', mar = c(0,0,3,0),
         diag=FALSE, title = "Correlations matrix - Control group")
```

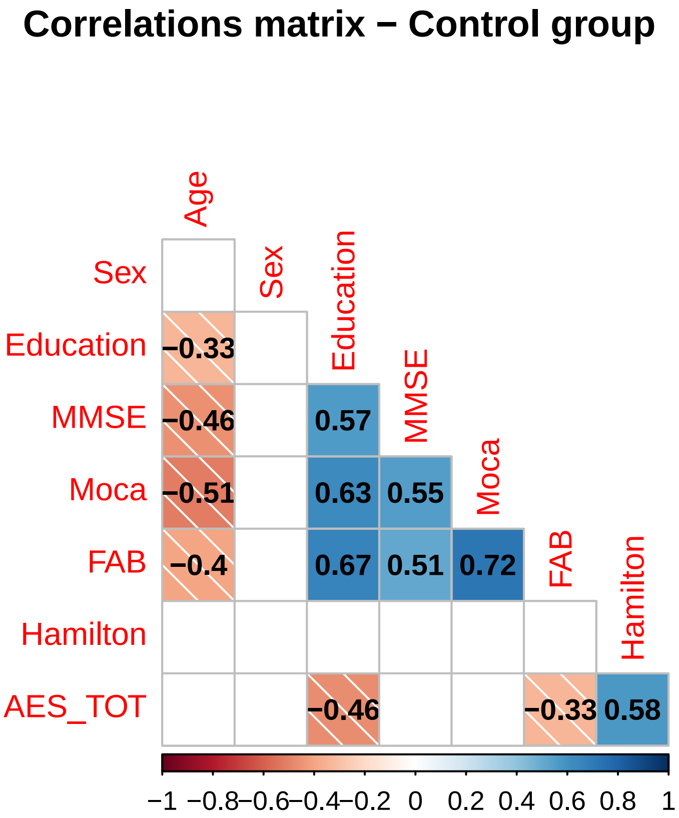


```r
# Correlation MCI
corr_m <- cor(matrix_m, method = "spearman")
corr_m
```

```
##                           Age         Sex   Education Comorbidity   Treatment
## Age                1.00000000 -0.03899537 -0.04648987  0.04954229 -0.12868726
```

```
## Sex           -0.03899537  1.00000000  0.06387665 -0.04444795 -0.10597571
## Education     -0.04648987  0.06387665  1.00000000 -0.24158336  0.05409277
## Comorbidity    0.04954229 -0.04444795 -0.24158336  1.00000000  0.22832135
## Treatment     -0.12868726 -0.10597571  0.05409277  0.22832135  1.00000000
## Rehabilitation -0.11161449 -0.08300054 -0.37571112  0.05477433  0.15631503
## MMSE          -0.09131337  0.17952369  0.31932996  0.14293975 -0.03474888
## Moca          -0.12577701  0.22908771  0.42204287  0.06453753  0.08360679
## FAB            0.01125911  0.32699825  0.53500162  0.05649673  0.03971406
## Hamilton       0.08428667 -0.18224554 -0.23769305  0.39102501  0.15471809
## AES_TOT       -0.10529889 -0.17698286 -0.25241725  0.14515607  0.09708231
##               Rehabilitation        MMSE        Moca         FAB    Hamilton
## Age              -0.11161449 -0.09131337 -0.12577701  0.01125911  0.08428667
## Sex              -0.08300054  0.17952369  0.22908771  0.32699825 -0.18224554
## Education        -0.37571112  0.31932996  0.42204287  0.53500162 -0.23769305
## Comorbidity       0.05477433  0.14293975  0.06453753  0.05649673  0.39102501
## Treatment         0.15631503 -0.03474888  0.08360679  0.03971406  0.15471809
## Rehabilitation    1.00000000 -0.21972003 -0.21988724 -0.31857272  0.31475125
## MMSE             -0.21972003  1.00000000  0.72836784  0.59629998  0.02044778
## Moca             -0.21988724  0.72836784  1.00000000  0.67081081 -0.13558274
## FAB              -0.31857272  0.59629998  0.67081081  1.00000000 -0.20936383
## Hamilton          0.31475125  0.02044778 -0.13558274 -0.20936383  1.00000000
## AES_TOT           0.34059861 -0.19588994 -0.29402218 -0.33915907  0.49557160
##                   AES_TOT
## Age           -0.10529889
## Sex           -0.17698286
## Education     -0.25241725
## Comorbidity    0.14515607
## Treatment      0.09708231
## Rehabilitation  0.34059861
## MMSE          -0.19588994
## Moca          -0.29402218
## FAB           -0.33915907
## Hamilton       0.49557160
## AES_TOT        1.00000000
```

```r
testRes_m = cor.mtest(matrix_m, conf.level = 0.95)

corrplot(corr_m, p.mat = testRes_m$p, method = 'shade', type = 'lower',
         insig='blank', addgrid.col = "grey",
         addCoef.col ='black', number.cex=7/ncol(matrix_m),
         order = 'original', mar = c(0,0,3,0),
         diag=FALSE, title = " Correlation matrix - MCI group")
```

Correlation matrix − MCI group

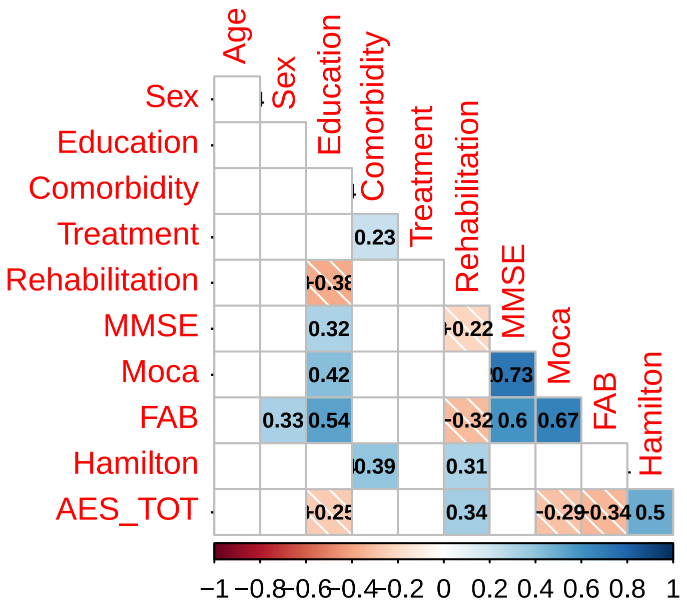


```r
# Correlation Alzheimer
corr_a <- cor(matrix_a, method = "spearman")
corr_a
```

```
##                       Age         Sex   Education Comorbidity Rehabilitation
## Age             1.0000000 -0.06814940 -0.10799912  0.15597575     -0.1528967
## Sex            -0.0681494  1.00000000  0.11854654  0.10566113      0.3024116
## Education      -0.1079991  0.11854654  1.00000000  0.17194642      0.1202739
## Comorbidity     0.1559758  0.10566113  0.17194642  1.00000000     -0.2631807
## Rehabilitation -0.1528967  0.30241165  0.12027392 -0.26318068      1.0000000
## MMSE           -0.3403421  0.00000000  0.34143501 -0.37666378      0.2105134
## Moca           -0.2284160  0.16147631  0.37409535 -0.30989455      0.3299339
## FAB            -0.2178213  0.06429195  0.40417852 -0.11408869      0.2276967
## Hamilton        0.1268385 -0.29741718 -0.09580225  0.16905833     -0.2233682
## AES_TOT         0.2502242  0.19990300 -0.01787578  0.05885991      0.1481307
##                      MMSE        Moca         FAB    Hamilton      AES_TOT
## Age            -0.34034207 -0.22841595 -0.21782128  0.12683845  0.25022421
## Sex             0.00000000  0.16147631  0.06429195 -0.29741718  0.19990300
## Education       0.34143501  0.37409535  0.40417852 -0.09580225 -0.01787578
## Comorbidity    -0.37666378 -0.30989455 -0.11408869  0.16905833  0.05885991
## Rehabilitation  0.21051336  0.32993387  0.22769671 -0.22336818  0.14813069
## MMSE            1.00000000  0.50718969  0.31816173 -0.16508918 -0.08810864
## Moca            0.50718969  1.00000000  0.78246969 -0.31445993  0.03879201
## FAB             0.31816173  0.78246969  1.00000000 -0.36599911 -0.03559083
## Hamilton       -0.16508918 -0.31445993 -0.36599911  1.00000000  0.12347647
## AES_TOT        -0.08810864  0.03879201 -0.03559083  0.12347647  1.00000000
```

```
testRes_a = cor.mtest(matrix_a, conf.level = 0.95)

?corrplot
corrplot(corr_a,  p.mat = testRes_a$p, method = 'shade', type = 'lower',
         insig='blank', addgrid.col = "grey",
         addCoef.col ='black',number.cex=7/ncol(matrix_m),
         order = 'original', mar = c(0,0,3,0),
         diag=FALSE, title = "Correlation matrix - Alzheimer group")
```

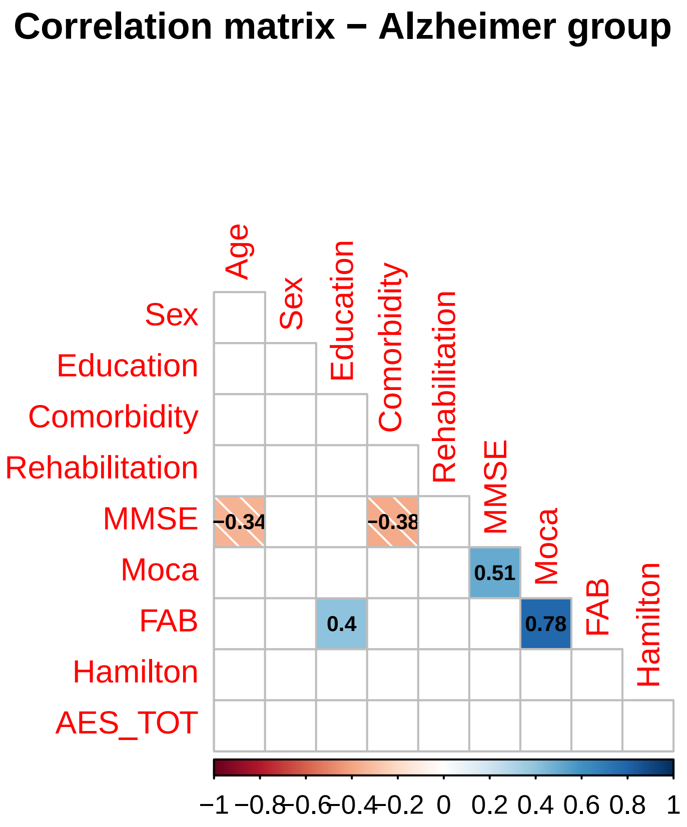


```
# PDF WITH CORRELATIONS PLOTS
# pdf("Correlations", width = 20)
# par(mfrow = c(1,3))
# corrplot(corr_c, p.mat = testRes_c$p, method = 'shade', type = 'lower',
#          insig='blank', addgrid.col = "grey",
#          addCoef.col ='black', number.cex=7/ncol(matrix_c),
#          order = 'original', mar = c(0,0,3,0),
#          diag=FALSE, title = "Correlations Control group")
# corrplot(corr_m, p.mat = testRes_m$p, method = 'shade', type = 'lower',
#          insig='blank', addgrid.col = "grey",
#          addCoef.col ='black', number.cex=7/ncol(matrix_m),
#          order = 'original', mar = c(0,0,3,0),
#          diag=FALSE, title = " Correlations MCI group")
# ccorrplot(corr_a,  p.mat = testRes_a$p, method = 'shade', type = 'lower',
#          insig='blank', addgrid.col = "grey",
#          addCoef.col ='black',number.cex=7/ncol(matrix_m),
```

```r
#           order = 'original', mar = c(0,0,3,0),
#           diag=FALSE, title = "Correlations Alzheimer group")
# dev.off()
```

```r
# REGRESSION MODEL - USE AKAIKE TO SELECT BEST FITTING MODEL; FROM NULL TO FULL
library(MASS)
par(mfrow = c(2,2))

# All the dataset
data1 <- data[,-c(13:30)]
null_all = lm(AES_TOT  ~ 1., data = data1)
full_all = lm(AES_TOT  ~ ., data = data1)

stepAIC(null_all, scope = list(lower = null_all, upper = full_all),
     direction = "both")
```

```
## Start:  AIC=1010.74
## AES_TOT ~ 1
##
##                  Df Sum of Sq   RSS     AIC
## + Moca            1    5990.8 17863  950.84
## + FAB             1    5888.4 17965  952.07
## + Hamilton        1    5804.1 18050  953.07
## + Diagnosis       1    5568.4 18285  955.85
## + MMSE            1    4922.9 18931  963.27
## + Rehabilitation  1    4810.0 19044  964.54
## + Education       1    3105.7 20748  982.89
## + Comorbidity     1    1710.8 22143  996.81
## + Treatment       1     529.5 23324 1007.93
## <none>                        23854 1010.74
## + Age             1      15.5 23838 1012.60
## + Sex             1       0.0 23854 1012.74
##
## Step:  AIC=950.84
## AES_TOT ~ Moca
##
##                  Df Sum of Sq   RSS     AIC
## + Hamilton        1    2696.3 15167  917.83
## + Education       1     824.0 17039  942.74
## + Rehabilitation  1     685.9 17177  944.47
## + FAB             1     447.7 17415  947.41
## + Diagnosis       1     247.2 17616  949.86
## + Comorbidity     1     241.4 17622  949.93
## + Age             1     186.1 17677  950.60
## <none>                        17863  950.84
## + Treatment       1      45.7 17817  952.30
## + MMSE            1      30.3 17833  952.48
## + Sex             1       0.5 17862  952.84
## - Moca            1    5990.8 23854 1010.74
##
## Step:  AIC=917.83
## AES_TOT ~ Moca + Hamilton
##
```

```
##                  Df Sum of Sq   RSS    AIC
## + Education       1    659.75 14507 910.31
## + Diagnosis       1    317.00 14850 915.31
## + Rehabilitation  1    276.91 14890 915.89
## + FAB             1    192.35 14974 917.10
## <none>                        15167 917.83
## + MMSE            1    139.81 15027 917.85
## + Age             1    128.10 15039 918.01
## + Sex             1     73.83 15093 918.78
## + Treatment       1     56.33 15110 919.03
## + Comorbidity     1      1.93 15165 919.80
## - Hamilton        1   2696.26 17863 950.84
## - Moca            1   2882.95 18050 953.07
##
## Step:  AIC=910.31
## AES_TOT ~ Moca + Hamilton + Education
##
##                  Df Sum of Sq   RSS    AIC
## + Diagnosis       1    530.89 13976 904.33
## + Rehabilitation  1    273.78 14233 908.23
## + Age             1    207.19 14300 909.23
## <none>                        14507 910.31
## + MMSE            1    101.33 14406 910.81
## + Sex             1     81.64 14425 911.10
## + Treatment       1     30.67 14476 911.86
## + FAB             1     10.58 14496 912.15
## + Comorbidity     1      3.02 14504 912.27
## - Education       1    659.75 15167 917.83
## - Moca            1   1752.25 16259 932.71
## - Hamilton        1   2532.04 17039 942.74
##
## Step:  AIC=904.33
## AES_TOT ~ Moca + Hamilton + Education + Diagnosis
##
##                  Df Sum of Sq   RSS    AIC
## - Moca            1      7.58 13984 902.45
## + Age             1    138.35 13838 904.20
## <none>                        13976 904.33
## + Rehabilitation  1    109.37 13867 904.65
## + MMSE            1     90.64 13885 904.94
## + FAB             1     36.26 13940 905.78
## + Sex             1     33.24 13943 905.82
## + Treatment       1     32.43 13944 905.84
## + Comorbidity     1      4.76 13971 906.26
## - Diagnosis       1    530.89 14507 910.31
## - Education       1    873.64 14850 915.31
## - Hamilton        1   2598.00 16574 938.82
##
## Step:  AIC=902.45
## AES_TOT ~ Hamilton + Education + Diagnosis
##
##                  Df Sum of Sq   RSS    AIC
## <none>                        13984 902.45
## + Age             1    121.30 13862 902.58
```

```
## + Rehabilitation  1    114.77 13869 902.69
## + MMSE            1     82.58 13901 903.18
## + FAB             1     41.01 13943 903.82
## + Treatment       1     30.35 13953 903.98
## + Sex             1     29.91 13954 903.99
## + Moca            1      7.58 13976 904.33
## + Comorbidity     1      4.90 13979 904.37
## - Education       1   1047.05 15031 915.90
## - Diagnosis       1   2275.56 16259 932.71
## - Hamilton        1   2754.92 16739 938.93
```

```
##
## Call:
## lm(formula = AES_TOT ~ Hamilton + Education + Diagnosis, data = data1)
##
## Coefficients:
## (Intercept)     Hamilton    Education    Diagnosis
##     29.7925       0.5576      -0.4384       4.8569
```

```
fit_all <- lm(AES_TOT ~ Hamilton + Education + Diagnosis, data = data1)
summary(fit_all)
```

```
##
## Call:
## lm(formula = AES_TOT ~ Hamilton + Education + Diagnosis, data = data1)
##
## Residuals:
##     Min      1Q  Median      3Q     Max
## -33.283  -5.185  -1.428   5.695  25.831
##
## Coefficients:
##             Estimate Std. Error t value Pr(>|t|)
## (Intercept) 29.79255    1.49241  19.963  < 2e-16 ***
## Hamilton     0.55763    0.08669   6.432 8.36e-10 ***
## Education   -0.43841    0.11056  -3.965    1e-04 ***
## Diagnosis    4.85694    0.83084   5.846 1.91e-08 ***
## ---
## Signif. codes:  0 '***' 0.001 '**' 0.01 '*' 0.05 '.' 0.1 ' ' 1
##
## Residual standard error: 8.16 on 210 degrees of freedom
## Multiple R-squared:  0.4138, Adjusted R-squared:  0.4054
## F-statistic: 49.41 on 3 and 210 DF,  p-value: < 2.2e-16
```

```
plot(fit_all)
```

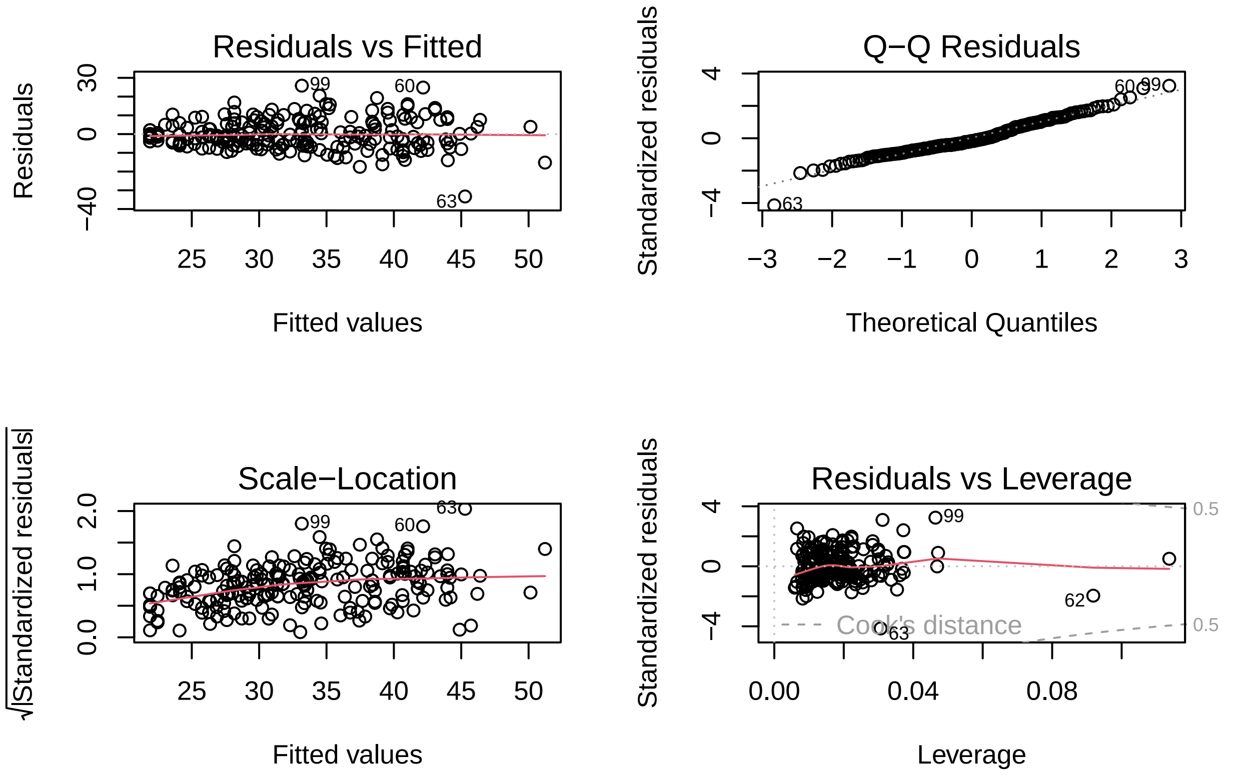


```
# Controls
control_data1 <- control_data[, -c(4:7,13:30)]

null_c = lm(AES_TOT ~ 1., data = control_data1)
full_c = lm(AES_TOT ~ ., data = control_data1)
step(null_c, scope = list(lower = null_c, upper = full_c),
     direction = "both")
```

```
## Start:  AIC=441.49
## AES_TOT ~ 1
##
##              Df Sum of Sq    RSS    AIC
## + Hamilton    1   2095.24 4409.0 401.89
## + Education   1   1361.83 5142.4 418.35
## + FAB         1    601.39 5902.8 433.11
## + Moca        1    147.01 6357.2 441.04
## <none>                    6504.2 441.49
## + MMSE        1    108.66 6395.5 441.69
## + Age         1      2.88 6501.3 443.44
## + Sex         1      0.77 6503.4 443.48
##
## Step:  AIC=401.89
## AES_TOT ~ Hamilton
##
##              Df Sum of Sq    RSS    AIC
## + Education   1    992.31 3416.7 376.60
```

```
## + FAB        1    421.54 3987.4 393.13
## + Moca       1    168.93 4240.0 399.71
## <none>                   4409.0 401.89
## + Sex        1     25.42 4383.5 403.27
## + MMSE       1     12.25 4396.7 403.59
## + Age        1      1.25 4407.7 403.86
## - Hamilton   1   2095.24 6504.2 441.49
##
## Step:  AIC=376.6
## AES_TOT ~ Hamilton + Education
##
##             Df Sum of Sq    RSS    AIC
## + MMSE       1    227.73 3188.9 371.22
## + Moca       1     88.37 3328.3 375.80
## + Age        1     84.24 3332.4 375.93
## <none>                   3416.7 376.60
## + Sex        1     19.11 3397.5 378.00
## + FAB        1      0.43 3416.2 378.59
## - Education  1    992.31 4409.0 401.89
## - Hamilton   1   1725.72 5142.4 418.35
##
## Step:  AIC=371.22
## AES_TOT ~ Hamilton + Education + MMSE
##
##             Df Sum of Sq    RSS    AIC
## <none>                   3188.9 371.22
## + Sex        1     24.99 3163.9 372.38
## + Moca       1     24.49 3164.4 372.40
## + Age        1     14.14 3174.8 372.75
## + FAB        1      7.36 3181.6 372.98
## - MMSE       1    227.73 3416.7 376.60
## - Education  1   1207.79 4396.7 403.59
## - Hamilton   1   1838.53 5027.5 417.93
```

```
##
## Call:
## lm(formula = AES_TOT ~ Hamilton + Education + MMSE, data = control_data1)
##
## Coefficients:
## (Intercept)     Hamilton    Education         MMSE
##    -29.4456       0.9209      -0.6939       2.0754
```

```r
fit_control <- lm(AES_TOT ~ Hamilton + Education + MMSE, data = control_data1)
summary(fit_control)
```

```
##
## Call:
## lm(formula = AES_TOT ~ Hamilton + Education + MMSE, data = control_data1)
##
## Residuals:
##      Min       1Q   Median       3Q      Max
## -12.3386  -3.8332  -0.3253   3.7038  14.7329
##
```

```
## Coefficients:
##             Estimate Std. Error t value Pr(>|t|)
## (Intercept) -29.4456    21.9531  -1.341  0.18277
## Hamilton      0.9209     0.1195   7.706 8.40e-12 ***
## Education    -0.6939     0.1111  -6.246 9.58e-09 ***
## MMSE          2.0754     0.7652   2.712  0.00784 **
## ---
## Signif. codes:  0 '***' 0.001 '**' 0.01 '*' 0.05 '.' 0.1 ' ' 1
##
## Residual standard error: 5.564 on 103 degrees of freedom
## Multiple R-squared:  0.5097, Adjusted R-squared:  0.4954
## F-statistic: 35.69 on 3 and 103 DF,  p-value: 6.719e-16
```

```r
plot(fit_control)
```

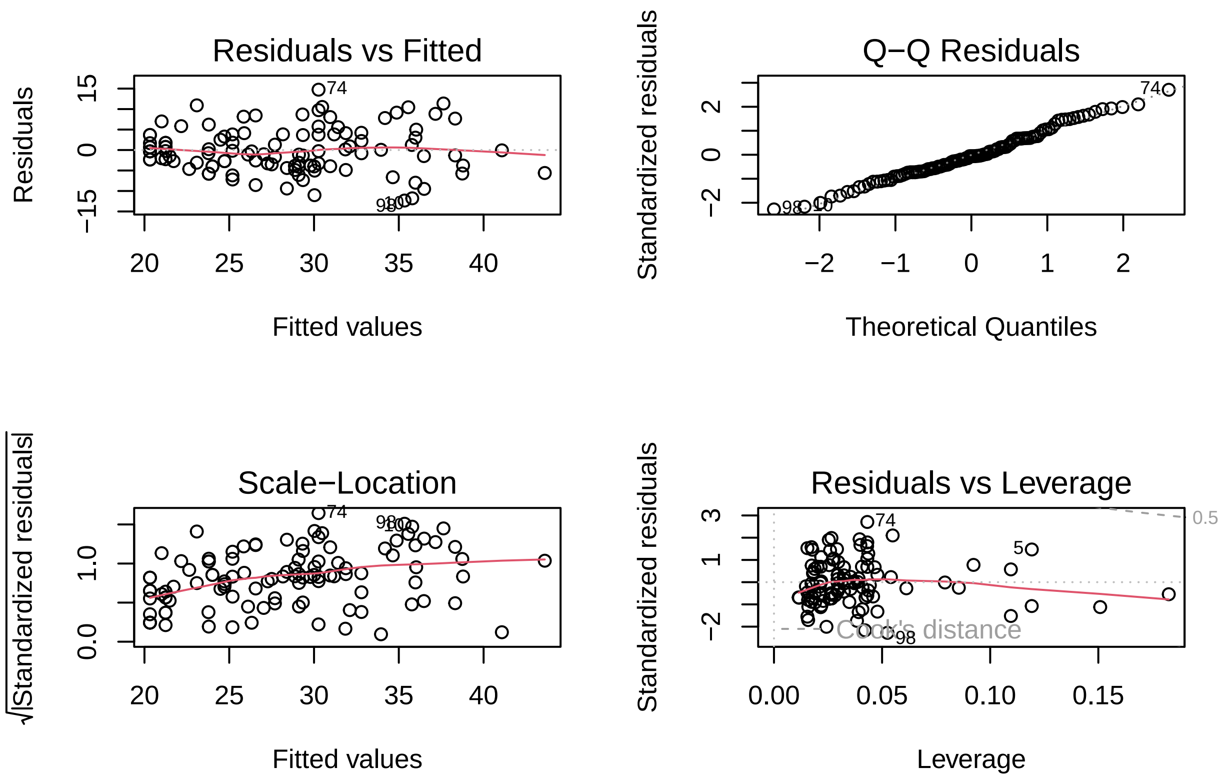


```r
# MCI
mci_data1 <- mci_data[, -c(4,13:30)]

null_m = lm(AES_TOT ~ 1. , data = mci_data1)
full_m = lm(AES_TOT ~ ., data = mci_data1)
step(null_m, scope = list(lower = null_m, upper = full_m),
     direction = "both")
```

```
## Start:  AIC=363.71
## AES_TOT ~ 1
```

```
##
##                  Df Sum of Sq    RSS    AIC
## + Hamilton        1   1811.67 6633.5 347.12
## + FAB             1    820.18 7625.0 357.84
## + Rehabilitation  1    709.57 7735.7 358.95
## + Moca            1    644.34 7800.9 359.60
## + Education       1    539.99 7905.2 360.62
## + MMSE            1    385.07 8060.1 362.12
## <none>                        8445.2 363.71
## + Comorbidity     1    214.57 8230.6 363.73
## + Sex             1    210.24 8235.0 363.77
## + Age             1    101.38 8343.8 364.78
## + Treatment       1     91.89 8353.3 364.87
##
## Step:  AIC=347.12
## AES_TOT ~ Hamilton
##
##                  Df Sum of Sq    RSS    AIC
## + MMSE            1    493.43 6140.1 343.17
## + Moca            1    488.19 6145.4 343.23
## + FAB             1    438.09 6195.5 343.86
## + Rehabilitation  1    270.28 6363.3 345.92
## + Education       1    231.57 6402.0 346.38
## <none>                        6633.5 347.12
## + Age             1    113.25 6520.3 347.79
## + Sex             1     34.45 6599.1 348.72
## + Treatment       1     13.16 6620.4 348.97
## + Comorbidity     1      0.02 6633.5 349.12
## - Hamilton        1   1811.67 8445.2 363.71
##
## Step:  AIC=343.17
## AES_TOT ~ Hamilton + MMSE
##
##                  Df Sum of Sq    RSS    AIC
## + Age             1    167.16 5973.0 343.04
## <none>                        6140.1 343.17
## + Rehabilitation  1    124.78 6015.3 343.59
## + FAB             1     81.85 6058.3 344.13
## + Moca            1     77.50 6062.6 344.19
## + Education       1     61.13 6079.0 344.40
## + Comorbidity     1      7.57 6132.5 345.07
## + Treatment       1      2.86 6137.2 345.13
## + Sex             1      0.40 6139.7 345.16
## - MMSE            1    493.43 6633.5 347.12
## - Hamilton        1   1920.03 8060.1 362.12
##
## Step:  AIC=343.04
## AES_TOT ~ Hamilton + MMSE + Age
##
##                  Df Sum of Sq    RSS    AIC
## <none>                        5973.0 343.04
## - Age             1    167.16 6140.1 343.17
## + Moca            1    124.32 5848.6 343.42
## + Rehabilitation  1     75.27 5897.7 344.07
```

```
## + FAB            1     65.44 5907.5 344.19
## + Education      1     52.28 5920.7 344.36
## + Comorbidity    1     13.84 5959.1 344.86
## + Sex            1      0.52 5972.4 345.04
## + Treatment      1      0.45 5972.5 345.04
## - MMSE           1    547.33 6520.3 347.79
## - Hamilton       1   1941.47 7914.4 362.71
```

```
##
## Call:
## lm(formula = AES_TOT ~ Hamilton + MMSE + Age, data = mci_data1)
##
## Coefficients:
## (Intercept)     Hamilton         MMSE          Age
##     63.0702       0.6486      -0.7897      -0.2022
```

```r
fit_MCI <- lm(AES_TOT ~ Hamilton + MMSE + Age, data = mci_data1)
summary(fit_MCI)
```

```
##
## Call:
## lm(formula = AES_TOT ~ Hamilton + MMSE + Age, data = mci_data1)
##
## Residuals:
##     Min      1Q  Median      3Q     Max
## -27.096  -5.709  -2.377   7.232  18.095
##
## Coefficients:
##             Estimate Std. Error t value Pr(>|t|)
## (Intercept)  63.0702    13.7779   4.578 1.89e-05 ***
## Hamilton      0.6486     0.1332   4.871 6.25e-06 ***
## MMSE         -0.7897     0.3053  -2.586   0.0117 *
## Age          -0.2022     0.1415  -1.429   0.1572
## ---
## Signif. codes:  0 '***' 0.001 '**' 0.01 '*' 0.05 '.' 0.1 ' ' 1
##
## Residual standard error: 9.046 on 73 degrees of freedom
## Multiple R-squared:  0.2927, Adjusted R-squared:  0.2637
## F-statistic: 10.07 on 3 and 73 DF,  p-value: 1.242e-05
```

```r
plot(fit_MCI)


# Alzheimer
alzheimer_data1 <- alzheimer_data[, -c(4,5,13:30)]

null_a = lm(AES_TOT ~ 1. , data = alzheimer_data1)
full_a = lm(AES_TOT ~ ., data = alzheimer_data1)
step(null_a, scope = list(lower = null_a, upper = full_a),
     direction = "both")
```

```
## Start:  AIC=143.32
```

```
## AES_TOT ~ 1
##
##                   Df Sum of Sq    RSS    AIC
## <none>                         3333.5 143.32
## + Hamilton        1   151.239 3182.3 143.92
## + Sex             1   116.591 3216.9 144.25
## + Rehabilitation  1    98.182 3235.3 144.42
## + Education       1    47.748 3285.8 144.88
## + FAB             1    46.465 3287.0 144.90
## + Age             1    36.940 3296.6 144.98
## + MMSE            1    15.004 3318.5 145.18
## + Moca            1    13.130 3320.4 145.20
```

```
##
## Call:
## lm(formula = AES_TOT ~ 1, data = alzheimer_data1)
##
## Coefficients:
## (Intercept)
##        42.5
```

```
fit_alzheimer <- lm(AES_TOT ~ 1, data = alzheimer_data1)
summary(fit_alzheimer)
```

```
##
## Call:
## lm(formula = AES_TOT ~ 1, data = alzheimer_data1)
##
## Residuals:
##    Min      1Q Median     3Q    Max
## -18.50  -8.25  -2.00   8.50  24.50
##
## Coefficients:
##             Estimate Std. Error t value Pr(>|t|)
## (Intercept)   42.500      1.957   21.71   <2e-16 ***
## ---
## Signif. codes:  0 '***' 0.001 '**' 0.01 '*' 0.05 '.' 0.1 ' ' 1
##
## Residual standard error: 10.72 on 29 degrees of freedom
```

```
plot(fit_MCI)
```

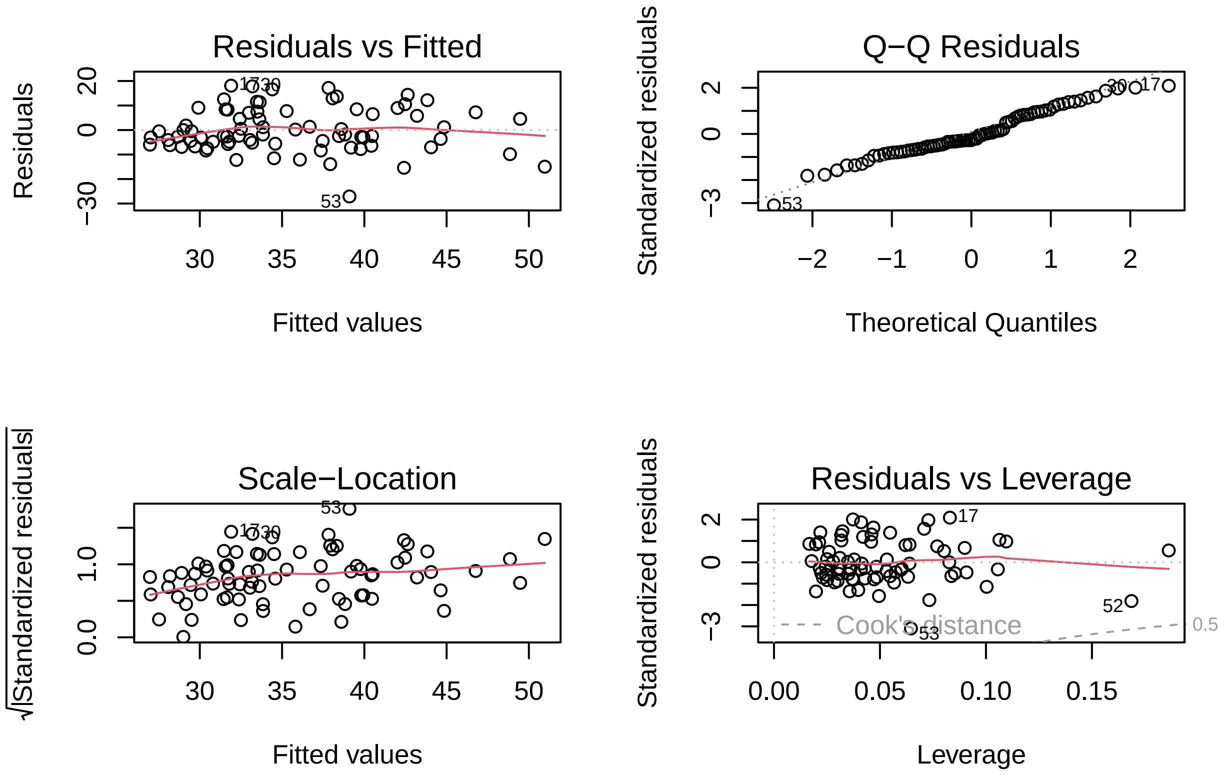


```r
# CLUSTERING ANALYSIS in the dataset to see if the three groups have very
# different characteristics on which they can be separated.

library(ggplot2)
str(data1)
```

```
## tibble [214 x 12] (S3: tbl_df/tbl/data.frame)
##  $ Age           : num [1:214] 80 67 75 88 71 70 77 78 83 66 ...
##  $ Sex           : num [1:214] 0 1 0 1 0 0 0 0 0 0 ...
##  $ Education     : num [1:214] 8 4 5 18 15 13 5 13 8 8 ...
##  $ Diagnosis     : num [1:214] 1 1 1 1 1 1 1 1 1 1 ...
##  $ Comorbidity   : num [1:214] 0 1 1 1 0 0 0 0 0 0 ...
##  $ Treatment     : num [1:214] 0 1 0 0 0 0 0 1 1 1 ...
##  $ Rehabilitation: num [1:214] 1 1 1 0 0 0 0 0 1 1 ...
##  $ MMSE          : num [1:214] 20 26 21 24 26 27 18 23 22 20 ...
##  $ Moca          : num [1:214] 14 25 14 15 20 15 9 13 11 12 ...
##  $ FAB           : num [1:214] 8 17 12 13 14 13 6 11 10 4 ...
##  $ Hamilton      : num [1:214] 3 14 11 17 0 14 2 23 17 23 ...
##  $ AES_TOT       : num [1:214] 29 51 36 29 22 38 29 37 37 39 ...
```

```r
View(data1)

data1$Sex <- factor(data1$Sex, levels = c(0,1),
                   labels = c("Female", "Male"))
data1$Diagnosis <- factor(data1$Diagnosis, levels = c(0,1,2),
```

```r
                         labels = c("Control", "MCI", "Alzheimer"))
data1$Comorbidity <- factor(data1$Comorbidity, levels = c(0,1),
                           labels = c("Absence of Comorbidity",
                                      "Presence of Comorbidity"))
data1$Treatment <- factor(data1$Treatment, levels = c(0,1),
                         labels = c("No treatment", "Under treatment"))
data1$Rehabilitation <- factor(data1$Rehabilitation, levels = c(0,1),
                              labels = c("No rehab", "Under rehab"))

ggplot(data = data1, aes(Moca, AES_TOT)) +
  geom_point(aes(colour = Diagnosis))
```

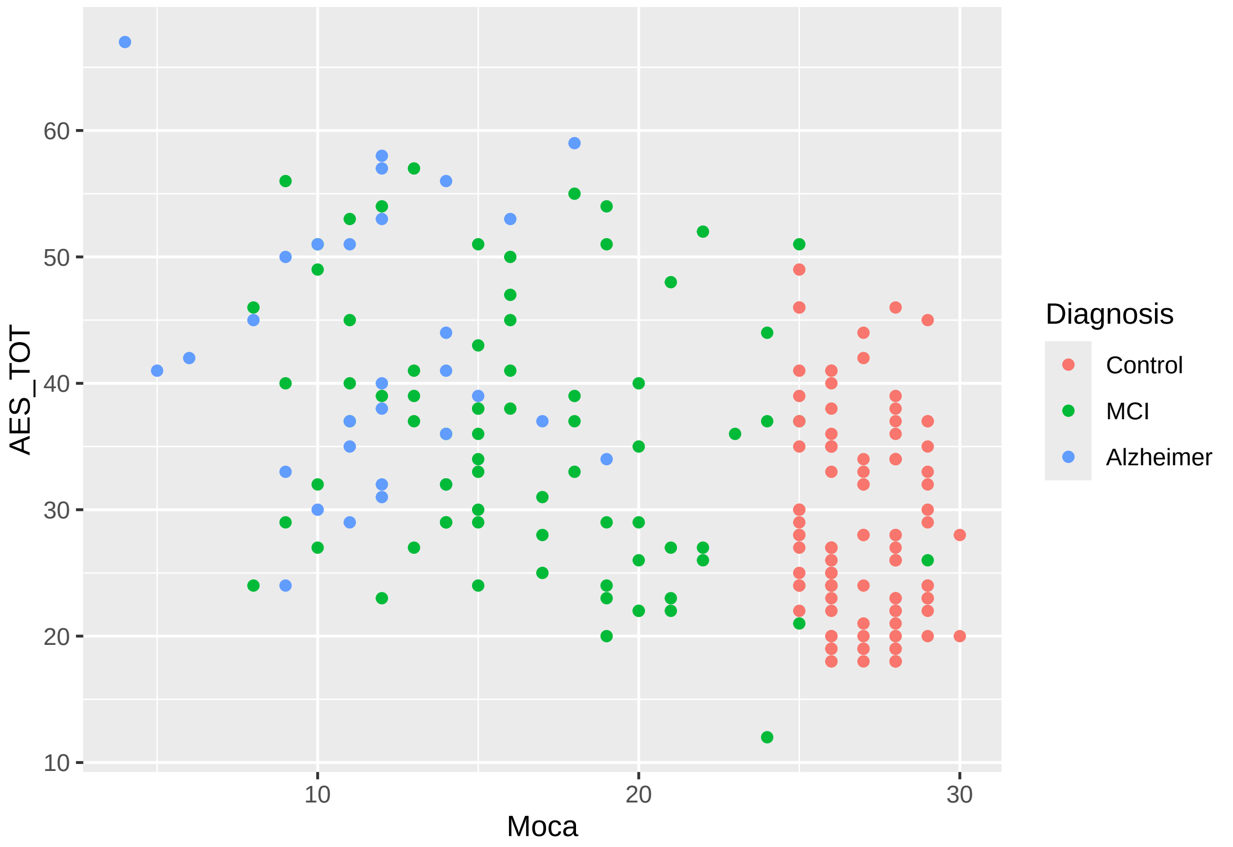


```r
set.seed(21)
data_cluster <- kmeans(data1[, c(12,9)], 3, nstart = 15)
data_cluster
```

```
## K-means clustering with 3 clusters of sizes 36, 85, 93
##
## Cluster means:
##    AES_TOT     Moca
## 1 50.72222 14.22222
## 2 35.41176 18.82353
## 3 23.84946 25.82796
##
## Clustering vector:
```

```
##   [1] 2 1 2 2 3 2 2 2 2 2 1 1 1 2 2 2 2 3 1 3 2 2 2 1 3 3 2 2 1 2 3 2 2 1 3 3 1
##  [38] 3 3 2 2 2 2 2 2 1 2 1 1 2 2 2 2 2 2 1 2 1 3 1 2 2 3 1 3 2 3 2 2 1 2 2 3 2
##  [75] 2 2 2 3 2 2 2 1 3 1 1 2 1 2 1 1 1 1 1 2 1 1 2 1 1 2 2 2 3 2 1 1 1 3 3 3 2
## [112] 1 1 3 3 3 3 3 3 3 3 3 3 3 3 3 3 2 3 3 3 3 3 3 3 3 3 2 3 3 2 3 2 3 2 3 3 3
## [149] 3 3 3 3 3 3 3 3 3 3 2 2 1 2 3 2 2 2 2 2 2 2 2 3 3 2 2 2 3 2 3 3 3 2 2 2 3 2
## [186] 3 3 3 3 2 3 3 3 3 3 3 3 3 3 2 3 3 3 3 3 2 3 3 3 3 2 3 2 3
##
## Within cluster sum of squares by cluster:
## [1] 2201.444 5668.941 2397.140
##  (between_SS / total_SS =  69.9 %)
##
## Available components:
##
## [1] "cluster"      "centers"      "totss"        "withinss"     "tot.withinss"
## [6] "betweenss"    "size"         "iter"         "ifault"
```

```r
table(data_cluster$cluster, data1$Diagnosis)
```

```
##
##     Control MCI Alzheimer
##   1       3  19        14
##   2      29  40        16
##   3      75  18         0
```

```r
data_cluster$cluster <- as.factor(data_cluster$cluster)
ggplot(data1, aes(Moca, AES_TOT, color = data_cluster$cluster)) +
  geom_point()
```

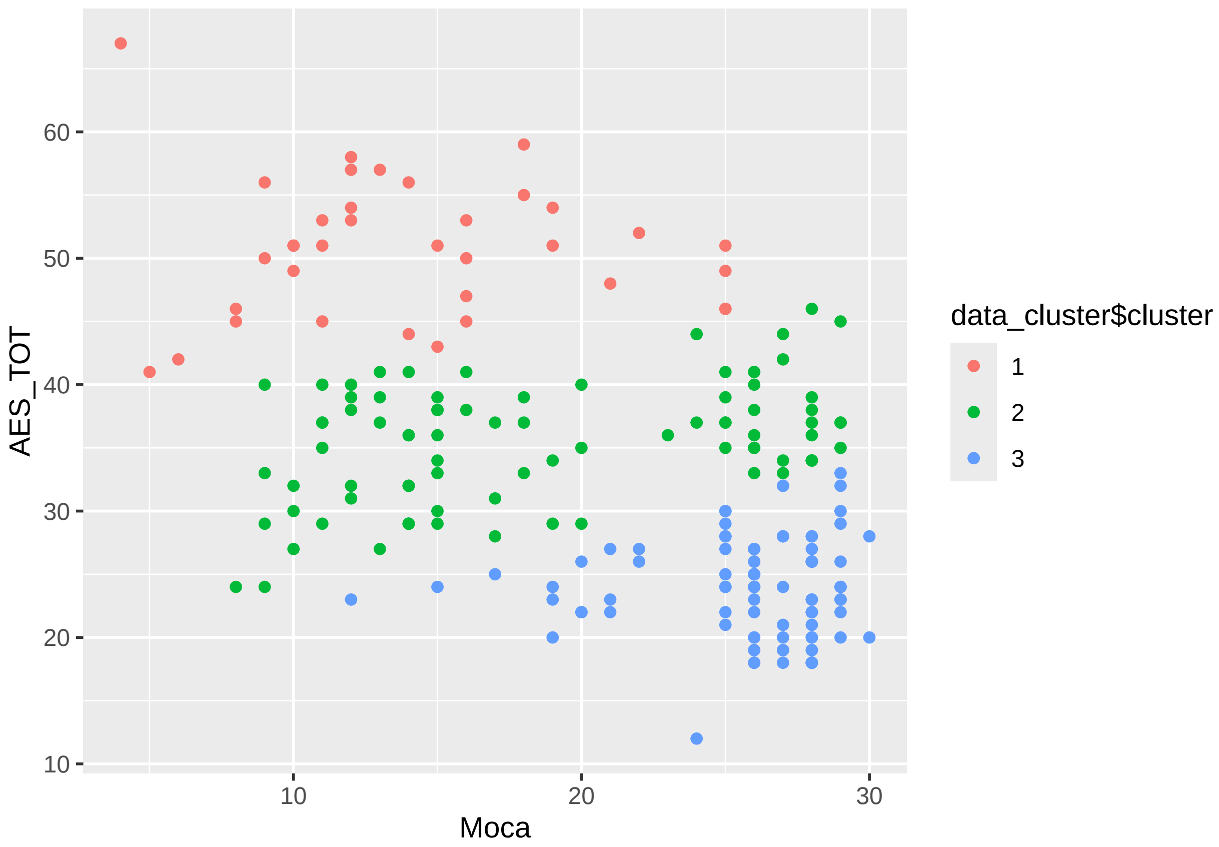


```
# PRINCIPAL COMPONENT ANALYSIS
log.x <- log(data1[, c(1,3,8:12)])
View(log.x)
data_diagnosis <- data1[, 4]

# Eliminate all logarithms that have Inf. value. 189 observations left
log.x <- log.x[which(apply(log.x, 1, \(i) !any(i == "-Inf"))),]

data.pca <- prcomp(log.x,
                   center = TRUE,
                   scale. = TRUE)

print(data.pca)
```

```
## Standard deviations (1, .., p=7):
## [1] 1.8195121 1.0601120 0.9572458 0.8792278 0.7256000 0.4787121 0.3471549
##
## Rotation (n x k) = (7 x 7):
##                  PC1         PC2        PC3           PC4         PC5
## Age       -0.1566450 -0.70061042  0.3088370  0.5592470219 -0.26222767
## Education  0.2635500  0.23318411 -0.6616285  0.5918222771 -0.24167045
## MMSE       0.4766555  0.11318689  0.3406389  0.0027075623  0.02090378
## Moca       0.4994218  0.05777994  0.3165541 -0.0006098696 -0.04166245
## FAB        0.4888858  0.05891360  0.1927704  0.1151086302 -0.22995322
## Hamilton  -0.2659498  0.49221189  0.3800379  0.5645450378  0.46231034
## AES_TOT   -0.3458939  0.43917771  0.2627487 -0.0708545091 -0.77720653
```

```
##                    PC6          PC7
## Age        0.086791517  0.013181908
## Education  0.158305188  0.068523815
## MMSE       0.673700131 -0.435473591
## Moca      -0.002574516  0.803299491
## FAB       -0.704011901 -0.398243976
## Hamilton  -0.100544834  0.004263686
## AES_TOT    0.088239359  0.039836668
```

```
plot(data.pca, type = "l") #see which Principal Component explains maximum
```

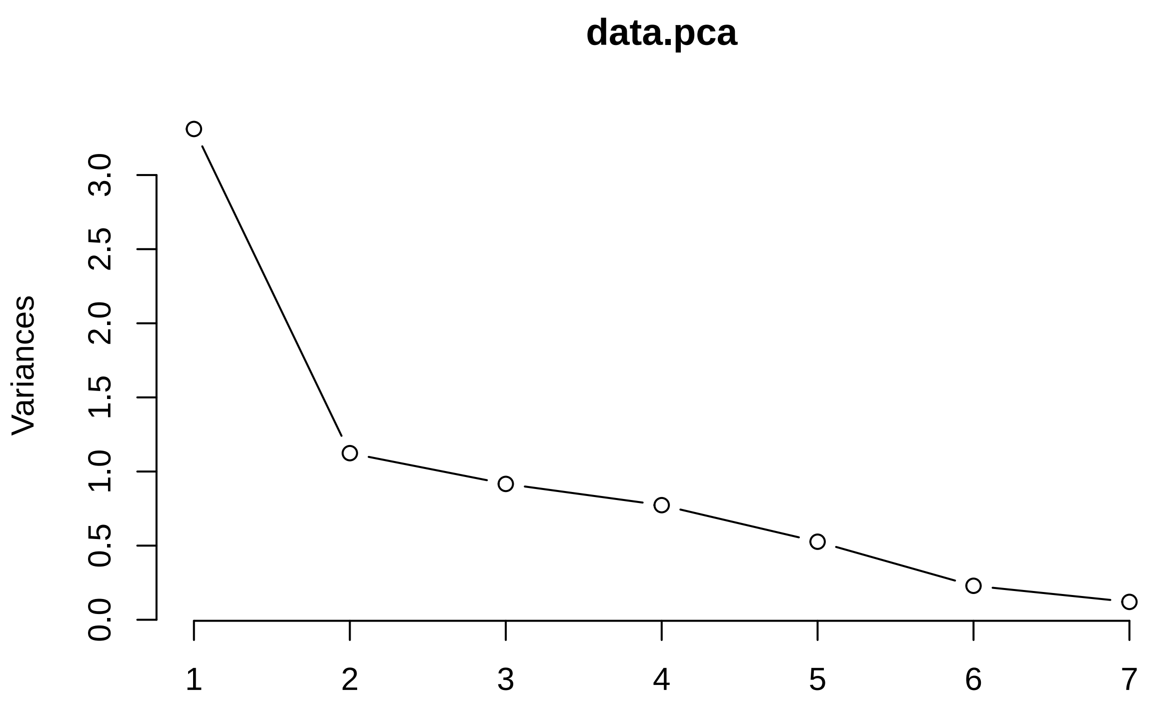


```
# variability in data for margin error
par("mar")
```

```
## [1] 5.1 4.1 4.1 2.1
```

```
par(mar=c(1,1,1,1))

summary(data.pca)
```

```
## Importance of components:
##                           PC1    PC2    PC3    PC4     PC5     PC6     PC7
## Standard deviation     1.8195 1.0601 0.9572 0.8792 0.72560 0.47871 0.34715
## Proportion of Variance 0.4729 0.1605 0.1309 0.1104 0.07521 0.03274 0.01722
## Cumulative Proportion  0.4729 0.6335 0.7644 0.8748 0.95005 0.98278 1.00000
```

```r
library(devtools)
# install_github("fawda123/ggord")
library(ggord)

data2 <- data1[-c(5,80,98,101,109,110,116,134,136,139,140,152,158,162,178,184,
                  188,191,192,194,196:199,201) ,]
str(data2)
```

```
## tibble [189 x 12] (S3: tbl_df/tbl/data.frame)
##  $ Age           : num [1:189] 80 67 75 88 70 77 78 83 66 83 ...
##  $ Sex           : Factor w/ 2 levels "Female","Male": 1 2 1 2 1 1 1 1 1 1 ...
##  $ Education     : num [1:189] 8 4 5 18 13 5 13 8 8 5 ...
##  $ Diagnosis     : Factor w/ 3 levels "Control","MCI",..: 2 2 2 2 2 2 2 2 2 2 ...
##  $ Comorbidity   : Factor w/ 2 levels "Absence of Comorbidity",..: 1 2 2 2 1 1 1 1 1 1 ...
##  $ Treatment     : Factor w/ 2 levels "No treatment",..: 1 2 1 1 1 1 2 2 2 2 ...
##  $ Rehabilitation: Factor w/ 2 levels "No rehab","Under rehab": 2 2 2 1 1 1 1 2 2 2 ...
##  $ MMSE          : num [1:189] 20 26 21 24 27 18 23 22 20 18 ...
##  $ Moca          : num [1:189] 14 25 14 15 15 9 13 11 12 16 ...
##  $ FAB           : num [1:189] 8 17 12 13 13 6 11 10 4 8 ...
##  $ Hamilton      : num [1:189] 3 14 11 17 14 2 23 17 23 13 ...
##  $ AES_TOT       : num [1:189] 29 51 36 29 38 29 37 37 39 47 ...
```

```r
p <- ggord(data.pca, data2$Diagnosis)
p
```

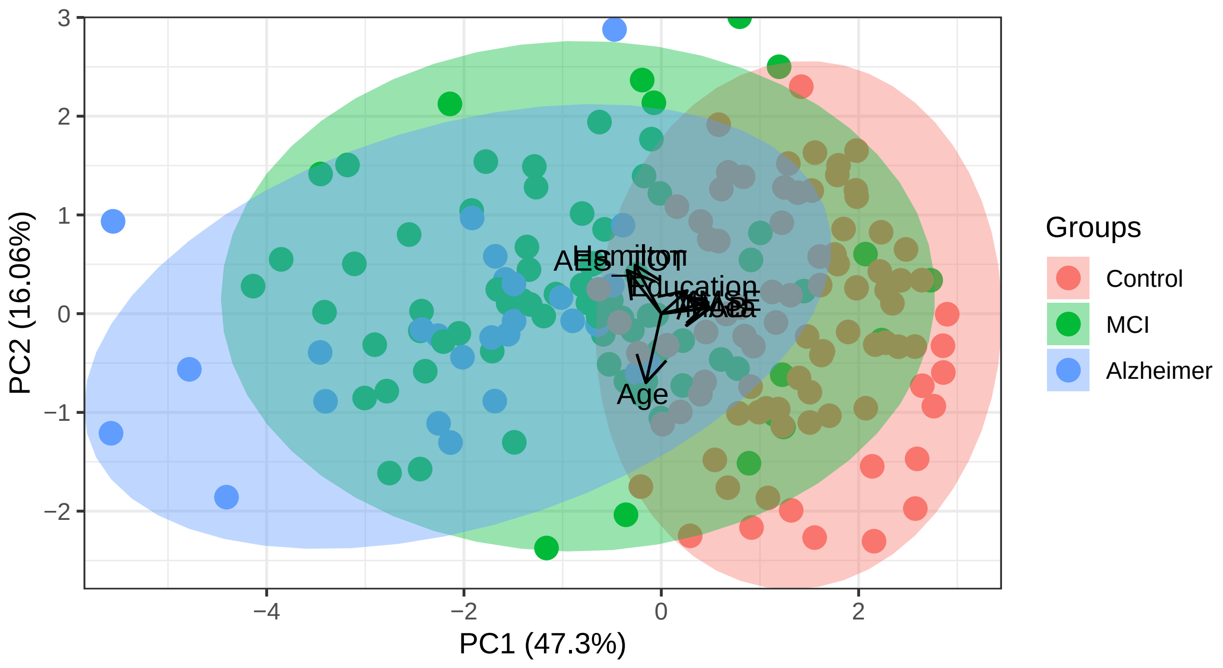

Supplement: Supplementary file 3 — Supplementary Material 3. [file 40359_2024_2239_MOESM3_ESM.pdf]
