# Supplementary material for: Exploring apathy components and their relationship in cognitive decline: insights from a network cross-sectional study
Source: BMC Psychol. 2025 Feb 17;13:129. doi: 10.1186/s40359-024-02239-x (PMC11834197; doi:10.1186/s40359-024-02239-x)

Rcode of Network Analysis - Exploring apathy components and their relationship in cognitive decline: insights from a network analysis study -

Pierfrancesco Sarti and Sophie Tascedda

GENERAL NETWORK MODELS

```r
library(readxl)

data_Control <- read_excel("data_Control.xlsx")
data_MCI <- read_excel("data_MCI.xlsx")
data_Alzheimer <- read_excel("data_Alzheimer.xlsx")

data_Control <- data_Control[, -c(2:19, 24:26)]
data_MCI <- data_MCI[, -c(2:19)]
data_Alzheimer <- data_Alzheimer[, -c(2:19)]
data_Alzheimer <- data_Alzheimer[, -c(7)]

C_mat <- as.matrix(data_Control)
MCI_mat <- as.matrix(data_MCI)
A_mat <- as.matrix(data_Alzheimer)

groups_C <- c(rep("Apathy Evaluation Scale",1), rep ("Depression",1),
              rep("General Informations",3), rep("Neurocognitive",3))

groups <- c(rep("Apathy Evaluation Scale",1), rep("Depression",1),
            rep("General Informations",5), rep("Neurocognitive",3))

col = c("grey", "lightskyblue", "olivedrab1", "firebrick1")
```

Mixed Graphical Model for the CONTROL GROUP

```r
library(mgm)
library(qgraph)
library(networktools)
library(igraph)
library(bootnet)

set.seed(321)

net_C <- mgm(data = C_mat, alphaSel = "EBIC",
             type = c("g","g","g","c","g","g","g","g"),
             level = c("1","1","1","2","1","1","1","1"),
             k = 2, ruleReg = "OR", alphaSeq = 1, scale = TRUE,
             method = "glm", pbar = FALSE)
```

```
## Note that the sign of parameter estimates is stored separately; see ?mgm
```

```r
##### Representation of the MGM
qgraph_C <- qgraph(net_C$pairwise$wadj, layout = "circle",
                   edge.color = net_C$pairwise$edgecolor,
                   groups = groups_C, color = col,
```

```
                    nodeNames = colnames(C_mat), legend.mode = "style2",
                    legend.cex = 0.3, vTrans = 200,
                    vsize = 5.5, esize = 25)
```

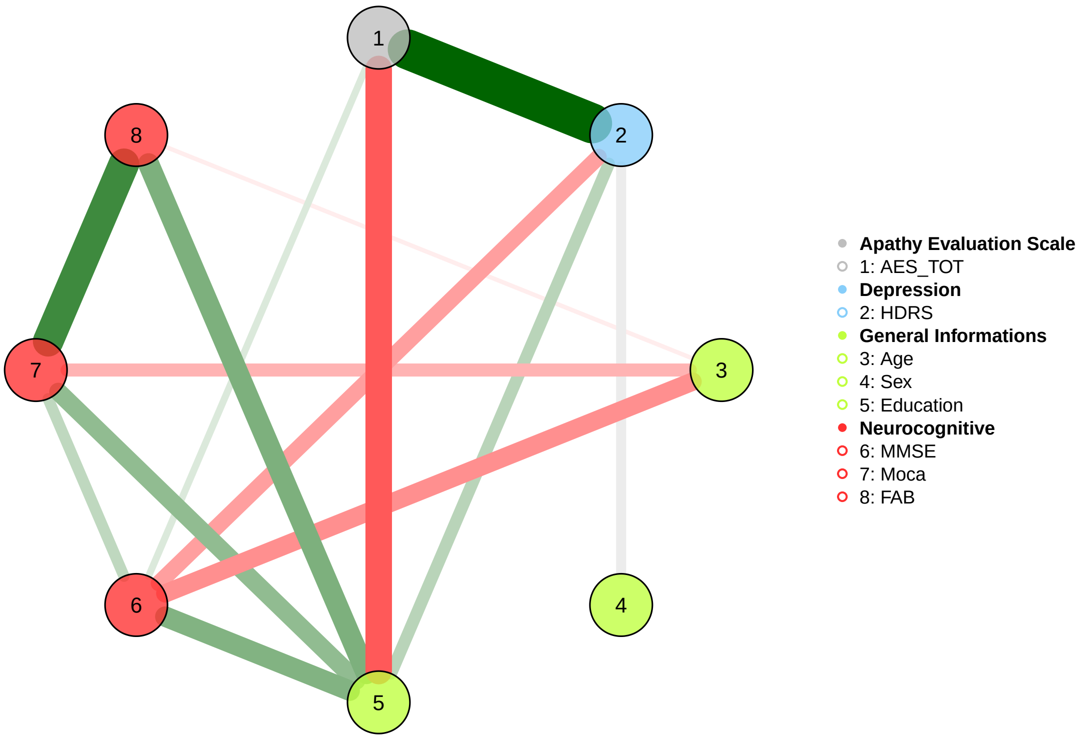


```
c <- ncol(C_mat)

pred_objControl <- predict(object = net_C, data = C_mat,
                           errorCat = c("CC", "nCC", "CCmarg"),
                           errorCon = c("R2"))
pred_objControl$errors
```

```
##    Variable    R2    CC nCC CCmarg
## 1   AES_TOT 0.491    NA  NA     NA
## 2      HDRS 0.401    NA  NA     NA
## 3       Age 0.280    NA  NA     NA
## 4       Sex    NA 0.729   0  0.729
## 5 Education 0.623    NA  NA     NA
## 6      MMSE 0.437    NA  NA     NA
## 7      Moca 0.592    NA  NA     NA
## 8       FAB 0.588    NA  NA     NA
```

```
errorsControl <- list()
for(i in 1:3) errorsControl[[i]] <- pred_objControl$errors[i,2]
for(i in 4) errorsControl[[i]] <- pred_objControl$errors[i,3]
```

```r
for(i in 5:8) errorsControl[[i]] <- pred_objControl$errors[i,2]

color_listControl <- list()
for(i in 1:8) color_listControl[[i]] <- "black"

#### Representation of the resulting network with predictability measures
Control <- qgraph(net_C$pairwise$wadj,
                  layout = "circle", groups = groups_C,
                  vsize = 6, edge.color = net_C$pairwise$edgecolor,
                  color = col, nodeNames = colnames(C_mat),
                  legend.mode = "style2",
                  legend.cex = 0.3, vTrans = 200, vsize = 5.5, esize = 25,
                  pie = errorsControl, pieColor = color_listControl,
                  title = "Control group")
```

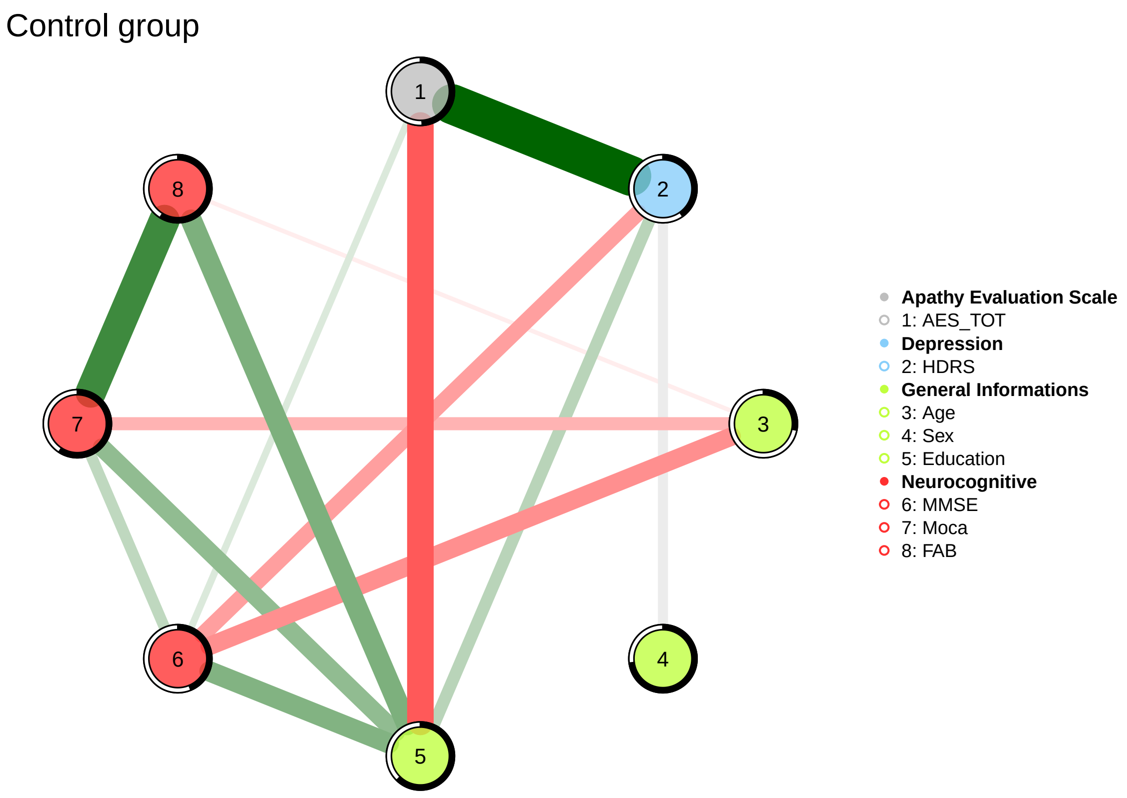

Mixed Graphical Model for the MCI Patients

```r
set.seed(202)
net_M <- mgm(data = MCI_mat, alphaSel = "CV", alphaFolds = 10,
             type = c("g","g","g","g","g","c","c","c","g","g","g"),
             level = c("1","1","1","1","1","2","2","2","1","1","1"),
```

```r
              k = 2, ruleReg = "OR", alphaSeq = 1, scale = TRUE,
              method = "glm", pbar = FALSE)
```

```
## Note that the sign of parameter estimates is stored separately; see ?mgm
```

```r
#### Representation of the MGM
qgraph_M <- qgraph(net_M$pairwise$wadj, layout = "circle",
                   edge.color = net_M$pairwise$edgecolor,
                   groups = groups, color = col,
                   nodeNames = colnames(MCI_mat), legend.mode = "style2",
                   legend.cex = 0.3, vTrans = 200,
                   vsize = 5.5, esize = 25)
```

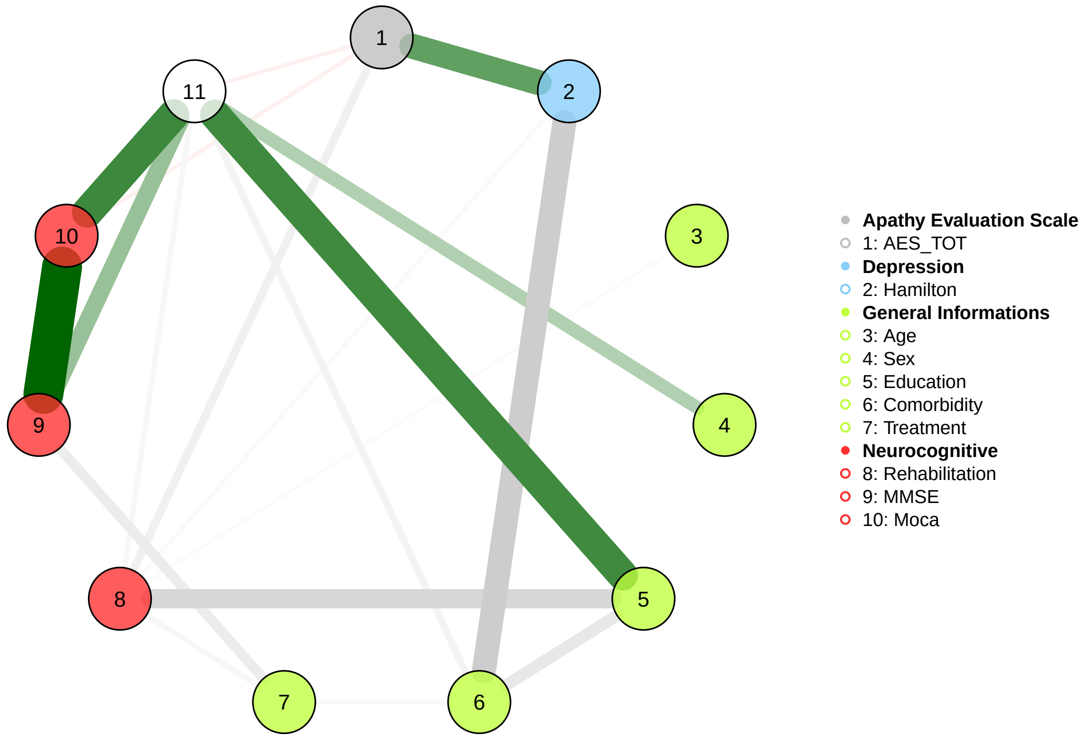


```r
#### Calculating the Predictability of each node
m <- ncol(MCI_mat)

pred_objMCI <- predict(object = net_M, data = MCI_mat,
                       errorCat = c("CC", "nCC", "CCmarg"),
                       errorCon = c("R2"))
pred_objMCI$errors
```

```
##          Variable    R2    CC   nCC CCmarg
## 1         AES_TOT 0.242    NA    NA     NA
## 2        Hamilton 0.265    NA    NA     NA
```

```
## 3             Age 0.000    NA   NA     NA
## 4             Sex 0.078    NA   NA     NA
## 5       Education 0.365    NA   NA     NA
## 6     Comorbidity    NA 0.753 0.00  0.753
## 7       Treatment    NA 0.662 0.00  0.662
## 8  Rehabilitation    NA 0.688 0.25  0.584
## 9            MMSE 0.569    NA   NA     NA
## 10           Moca 0.619    NA   NA     NA
## 11            FAB 0.629    NA   NA     NA
```

```
errorsMCI <- list()
for(i in 1:5) errorsMCI[[i]] <- pred_objMCI$errors[i,2]
for(i in 6:8) errorsMCI[[i]] <- pred_objMCI$errors[i,4]
for(i in 9:11) errorsMCI[[i]] <- pred_objMCI$errors[i,2]

color_listMCI <- list()
for(i in 1:11) color_listMCI[[i]] <- "black"


##### Representation of the resulting network with predictability measures
MCI <- qgraph(net_M$pairwise$wadj,
              layout = "circle", groups = groups,
              vsize = 6, edge.color = net_M$pairwise$edgecolor,
              color = col, nodeNames = colnames(MCI_mat),
              legend.mode = "style2",
              legend.cex = 0.3, vTrans = 200, vsize = 5.5, esize = 25,
              pie = errorsMCI, pieColor = color_listMCI,
              title = "Mild Cognitive Impairment")
```

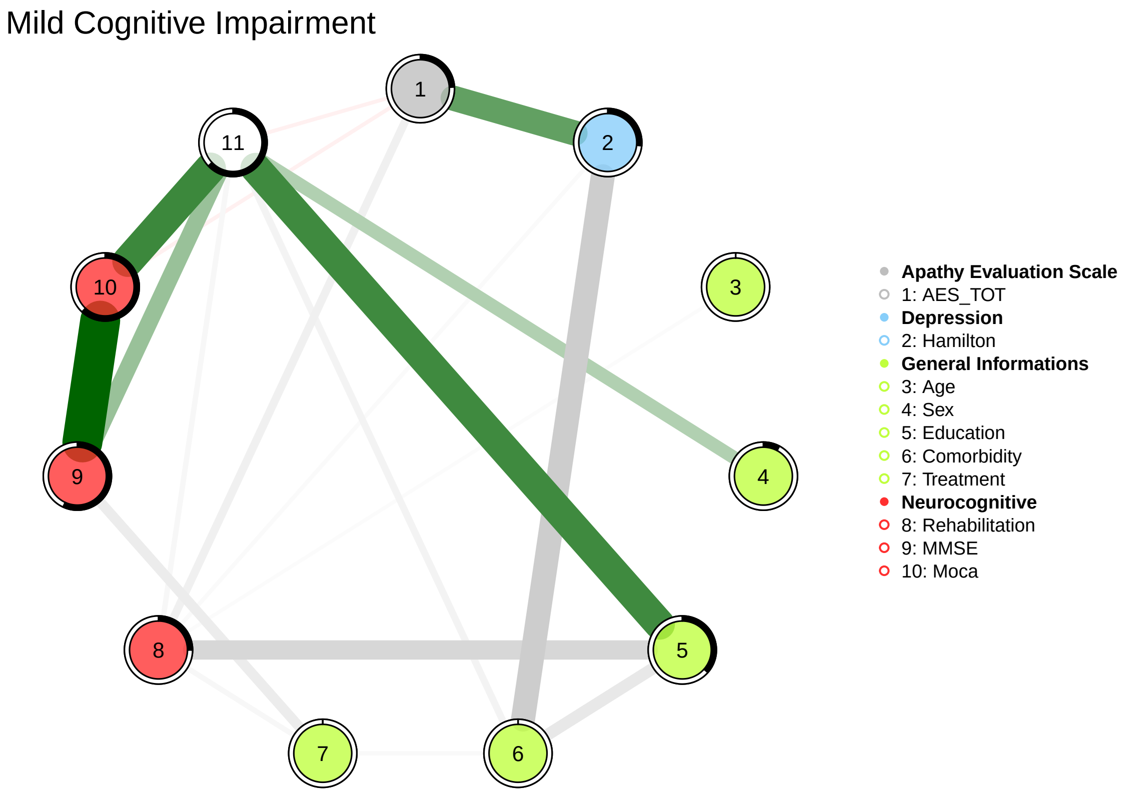

Mixed Graphical Model for the ALZHEIMER Patients

```r
set.seed(127)
net_A <- mgm(data = A_mat, alphaSel = "CV", alphaFolds = 10,
             type = c("g","g","g","g","g","c","c","g","g","g"),
             level = c("1","1","1","1","1","2","2","1","1","1"),
             k = 2, ruleReg = "OR", alphaSeq = 1, scale = TRUE,
             method = "glm", pbar = FALSE)
```

```
## Note that the sign of parameter estimates is stored separately; see ?mgm
```

```r
#### Representation of the MGM
qgraph_A <- qgraph(net_A$pairwise$wadj, layout = "circle",
                   edge.color = net_A$pairwise$edgecolor,
                   groups = groups, color = col,
                   nodeNames = colnames(A_mat), legend.mode = "style2",
                   legend.cex = 0.3, vTrans = 200,
                   vsize = 5.5, esize = 25)
```

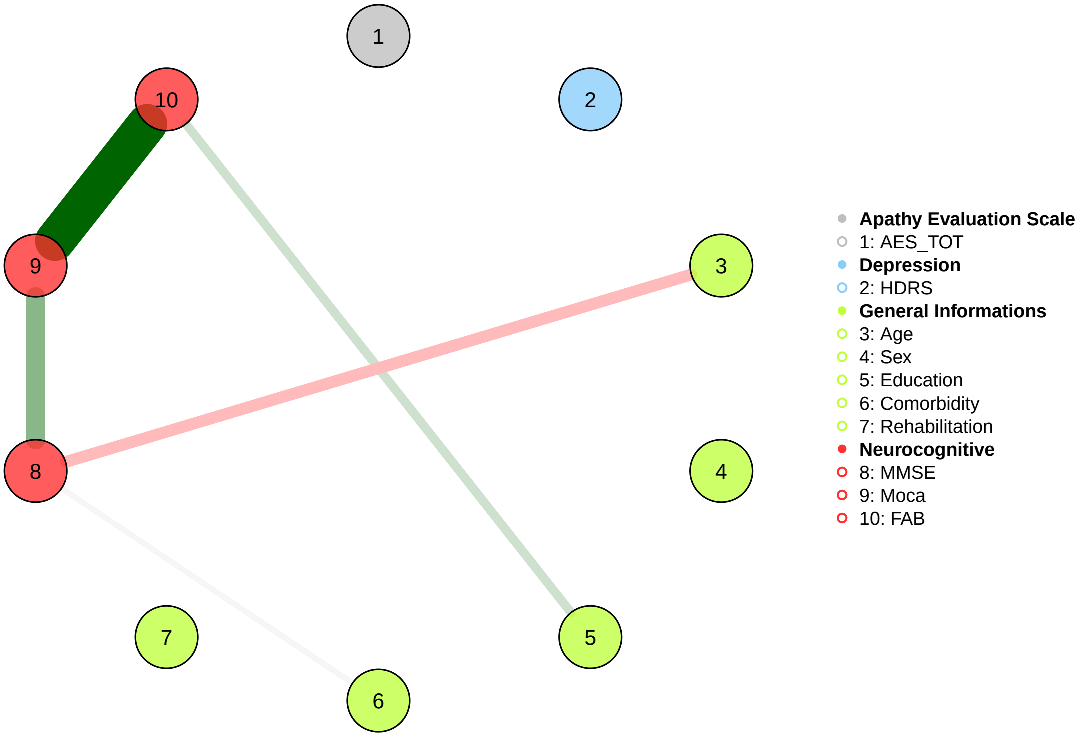


```r
#### Calculating the Predictability of each node
a <- ncol(A_mat)

pred_objAlz <- predict(object = net_A, data = A_mat,
                       errorCat = c("CC", "nCC", "CCmarg"),
                       errorCon = c("R2"))
pred_objAlz$errors
```

```
##          Variable    R2    CC nCC CCmarg
## 1         AES_TOT 0.000    NA  NA     NA
## 2            HDRS 0.000    NA  NA     NA
## 3             Age 0.122    NA  NA     NA
## 4             Sex 0.000    NA  NA     NA
## 5       Education 0.132    NA  NA     NA
## 6     Comorbidity    NA 0.700   0  0.700
## 7  Rehabilitation    NA 0.733   0  0.733
## 8            MMSE 0.297    NA  NA     NA
## 9            Moca 0.586    NA  NA     NA
## 10            FAB 0.519    NA  NA     NA
```

```r
errorsAlz <- list()
for(i in 1:5) errorsAlz[[i]] <- pred_objAlz$errors[i,2]
for(i in 6:7) errorsAlz[[i]] <- pred_objAlz$errors[i,4]
for(i in 8:10) errorsAlz[[i]] <- pred_objAlz$errors[i,2]
```

```r
color_listAlz <- list()
for(i in 1:10) color_listAlz[[i]] <- "black"


# Representation of the resulting network with predictability measures

Alzheimer <- qgraph(net_A$pairwise$wadj,
                    layout = "circle", groups = groups,
                    vsize = 6, edge.color = net_A$pairwise$edgecolor,
                    color = col, nodeNames = colnames(A_mat),
                    legend.mode = "style2",
                    legend.cex = 0.3, vTrans = 200, vsize = 5.5, esize = 25,
                    pie = errorsAlz, pieColor = color_listAlz,
                    title = "Alzheimer")
```

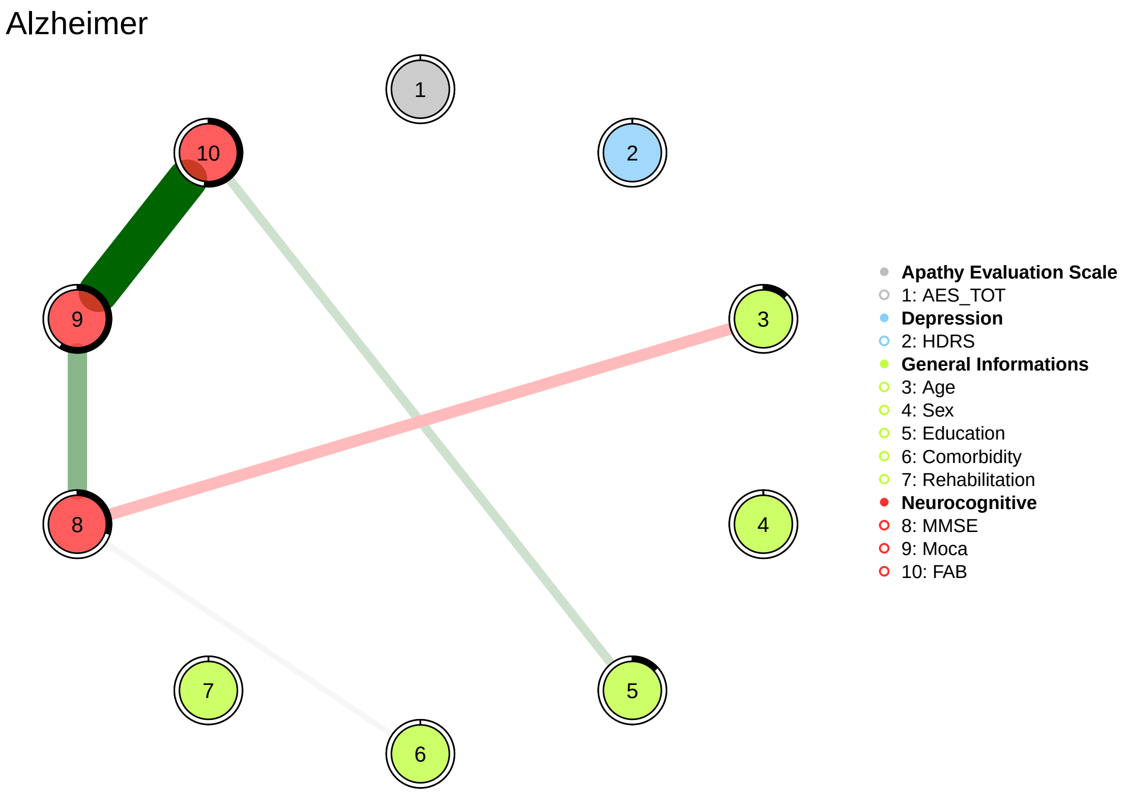


```r
# PDF with the networks not exploring the Apathy Scale

# pdf("General Networks.pdf", width = 25)
# par(mfrow = c(1,3))
# qgraph(net_C$pairwise$wadj,
#        layout = "circle", groups = groups_C,
#        vsize = 10, edge.color = net_C$pairwise$edgecolor,
#        color = col, nodeNames = colnames(C_mat),
#        legend.mode = "style2",
#        vTrans = 200, vsize = 5.5, esize = 20,
```

```r
#        pie = errorsControl, pieColor = color_listControl,
#        title = "Control group")
# qgraph(net_M$pairwise$wadj,
#        layout = "circle", groups = groups,
#        vsize = 10, edge.color = net_M$pairwise$edgecolor,
#        color = col, nodeNames = colnames(MCI_mat),
#        legend.mode = "style2",
#        vTrans = 200, usize = 5.5, esize = 20,
#        pie = errorsMCI, pieColor = color_listMCI,
#        title = "Mild Cognitive Impairment")
# qgraph(net_A$pairwise$wadj,
#        layout = "circle", groups = groups,
#        vsize = 10, edge.color = net_A$pairwise$edgecolor,
#        color = col, nodeNames = colnames(A_mat),
#        legend.mode = "style2",
#        vTrans = 200, usize = 5.5, esize = 20,
#        pie = errorsAlz, pieColor = color_listAlz,
#        title = "Alzheimer")
# dev.off()
```

NETWORK MODELS WITH ALL ITEMS OF APATHY EVALUATION SCALE

Directory and Uploading the datasets

```
load(file = "mark_env.RData")
library(readxl)
data_Control <- read_excel("data_Control.xlsx")
data_MCI <- read_excel("data_MCI.xlsx")
data_Alzheimer <- read_excel("data_Alzheimer.xlsx")
```

Looking for NAs and the structure of the dataset!

```
# is.na(data_Control)
# is.na(data_MCI)
# is.na(data_Alzheimer) # No NAs in the datasets
#
# str(data_Control)
# str(data_MCI)
# str(data_Alzheimer)
```

Deleting the column AES_TOT in both datasets for not plotting boosted edges with the single Scale Items.
Deleting the variables Treatment, Rehabilitation, Comorbidity, form the control group because non of the control had one of these.
Deleting the variable Treatment from Alzheimer group because non of them had a pharmacological treatment.

```
C <- data_Control[, c(-1,-24,-25,-26,-27)]
A <- data_Alzheimer[, c(-1,-25,-27)]
M <- data_MCI[, c(-1,-27)]
```

From datasets to Matrices

```
C <- as.matrix(C)
A <- as.matrix(A)
M <- as.matrix(M)
```

Retrieving column names

```
names <- colnames(A)
names_Control <- colnames(C)
names_MCI <- colnames(M)
```

Grouping the variables in specific domains + colours

```
groups_MCI <- c(rep("Apathy Evaluation Scale",18), rep("Depression",1),
            rep("General Informations",6), rep("Neurocognitive",2))

groups_C <- c(rep("Apathy Evaluation Scale",18), rep ("Depression",1),
              rep("General Informations",3), rep("Neurocognitive",2))
```

```r
groups_A <- c(rep("Apathy Evaluation Scale",18), rep("Depression",1),
              rep("General Informations",5), rep("Neurocognitive",2))

col = c("grey", "lightskyblue", "olivedrab1", "firebrick1")
```

Libraries that will be used for Network Analysis

```r
library(mgm)
library(qgraph)
library(networktools)
library(igraph)
library(bootnet)
```

Mixed Graphical Model for the CONTROL GROUP

```r
# set.seed(113)
# net_C <- mgm(data = C, alphaSel = "EBIC",
#              type = c("g","g","g","g","g","g","g","g","g","g","g","g","g",
#                       "g","g","g","g","g","g","g","c","g","g","g"),
#              level = c("1","1","1","1","1","1","1","1","1","1","1","1","1",
#                        "1","1","1","1","1","1","1","2","1","1","1"),
#              k = 2, ruleReg = "OR", alphaSeq = 1, scale = TRUE,
#              method = "glm", pbar = FALSE)
```

Representation of the MGM

```r
qgraph_C <- qgraph(net_C$pairwise$wadj, layout = "spring",
                   edge.color = net_C$pairwise$edgecolor,
                   groups = groups_C, color = col,
                   nodeNames = names_Control, legend.mode = "style2",
                   legend.cex = 0.3, vTrans = 200,
                   vsize = 5.5, esize = 25)
```

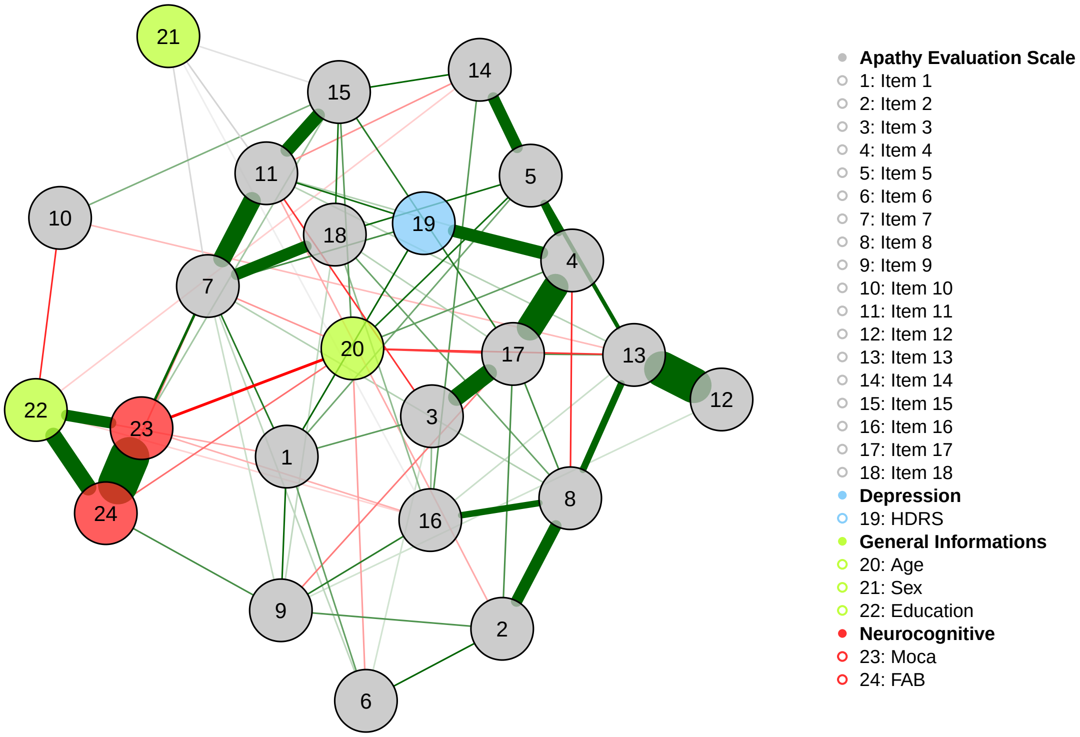

Calculating the Predictability of each node

```
c <- ncol(C)

pred_objControl <- predict(object = net_C, data = C,
                           errorCat = c("CC", "nCC", "CCmarg"),
                           errorCon = c("R2"))
pred_objControl$errors
```

```
##      Variable    R2    CC  nCC  CCmarg
## 1      Item 1 0.515    NA   NA      NA
## 2      Item 2 0.369    NA   NA      NA
## 3      Item 3 0.405    NA   NA      NA
## 4      Item 4 0.615    NA   NA      NA
## 5      Item 5 0.535    NA   NA      NA
## 6      Item 6 0.245    NA   NA      NA
## 7      Item 7 0.642    NA   NA      NA
## 8      Item 8 0.566    NA   NA      NA
## 9      Item 9 0.416    NA   NA      NA
## 10    Item 10 0.151    NA   NA      NA
## 11    Item 11 0.454    NA   NA      NA
## 12    Item 12 0.355    NA   NA      NA
## 13    Item 13 0.637    NA   NA      NA
## 14    Item 14 0.346    NA   NA      NA
## 15    Item 15 0.418    NA   NA      NA
## 16    Item 16 0.454    NA   NA      NA
```

```
## 17   Item 17 0.694    NA  NA     NA
## 18   Item 18 0.550    NA  NA     NA
## 19      HDRS 0.462    NA  NA     NA
## 20       Age 0.409    NA  NA     NA
## 21       Sex    NA 0.729   0  0.729
## 22 Education 0.609    NA  NA     NA
## 23      Moca 0.647    NA  NA     NA
## 24       FAB 0.662    NA  NA     NA
```

```
errorsControl <- list()
for(i in 1:20) errorsControl[[i]] <- pred_objControl$errors[i,2]
for(i in 21) errorsControl[[i]] <- pred_objControl$errors[i,4]
for(i in 22:24) errorsControl[[i]] <- pred_objControl$errors[i,2]

color_listControl <- list()
for(i in 1:24) color_listControl[[i]] <- "black"
```

Representation of the resulting network with predictability measures

```
Control <- qgraph(net_C$pairwise$wadj,
                    layout = "spring", groups = groups_C,
                    vsize = 6, edge.color = net_C$pairwise$edgecolor,
                    color = col, nodeNames = names_Control,
                    legend.mode = "style2",
                    legend.cex = 0.3, vTrans = 200, vsize = 5.5, esize = 25,
                    pie = errorsControl, pieColor = color_listControl,
                    title = "Control group")
```

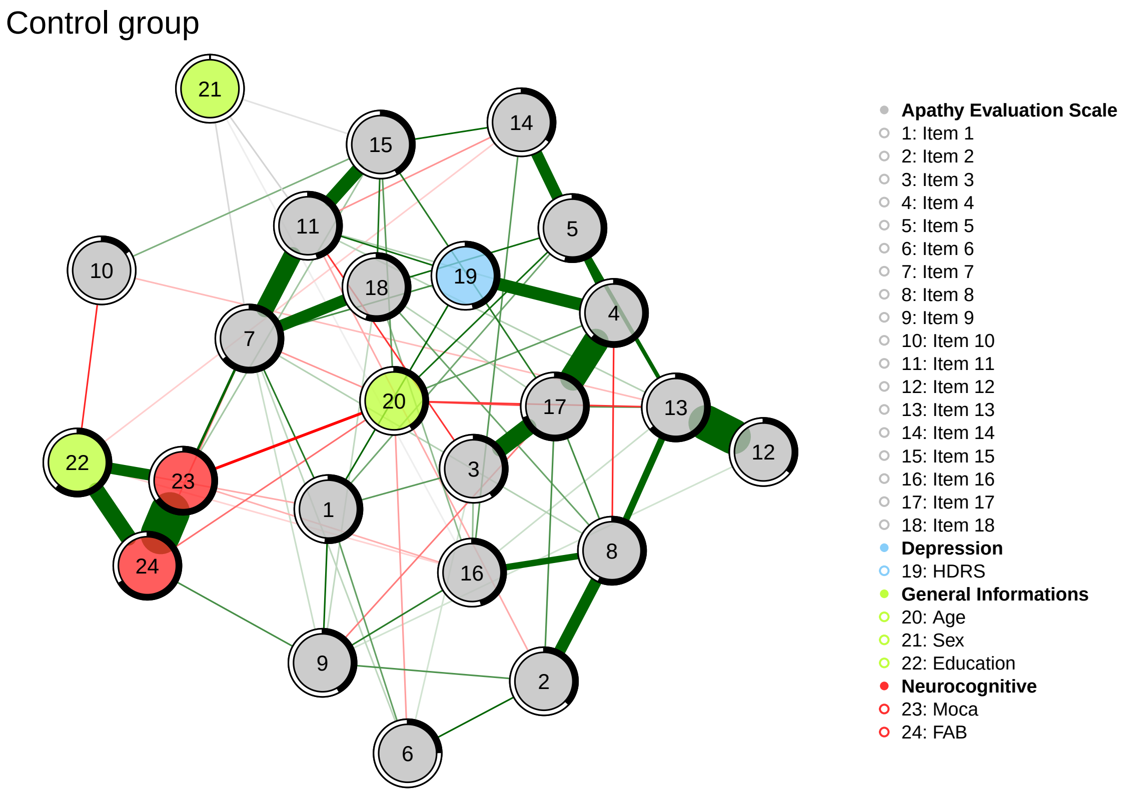

Printing the CONTROL network in a PDF file

```
# pdf("Network_Control.pdf", width = 10)
# qgraph(net_C$pairwise$wadj,
#        layout = "spring", groups = groups_C,
#        vsize = 6, edge.color = net_C$pairwise$edgecolor,
#        color = col, nodeNames = names_Control, legend.mode = "style2",
#        legend.cex = 0.3, vTrans = 200, vsize = 5.5, esize = 25,
#        pie = errorsControl, pieColor = color_listControl,
#        title = "Control group")
# dev.off()
```

Mixed Graphical Model for the MCI Patients

```
set.seed(223)
net_M <- mgm(data = M, alphaSel = "CV", alphaFolds = 10,
             type = c("g","g","g","g","g","g","g","g","g","g","g","g","g",
                      "g","g","g","g","g","g","g","c","g","c","c","c","g","g"),
             level = c("1","1","1","1","1","1","1","1","1","1","1","1","1",
                       "1","1","1","1","1","1","1","2","1","2","2","2","1","1"),
             k = 2, ruleReg = "OR", alphaSeq = 1, scale = TRUE,
             method = "glm", pbar = FALSE)
```

Representation of the MGM

```
qgraph_M <- qgraph(net_M$pairwise$wadj, layout = "spring",
                   edge.color = net_M$pairwise$edgecolor,
                   groups = groups_MCI, color = col,
                   nodeNames = names_MCI, legend.mode = "style2",
                   legend.cex = 0.3, vTrans = 200,
                   vsize = 5.5, esize = 25)
```

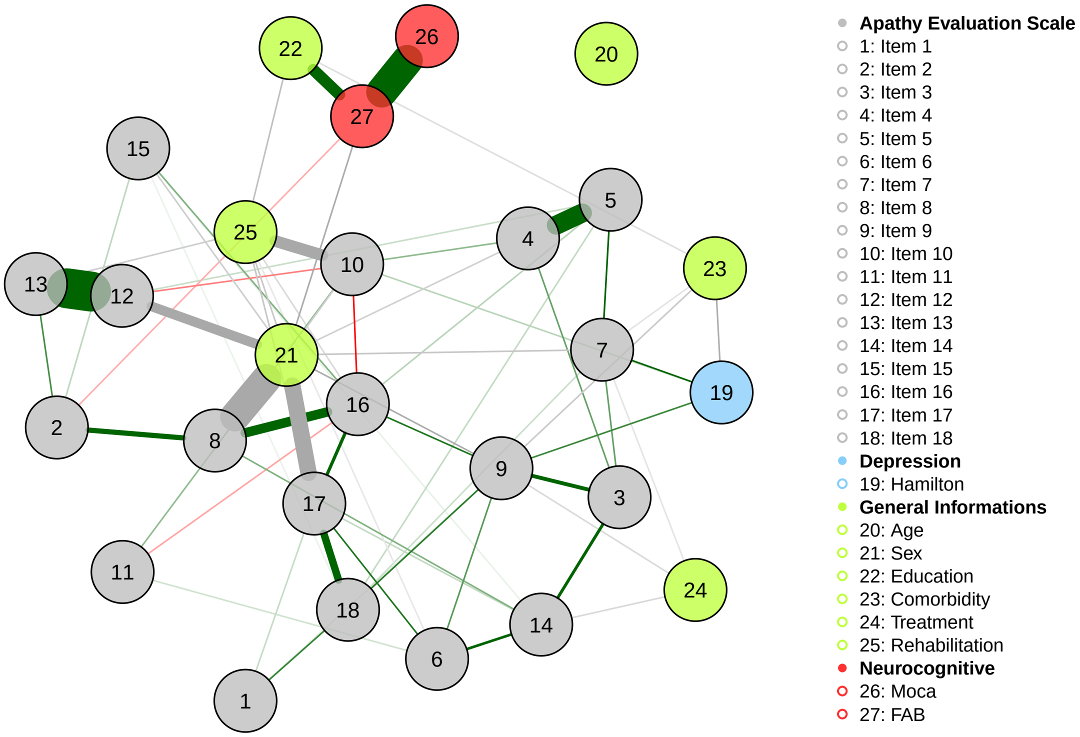

Calculating the Predictability of each node

```r
m <- ncol(M)

pred_objMCI <- predict(object = net_M, data = M,
                       errorCat = c("CC", "nCC", "CCmarg"),
                       errorCon = c("R2"))
pred_objMCI$errors
```

```
##          Variable    R2    CC   nCC CCmarg
## 1          Item 1 0.304    NA    NA     NA
## 2          Item 2 0.419    NA    NA     NA
## 3          Item 3 0.520    NA    NA     NA
## 4          Item 4 0.397    NA    NA     NA
## 5          Item 5 0.501    NA    NA     NA
## 6          Item 6 0.455    NA    NA     NA
## 7          Item 7 0.546    NA    NA     NA
## 8          Item 8 0.493    NA    NA     NA
## 9          Item 9 0.660    NA    NA     NA
## 10        Item 10 0.525    NA    NA     NA
## 11        Item 11 0.000    NA    NA     NA
## 12        Item 12 0.532    NA    NA     NA
## 13        Item 13 0.609    NA    NA     NA
## 14        Item 14 0.533    NA    NA     NA
## 15        Item 15 0.107    NA    NA     NA
## 16        Item 16 0.582    NA    NA     NA
## 17        Item 17 0.667    NA    NA     NA
## 18        Item 18 0.583    NA    NA     NA
## 19       Hamilton 0.474    NA    NA     NA
## 20            Age 0.000    NA    NA     NA
## 21            Sex    NA 0.896 0.652  0.701
## 22      Education 0.371    NA    NA     NA
## 23    Comorbidity    NA 0.753 0.000  0.753
## 24      Treatment    NA 0.662 0.000  0.662
## 25 Rehabilitation    NA 0.688 0.250  0.584
## 26           Moca 0.483    NA    NA     NA
## 27            FAB 0.570    NA    NA     NA
```

```r
errorsMCI <- list()
for(i in 1:20) errorsMCI[[i]] <- pred_objMCI$errors[i,2]
for(i in 21) errorsMCI[[i]] <- pred_objMCI$errors[i,3]
for(i in 22) errorsMCI[[i]] <- pred_objMCI$errors[i,2]
for(i in 23:25) errorsMCI[[i]] <- pred_objMCI$errors[i,3]
for(i in 26:27) errorsMCI[[i]] <- pred_objMCI$errors[i,2]

color_listMCI <- list()
for(i in 1:27) color_listMCI[[i]] <- "black"
```

Representation of the resulting network with predictability measures

```r
MCI <- qgraph(net_M$pairwise$wadj,
              layout = "spring", groups = groups_MCI,
              vsize = 6, edge.color = net_M$pairwise$edgecolor,
              color = col, nodeNames = names_MCI, legend.mode = "style2",
```

```
legend.cex = 0.3, vTrans = 200, vsize = 5.5, esize = 25,
pie = errorsMCI, pieColor = color_listMCI,
title = "Mild Cognitive Impairment group")
```

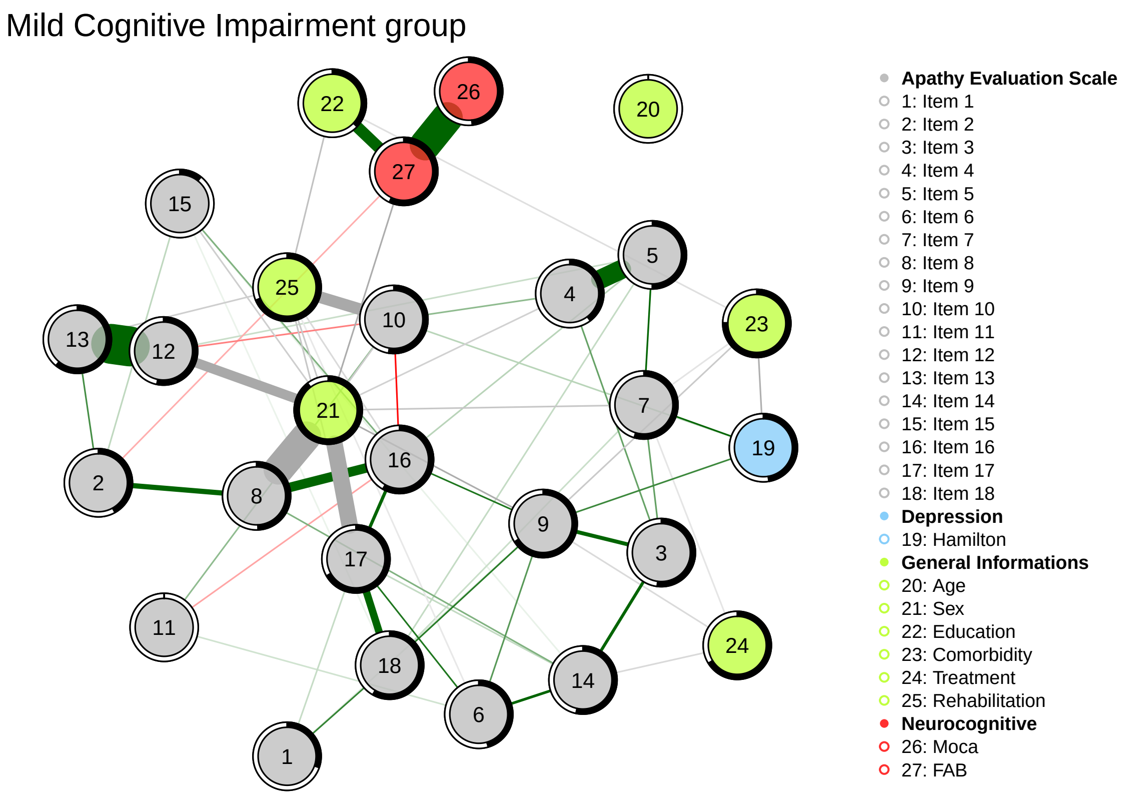

Printing the PDF of MCI

```
# pdf("Network_MCI.pdf", width = 10)
# qgraph(net_M$pairwise$wadj,
#        layout = "spring", groups = groups,
#        vsize = 6, edge.color = net_M$pairwise$edgecolor,
#        color = col, nodeNames = names_MCI, legend.mode = "style2",
#        legend.cex = 0.5, vTrans = 200, vsize = 5.5, esize = 25,
#        pie = errorsMCI, pieColor = color_listMCI,
#        title = "Mild Cognitive Impairment group")
# dev.off()
```

Mixed Graphical Model for the ALZHEIMER Patients

```
set.seed(333)
net_A <- mgm(data = A, alphaSel = "CV", alphaFolds = 10,
             type = c("g","g","g","g","g","g","g","g","g","g","g","g","g",
                      "g","g","g","g","g","g","g","c","g","c","c","g","g"),
             level = c("1","1","1","1","1","1","1","1","1","1","1","1","1",
                       "1","1","1","1","1","1","1","2","1","2","2","1","1"),
              k = 2, ruleReg = "OR", alphaSeq = 1, scale = TRUE,
              method = "glm", pbar = FALSE)
```

Representation of the MGM

```
qgraph_A <- qgraph(net_A$pairwise$wadj, layout = "spring",
                   edge.color = net_A$pairwise$edgecolor,
                   groups = groups_A, color = col,
                   nodeNames = colnames(A), legend.mode = "style2",
                   legend.cex = 0.3, vTrans = 200,
                   vsize = 5.5, esize = 25)
```

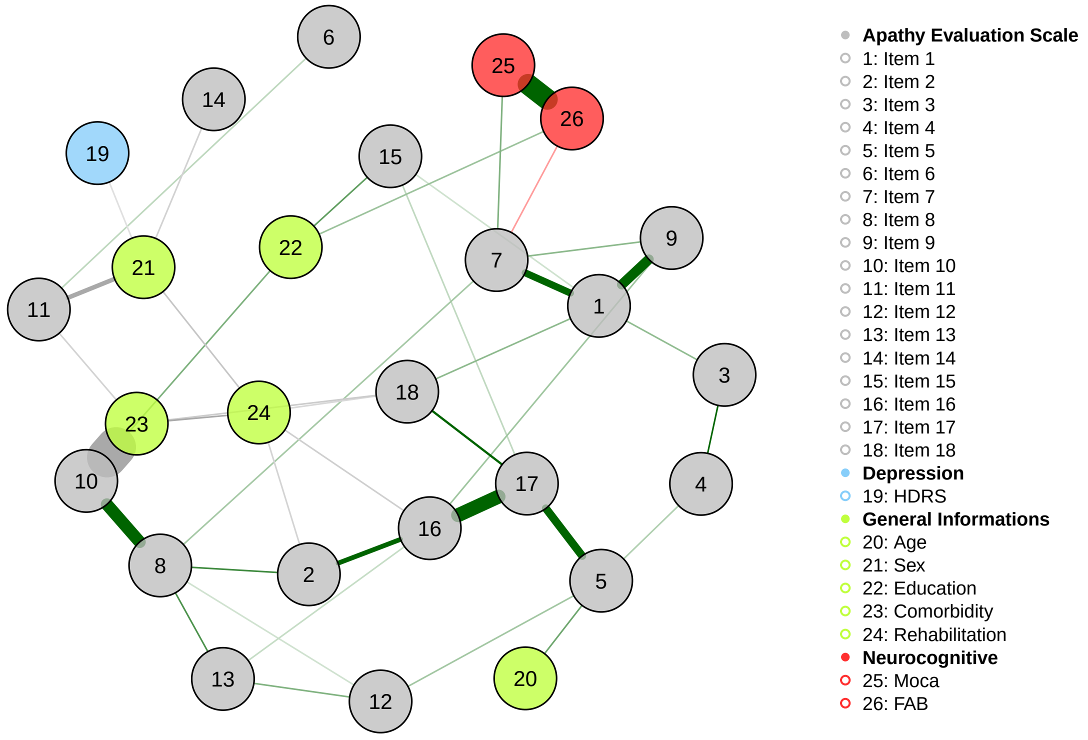

Calculating the Predictability of each node

```r
a <- ncol(A)

pred_objAlz <- predict(object = net_A, data = A,
                       errorCat = c("CC", "nCC", "CCmarg"),
                       errorCon = c("R2"))
pred_objAlz$errors
```

```
##          Variable    R2    CC   nCC CCmarg
## 1          Item 1 0.798    NA    NA     NA
## 2          Item 2 0.573    NA    NA     NA
## 3          Item 3 0.463    NA    NA     NA
## 4          Item 4 0.449    NA    NA     NA
## 5          Item 5 0.574    NA    NA     NA
## 6          Item 6 0.000    NA    NA     NA
## 7          Item 7 0.793    NA    NA     NA
## 8          Item 8 0.700    NA    NA     NA
## 9          Item 9 0.683    NA    NA     NA
## 10        Item 10 0.788    NA    NA     NA
## 11        Item 11 0.227    NA    NA     NA
## 12        Item 12 0.297    NA    NA     NA
## 13        Item 13 0.322    NA    NA     NA
## 14        Item 14 0.000    NA    NA     NA
## 15        Item 15 0.353    NA    NA     NA
## 16        Item 16 0.817    NA    NA     NA
## 17        Item 17 0.762    NA    NA     NA
## 18        Item 18 0.792    NA    NA     NA
## 19           HDRS 0.000    NA    NA     NA
## 20            Age 0.063    NA    NA     NA
## 21            Sex    NA 0.833 0.545  0.633
## 22      Education 0.387    NA    NA     NA
## 23    Comorbidity    NA 0.900 0.667  0.700
## 24 Rehabilitation    NA 0.733 0.000  0.733
## 25           Moca 0.480    NA    NA     NA
## 26            FAB 0.454    NA    NA     NA
```

```r
errorsAlz <- list()
for(i in 1:20) errorsAlz[[i]] <- pred_objAlz$errors[i,2]
for(i in 21) errorsAlz[[i]] <- pred_objAlz$errors[i,3]
for(i in 22) errorsAlz[[i]] <- pred_objAlz$errors[i,2]
for(i in 23:24) errorsAlz[[i]] <- pred_objAlz$errors[i,3]
for(i in 25:26) errorsAlz[[i]] <- pred_objAlz$errors[i,2]

color_listAlz <- list()
for(i in 1:26) color_listAlz[[i]] <- "black"
```

Representation of the resulting network with predictability measures

```r
Alzheimer <- qgraph(net_A$pairwise$wadj,
              layout = "spring", groups = groups_A,
              vsize = 6, edge.color = net_A$pairwise$edgecolor,
              color = col, nodeNames = colnames(A), legend.mode = "style2",
              legend.cex = 0.3, vTrans = 200, vsize = 5.5, esize = 25,
```

```r
            pie = errorsAlz, pieColor = color_listAlz,
            title = "Alzheimer patients group")
```

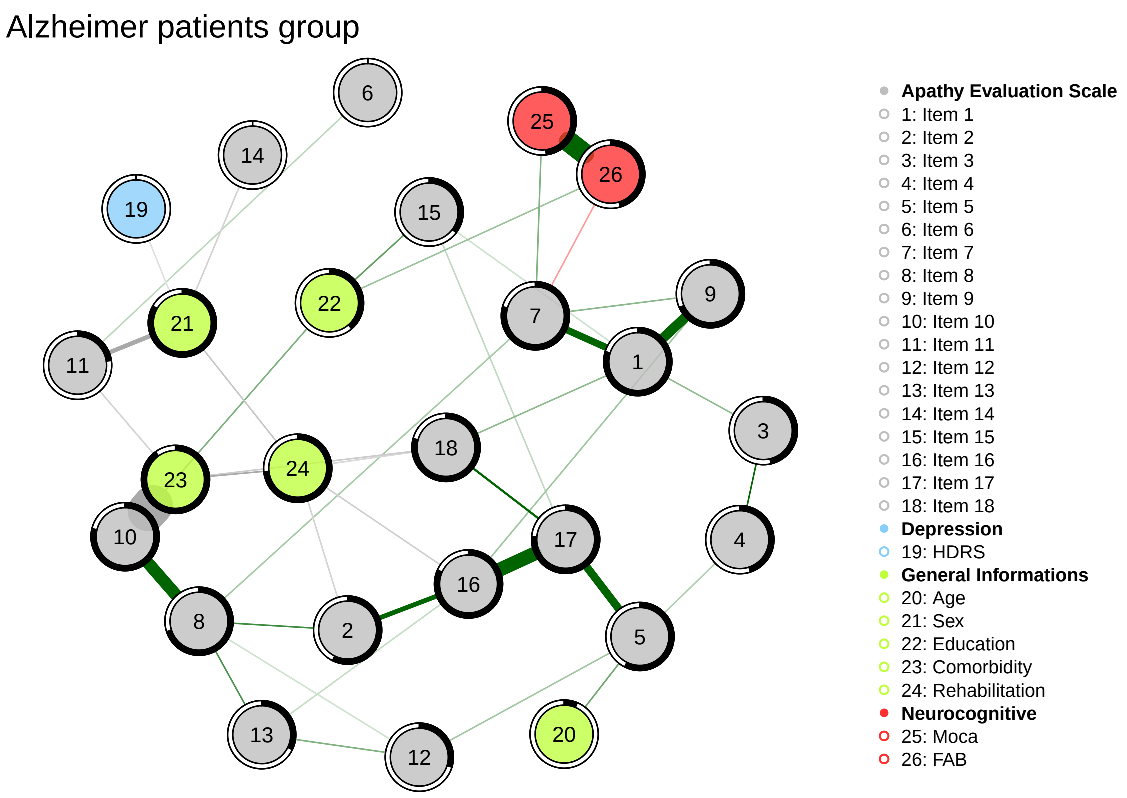

Printing the PDF of Alzheimer Patients

```r
# pdf("Network_Alzh.pdf", width = 10)
# qgraph(net_A$pairwise$wadj,
#        layout = "spring", groups = groups_A,
#        vsize = 6, edge.color = net_A$pairwise$edgecolor,
#        color = col, nodeNames = colnames(A), legend.mode = "style2",
#        legend.cex = 0.5, vTrans = 200, vsize = 5.5, esize = 25,
#        pie = errorsAlz, pieColor = color_listAlz,
#        title = "Alzheimer patients group")
# dev.off()
```

CENTRALITY MEASURE FOR THE THREE NETWORKS and their relative BOOTSTRAPS

Centrality measure in CONTROL Patients

```
?centralityPlot
centralityPlot(Control, include = c("Betweenness", "Closeness","ExpectedInfluence"),
               scale = "z-scores", theme_bw = FALSE,
               weighted = TRUE, labels = colnames(C), orderBy = "ExpectedInfluence")
```

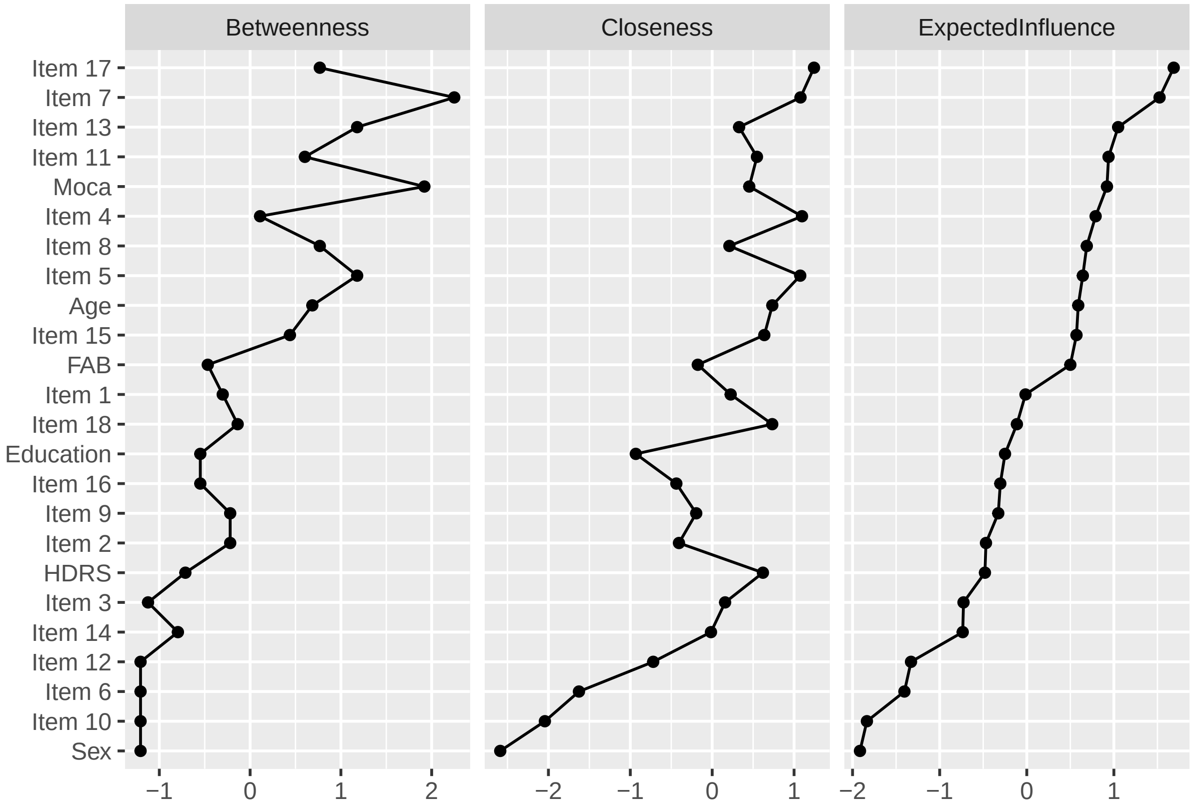


```
# set.seed(444)
# fit_ControlBoot   <- bootnet(C, nBoots    = 500,  nCores  = 16, default = "mgm",
#                             type = "case", statistics = c("strength",
#                                                           "betweenness",
#                                                           "closeness",
#                                                           "ExpectedInfluence"))
# Bootstrap of Controls dataset
plot(fit_ControlBoot, statistics = c("strength", "betweenness", "ExpectedInfluence"))
```

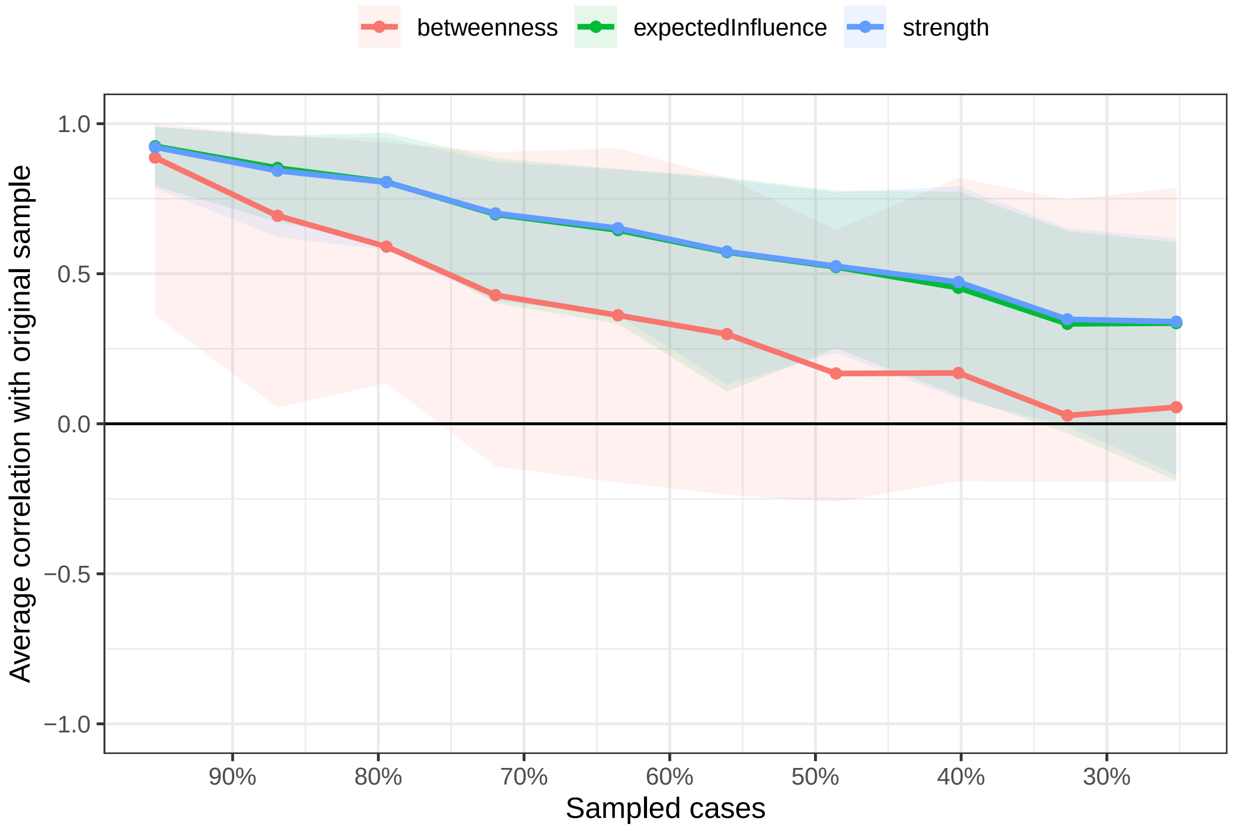


```r
corStability(fit_ControlBoot)
```

```
## === Correlation Stability Analysis ===
##
## Sampling levels tested:
##    nPerson Drop%  n
## 1       27  74.8 51
## 2       35  67.3 59
## 3       43  59.8 49
## 4       52  51.4 50
## 5       60  43.9 53
## 6       68  36.4 55
## 7       77  28.0 52
## 8       85  20.6 35
## 9       93  13.1 54
## 10     102   4.7 42
##
## Maximum drop proportions to retain correlation of 0.7 in at least 95% of the samples:
##
## betweenness: 0.047 (CS-coefficient is lowest level tested)
##   - For more accuracy, run bootnet(..., caseMin = 0, caseMax = 0.131)
##
## closeness: 0
##   - For more accuracy, run bootnet(..., caseMin = 0, caseMax = 0.047)
##
```

```
## edge: 0.206
##   - For more accuracy, run bootnet(..., caseMin = 0.131, caseMax = 0.28)
##
## expectedInfluence: 0.131
##   - For more accuracy, run bootnet(..., caseMin = 0.047, caseMax = 0.206)
##
## strength: 0.047 (CS-coefficient is lowest level tested)
##   - For more accuracy, run bootnet(..., caseMin = 0, caseMax = 0.131)
##
## Accuracy can also be increased by increasing both 'nBoots' and 'caseN'.
```

Centrality measure in MCI Patients

```
centralityPlot(MCI, include = c("Betweenness","Closeness","ExpectedInfluence"),
               scale = "z-score", theme_bw = FALSE,
               weighted = TRUE, labels = colnames(M), orderBy = "ExpectedInfluence")
```

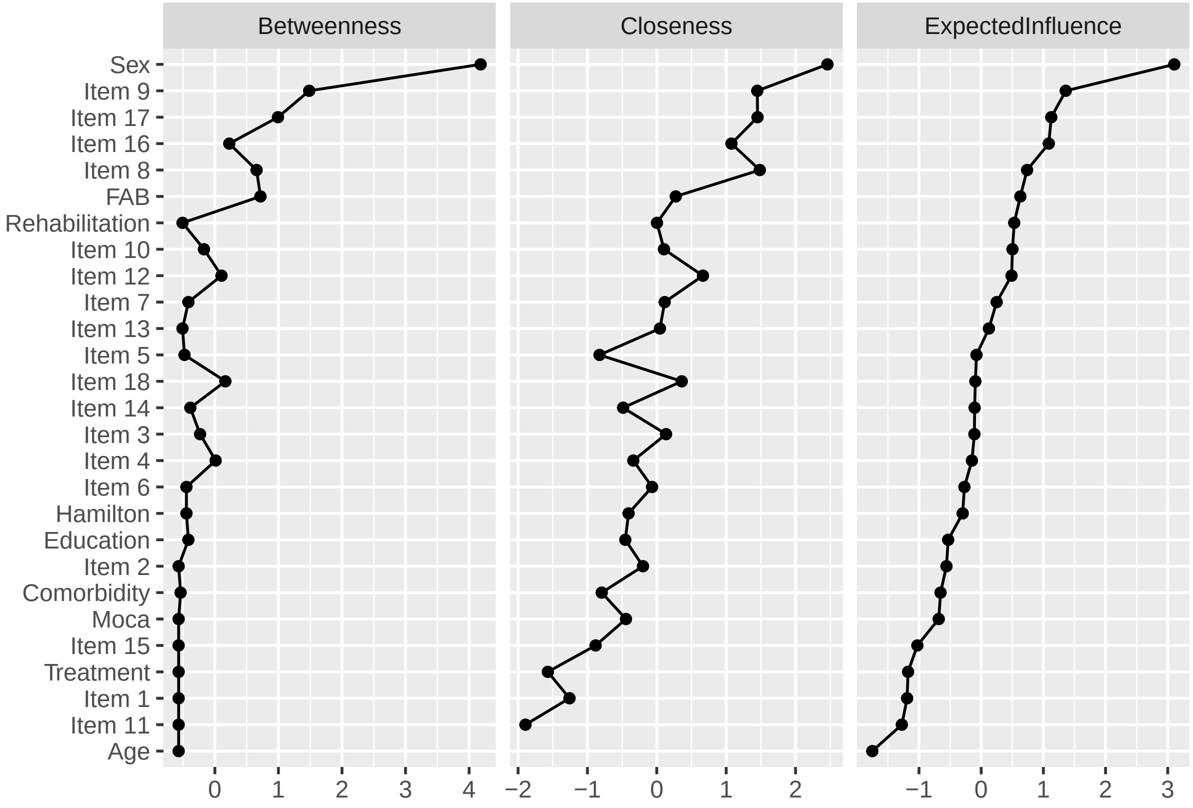


```
# set.seed(555)
# fit_MCIBoot   <- bootnet(M,   nBoots = 500,   nCores = 8, default = "mgm",
#                         type   = "case", statistics = c("strength",
#                                                          "betweenness",
#                                                          "closeness",
#                                                          "ExpectedInfluence"))
# Bootstrap of MCI dataset
plot(fit_MCIBoot, statistics = c("strength", "betweenness", "ExpectedInfluence"))
```

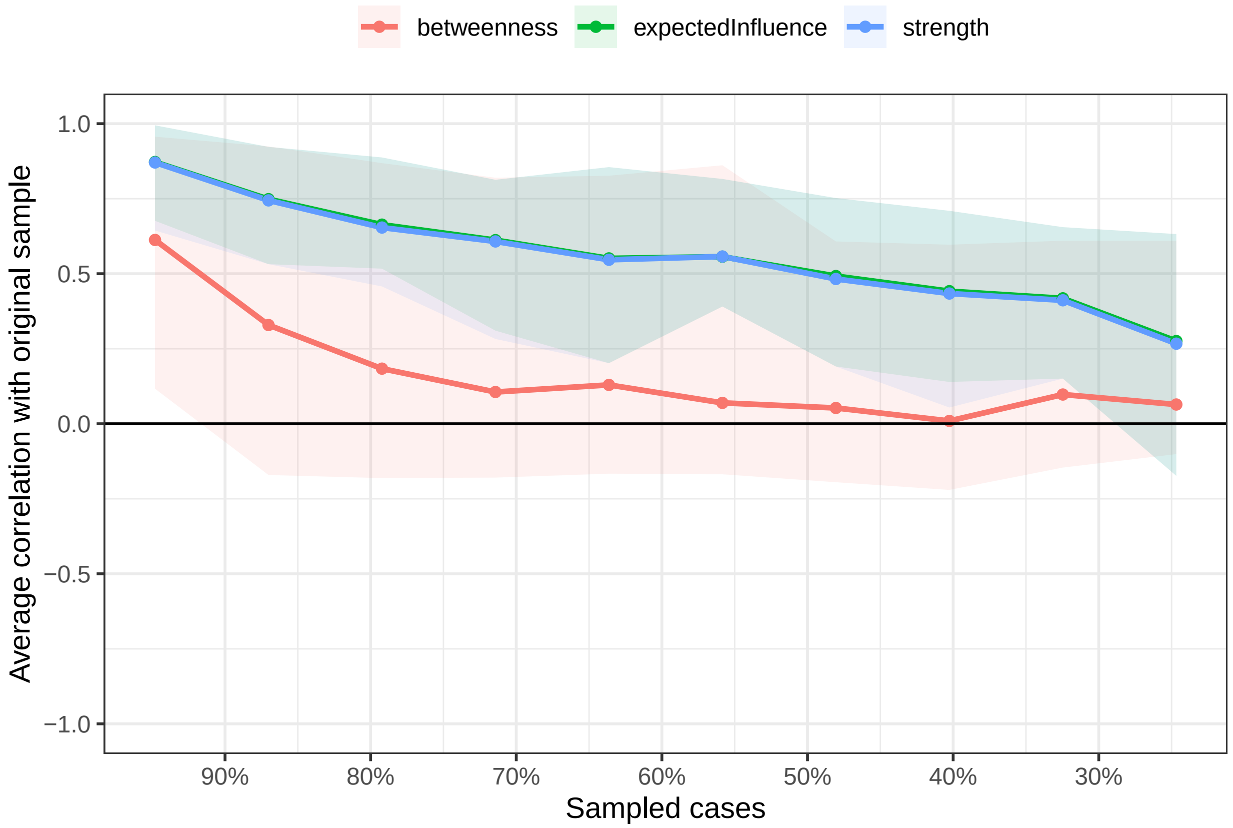


```
corStability(fit_MCIBoot)
```

```
## === Correlation Stability Analysis ===
##
## Sampling levels tested:
##    nPerson Drop%  n
## 1       19  75.3 55
## 2       25  67.5 49
## 3       31  59.7 60
## 4       37  51.9 52
## 5       43  44.2 32
## 6       49  36.4 47
## 7       55  28.6 58
## 8       61  20.8 44
## 9       67  13.0 55
## 10      73   5.2 48
##
## Maximum drop proportions to retain correlation of 0.7 in at least 95% of the samples:
##
## betweenness: 0
##   - For more accuracy, run bootnet(..., caseMin = 0, caseMax = 0.052)
##
## closeness: 0
##   - For more accuracy, run bootnet(..., caseMin = 0, caseMax = 0.052)
##
```

```
## edge: 0.208
##   - For more accuracy, run bootnet(..., caseMin = 0.13, caseMax = 0.286)
##
## expectedInfluence: 0
##   - For more accuracy, run bootnet(..., caseMin = 0, caseMax = 0.052)
##
## strength: 0
##   - For more accuracy, run bootnet(..., caseMin = 0, caseMax = 0.052)
##
## Accuracy can also be increased by increasing both 'nBoots' and 'caseN'.
```

Centrality measure in ALZHEIMER Patients

```r
centralityPlot(Alzheimer, include = c("Betweenness","Closeness","ExpectedInfluence"),
               scale = "z-scores", theme_bw = FALSE,
               weighted = TRUE, labels = colnames(A), orderBy = "ExpectedInfluence")
```

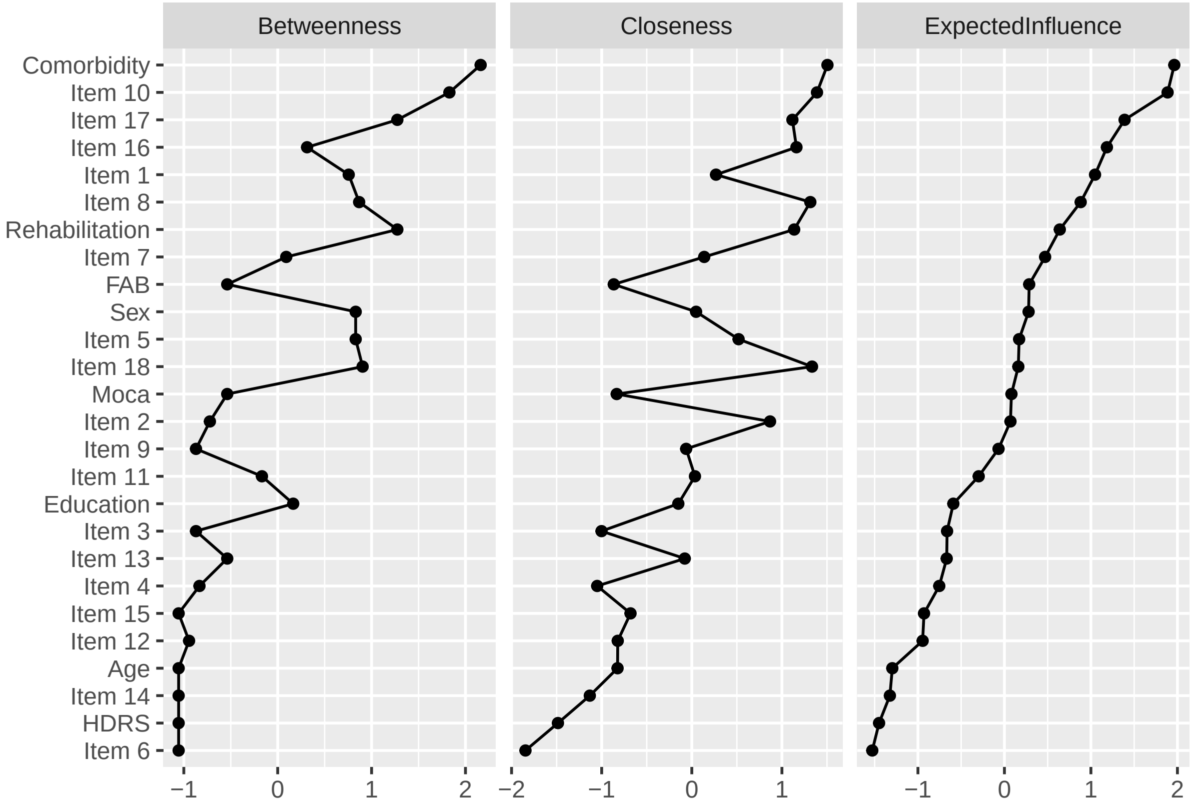


```r
# set.seed(555)
# fit_AlzhBoot  <- bootnet(A, nBoots    = 500,  nCores  = 8, default = "mgm",
#                          type = "case", statistics = c("strength",
#                                                        "betweenness",
#                                                        "closeness",
#                                                        "ExpectedInfluence"))
# Bootstrap of Alzheimer dataset
plot(fit_AlzhBoot, statistics = c("strength", "betweenness", "ExpectedInfluence"))
```

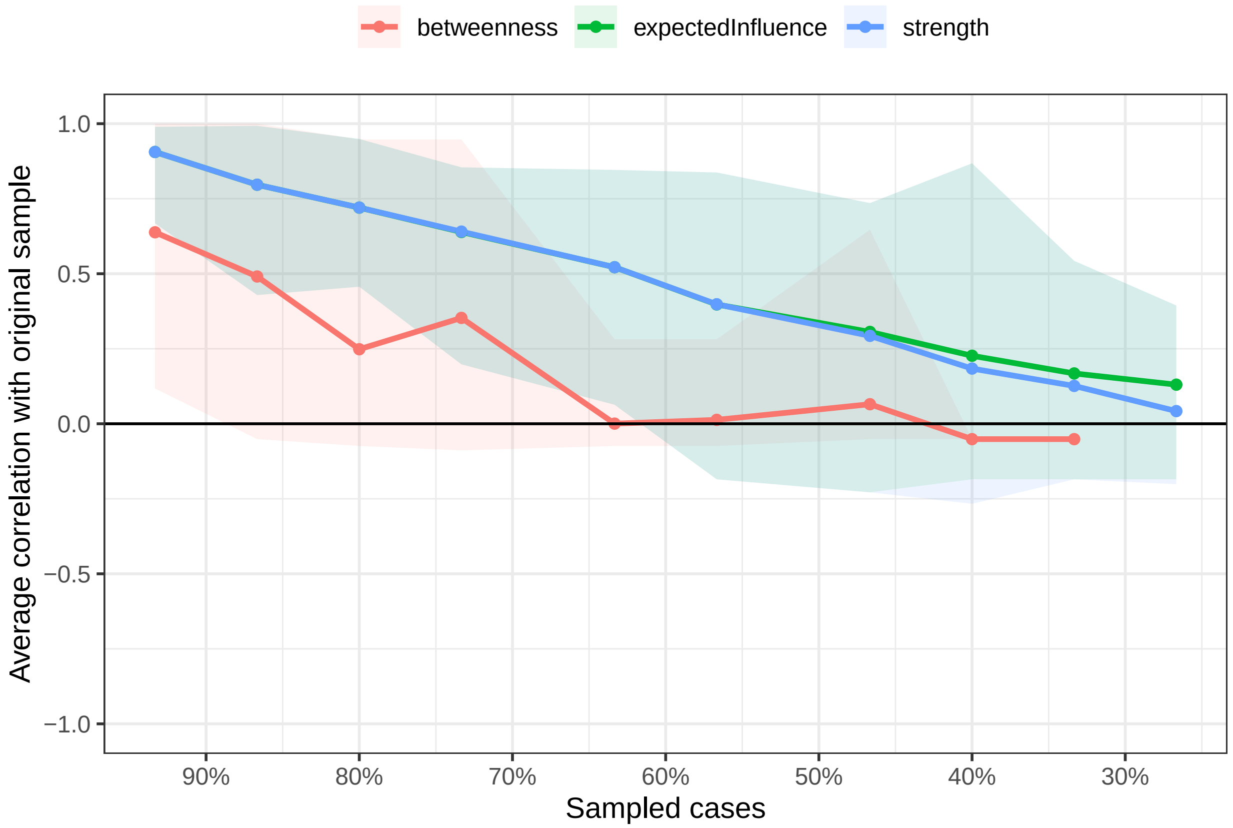


```r
corStability(fit_AlzhBoot)
```

```
## === Correlation Stability Analysis ===
##
## Sampling levels tested:
##    nPerson Drop%  n
## 1        8  73.3 29
## 2       10  66.7 46
## 3       12  60.0 51
## 4       14  53.3 65
## 5       17  43.3 55
## 6       19  36.7 49
## 7       22  26.7 48
## 8       24  20.0 58
## 9       26  13.3 44
## 10      28   6.7 55
##
## Maximum drop proportions to retain correlation of 0.7 in at least 95% of the samples:
##
## betweenness: 0
##   - For more accuracy, run bootnet(..., caseMin = 0, caseMax = 0.067)
##
## closeness: 0
##   - For more accuracy, run bootnet(..., caseMin = 0, caseMax = 0.067)
##
```

```
## edge: 0.067 (CS-coefficient is lowest level tested)
##   - For more accuracy, run bootnet(..., caseMin = 0, caseMax = 0.133)
##
## expectedInfluence: 0.067 (CS-coefficient is lowest level tested)
##   - For more accuracy, run bootnet(..., caseMin = 0, caseMax = 0.133)
##
## strength: 0.067 (CS-coefficient is lowest level tested)
##   - For more accuracy, run bootnet(..., caseMin = 0, caseMax = 0.133)
##
## Accuracy can also be increased by increasing both 'nBoots' and 'caseN'.
```

Analysis with igraph

Extracting the layout coordinates for each node in order to maintain it in the igraph representation

```
Layout_Control <- Control$layout
Layout_MCI <- MCI$layout
Layout_Alzh <- Alzheimer$layout
```

Transforming the networks in igraph objects to conduct cluster analysis

```
library(igraph)

igraph_Control <- as.igraph(Control)
igraph_Alzh <- as.igraph(Alzheimer)
igraph_MCI  <- as.igraph(MCI)
```

Network Density for each group

```
ecount(igraph_Control)
```

```
## [1] 77
```

```
edge_density(igraph_Control, loops = FALSE) # Control group
```

```
## [1] 0.2789855
```

```
ecount(igraph_MCI)
```

```
## [1] 65
```

```
edge_density(igraph_MCI, loops = FALSE) # MCI group
```

```
## [1] 0.1851852
```

```
ecount(igraph_Alzh)
```

```
## [1] 41
```

```
edge_density(igraph_Alzh, loops = FALSE) # Alzheimer group
```

```
## [1] 0.1261538
```

Cluster analysis with WALKTRAP algorithm for the Networks

Clusters for CONTROL group

```r
noClusterControl <- 0
set.seed(666)
for (i in 1:1000){
  communityControl <- cluster_walktrap(igraph_Control,
                                       weights = E(igraph_Control)$weight,
                                       steps = i,
                                       merges = TRUE,
                                       modularity = TRUE,
                                       membership = TRUE)
  noClusterControl[i] <- length(communityControl)
}

plot(noClusterControl)
```

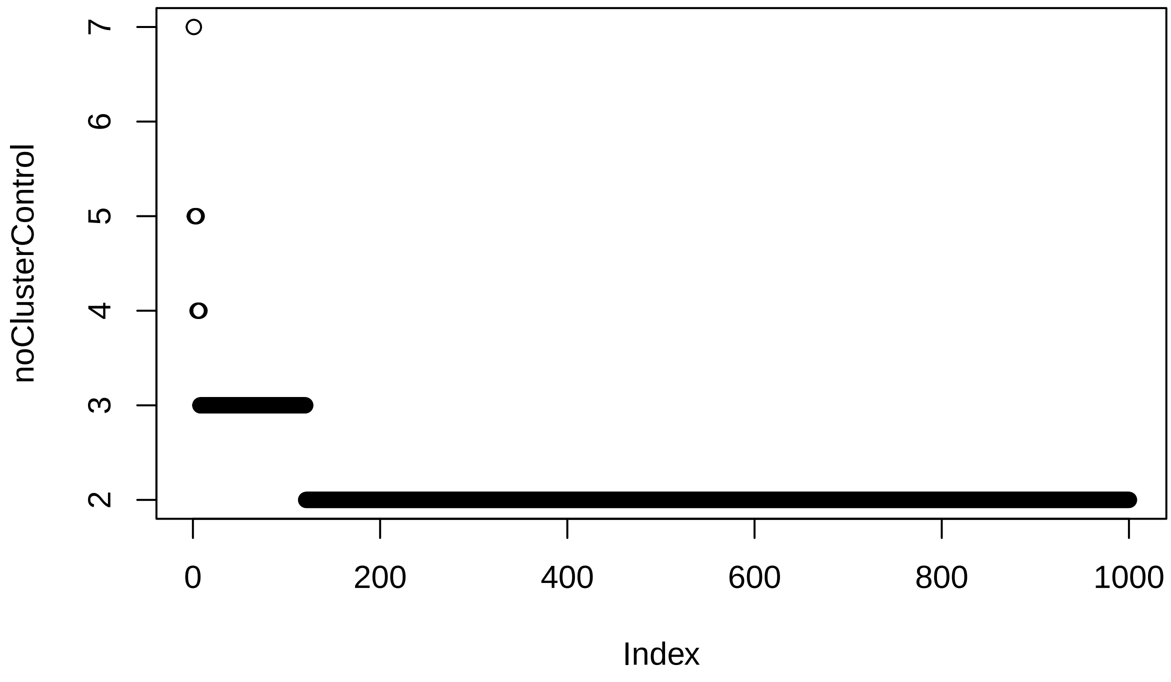


```r
median(noClusterControl)
```

```
## [1] 2
```

```r
mean(noClusterControl)
```

```
## [1] 2.133
```

```r
# noClusterControl

communityControlPlot <- cluster_walktrap(igraph_Control,
                                         weights = E(igraph_Control)$weight,
                                         steps = 300,
                                         merges = TRUE,
                                         modularity = TRUE,
                                         membership = TRUE)

plot(communityControlPlot, igraph_Control, layout = Layout_Control)
```

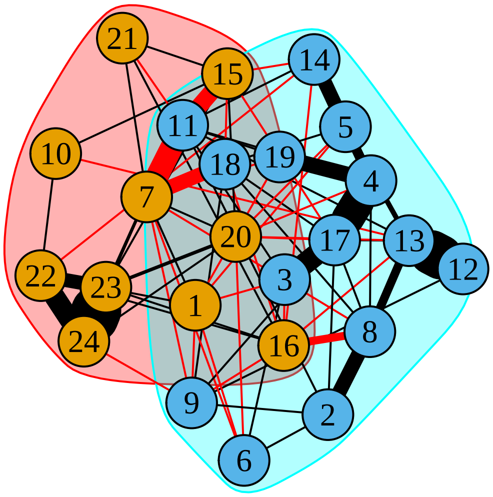

Clusters for MCI group

```r
noClusterMCI <- 0
set.seed(777)
for (i in 1:1000){
  communityMCI <- cluster_walktrap(igraph_MCI,
                                    weights = E(igraph_MCI)$weight,
                                    steps = i,
                                    merges = TRUE,
                                    modularity = TRUE,
                                    membership = TRUE)
  noClusterMCI[i] <- length(communityMCI)
}

plot(noClusterMCI)
```

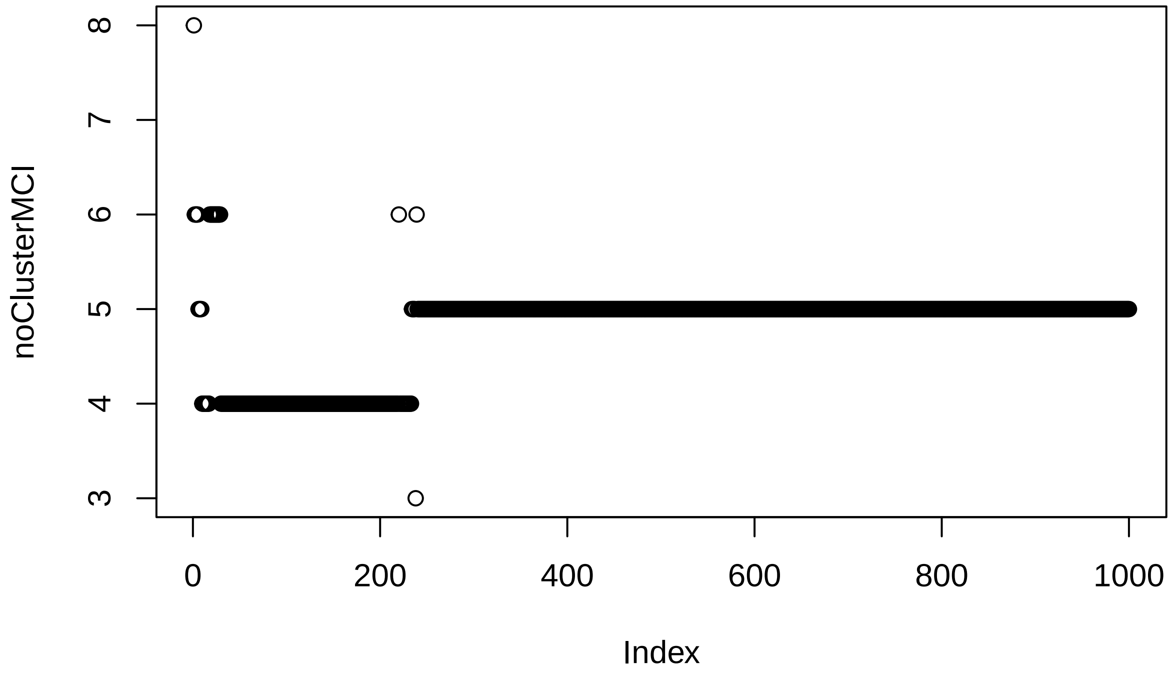


```
median(noClusterMCI)
```

```
## [1] 5
```

```
mean(noClusterMCI)
```

```
## [1] 4.808
```

```
# noClusterMCI

communityMCIPlot <- cluster_walktrap(igraph_MCI,
                                     weights = E(igraph_MCI)$weight,
                                     steps = 200,
                                     merges = TRUE,
                                     modularity = TRUE,
                                     membership = TRUE)

plot(communityMCIPlot, igraph_MCI, layout = Layout_MCI)
```

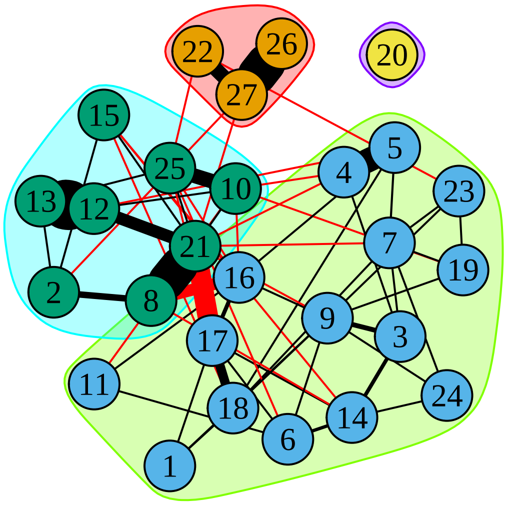

Clusters for Alzheimer group

```
noClusterAlz <- 0
set.seed(888)
for (i in 1:1000){
  communityAlzheimer <- cluster_walktrap(igraph_Alzh,
                                          weights = E(igraph_Alzh)$weight,
                                          steps = i,
                                          merges = TRUE,
                                          modularity = TRUE,
                                          membership = TRUE)
  noClusterAlz[i] <- length(communityAlzheimer)
}

plot(noClusterAlz)
```

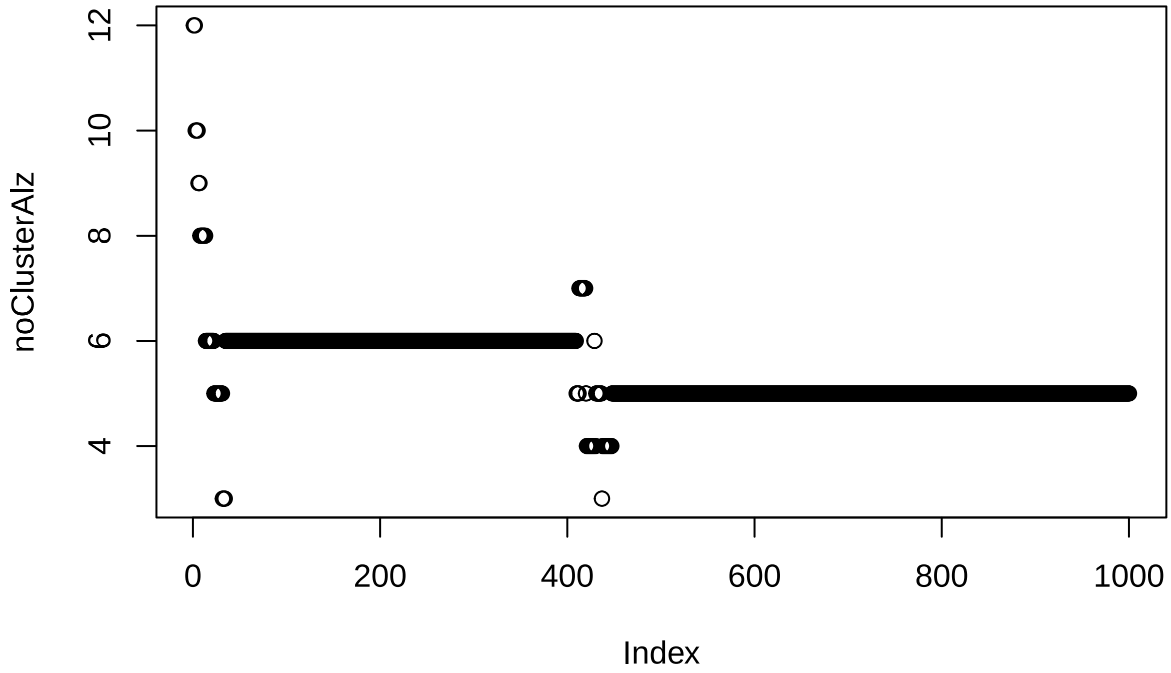


```r
median(noClusterAlz)
```

```
## [1] 5
```

```r
mean(noClusterAlz)
```

```
## [1] 5.427
```

```r
# noClusterAlz

communityAlzheimerPlot <- cluster_walktrap(igraph_Alzh,
                                           weights = E(igraph_Alzh)$weight,
                                           steps = 200,
                                           merges = TRUE,
                                           modularity = TRUE,
                                           membership = TRUE)


plot(communityAlzheimerPlot, igraph_Alzh, layout = Layout_Alzh)
```

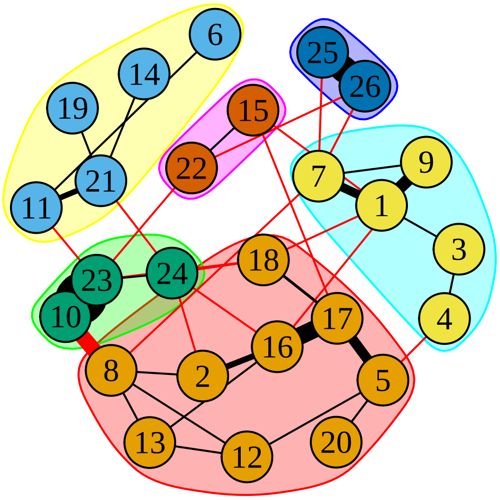

Make the PDF with the clusters of the 3 Groups

```
# pdf("Clusters.pdf", width = 20)
# par(mfrow = c(1,3))
# plot(communityControlPlot, igraph_Control, layout = Layout_Control)
# plot(communityMCIPlot, igraph_MCI, layout = Layout_MCI)
# plot(communityAlzheimerPlot, igraph_Alzh, layout = Layout_Alzh)
# dev.off()
```

Centrality tables

```
cenControls <- centralityTable(Control, standardized = FALSE, relative = TRUE)
cenMCI <- centralityTable(MCI, standardized = FALSE, relative = TRUE)
cenAlzheimer <- centralityTable(Alzheimer, standardized = FALSE,
                                relative = TRUE)
cenControls
```

```
##      graph type node          measure      value
## 1  graph 1   NA    1      Betweenness 0.26190476
## 2  graph 1   NA    2      Betweenness 0.28571429
## 3  graph 1   NA    3      Betweenness 0.02380952
## 4  graph 1   NA    4      Betweenness 0.38095238
## 5  graph 1   NA    5      Betweenness 0.69047619
## 6  graph 1   NA    6      Betweenness 0.00000000
## 7  graph 1   NA    7      Betweenness 1.00000000
## 8  graph 1   NA    8      Betweenness 0.57142857
```

```
## 9  graph 1   NA    9       Betweenness 0.28571429
## 10 graph 1   NA   10       Betweenness 0.00000000
## 11 graph 1   NA   11       Betweenness 0.52380952
## 12 graph 1   NA   12       Betweenness 0.00000000
## 13 graph 1   NA   13       Betweenness 0.69047619
## 14 graph 1   NA   14       Betweenness 0.11904762
## 15 graph 1   NA   15       Betweenness 0.47619048
## 16 graph 1   NA   16       Betweenness 0.19047619
## 17 graph 1   NA   17       Betweenness 0.57142857
## 18 graph 1   NA   18       Betweenness 0.30952381
## 19 graph 1   NA   19       Betweenness 0.14285714
## 20 graph 1   NA   20       Betweenness 0.54761905
## 21 graph 1   NA   21       Betweenness 0.00000000
## 22 graph 1   NA   22       Betweenness 0.19047619
## 23 graph 1   NA   23       Betweenness 0.90476190
## 24 graph 1   NA   24       Betweenness 0.21428571
## 25 graph 1   NA    1         Closeness 0.86840959
## 26 graph 1   NA    2         Closeness 0.78674953
## 27 graph 1   NA    3         Closeness 0.85962116
## 28 graph 1   NA    4         Closeness 0.98108467
## 29 graph 1   NA    5         Closeness 0.97832264
## 30 graph 1   NA    6         Closeness 0.62879580
## 31 graph 1   NA    7         Closeness 0.97872520
## 32 graph 1   NA    8         Closeness 0.86640067
## 33 graph 1   NA    9         Closeness 0.81400380
## 34 graph 1   NA   10         Closeness 0.57495310
## 35 graph 1   NA   11         Closeness 0.90990398
## 36 graph 1   NA   12         Closeness 0.74604834
## 37 graph 1   NA   13         Closeness 0.88174370
## 38 graph 1   NA   14         Closeness 0.83730416
## 39 graph 1   NA   15         Closeness 0.92155236
## 40 graph 1   NA   16         Closeness 0.78263227
## 41 graph 1   NA   17         Closeness 1.00000000
## 42 graph 1   NA   18         Closeness 0.93405589
## 43 graph 1   NA   19         Closeness 0.91934987
## 44 graph 1   NA   20         Closeness 0.93420072
## 45 graph 1   NA   21         Closeness 0.50448391
## 46 graph 1   NA   22         Closeness 0.71859126
## 47 graph 1   NA   23         Closeness 0.89778905
## 48 graph 1   NA   24         Closeness 0.81650688
## 49 graph 1   NA    1          Strength 0.61382422
## 50 graph 1   NA    2          Strength 0.51080623
## 51 graph 1   NA    3          Strength 0.45233053
## 52 graph 1   NA    4          Strength 0.79653808
## 53 graph 1   NA    5          Strength 0.76315574
## 54 graph 1   NA    6          Strength 0.29832743
## 55 graph 1   NA    7          Strength 0.96319565
## 56 graph 1   NA    8          Strength 0.77353262
## 57 graph 1   NA    9          Strength 0.54291360
## 58 graph 1   NA   10          Strength 0.20056184
## 59 graph 1   NA   11          Strength 0.83027054
## 60 graph 1   NA   12          Strength 0.31549889
## 61 graph 1   NA   13          Strength 0.85523357
## 62 graph 1   NA   14          Strength 0.45021451
```

```
## 63 graph 1   NA   15          Strength 0.74671160
## 64 graph 1   NA   16          Strength 0.54817021
## 65 graph 1   NA   17          Strength 1.00000000
## 66 graph 1   NA   18          Strength 0.59142673
## 67 graph 1   NA   19          Strength 0.50811232
## 68 graph 1   NA   20          Strength 0.75127145
## 69 graph 1   NA   21          Strength 0.18249122
## 70 graph 1   NA   22          Strength 0.56049268
## 71 graph 1   NA   23          Strength 0.82608238
## 72 graph 1   NA   24          Strength 0.73063013
## 73 graph 1   NA    1 ExpectedInfluence 0.61382422
## 74 graph 1   NA    2 ExpectedInfluence 0.51080623
## 75 graph 1   NA    3 ExpectedInfluence 0.45233053
## 76 graph 1   NA    4 ExpectedInfluence 0.79653808
## 77 graph 1   NA    5 ExpectedInfluence 0.76315574
## 78 graph 1   NA    6 ExpectedInfluence 0.29832743
## 79 graph 1   NA    7 ExpectedInfluence 0.96319565
## 80 graph 1   NA    8 ExpectedInfluence 0.77353262
## 81 graph 1   NA    9 ExpectedInfluence 0.54291360
## 82 graph 1   NA   10 ExpectedInfluence 0.20056184
## 83 graph 1   NA   11 ExpectedInfluence 0.83027054
## 84 graph 1   NA   12 ExpectedInfluence 0.31549889
## 85 graph 1   NA   13 ExpectedInfluence 0.85523357
## 86 graph 1   NA   14 ExpectedInfluence 0.45021451
## 87 graph 1   NA   15 ExpectedInfluence 0.74671160
## 88 graph 1   NA   16 ExpectedInfluence 0.54817021
## 89 graph 1   NA   17 ExpectedInfluence 1.00000000
## 90 graph 1   NA   18 ExpectedInfluence 0.59142673
## 91 graph 1   NA   19 ExpectedInfluence 0.50811232
## 92 graph 1   NA   20 ExpectedInfluence 0.75127145
## 93 graph 1   NA   21 ExpectedInfluence 0.18249122
## 94 graph 1   NA   22 ExpectedInfluence 0.56049268
## 95 graph 1   NA   23 ExpectedInfluence 0.82608238
## 96 graph 1   NA   24 ExpectedInfluence 0.73063013
```

```
cenMCI
```

```
##       graph type node           measure       value
## 1   graph 1   NA    1       Betweenness 0.000000000
## 2   graph 1   NA    2       Betweenness 0.000000000
## 3   graph 1   NA    3       Betweenness 0.070967742
## 4   graph 1   NA    4       Betweenness 0.122580645
## 5   graph 1   NA    5       Betweenness 0.019354839
## 6   graph 1   NA    6       Betweenness 0.025806452
## 7   graph 1   NA    7       Betweenness 0.032258065
## 8   graph 1   NA    8       Betweenness 0.258064516
## 9   graph 1   NA    9       Betweenness 0.432258065
## 10  graph 1   NA   10       Betweenness 0.083870968
## 11  graph 1   NA   11       Betweenness 0.000000000
## 12  graph 1   NA   12       Betweenness 0.141935484
## 13  graph 1   NA   13       Betweenness 0.012903226
## 14  graph 1   NA   14       Betweenness 0.038709677
## 15  graph 1   NA   15       Betweenness 0.000000000
## 16  graph 1   NA   16       Betweenness 0.167741935
```

```
## 17  graph 1   NA   17       Betweenness 0.329032258
## 18  graph 1   NA   18       Betweenness 0.154838710
## 19  graph 1   NA   19       Betweenness 0.025806452
## 20  graph 1   NA   20       Betweenness 0.000000000
## 21  graph 1   NA   21       Betweenness 1.000000000
## 22  graph 1   NA   22       Betweenness 0.032258065
## 23  graph 1   NA   23       Betweenness 0.006451613
## 24  graph 1   NA   24       Betweenness 0.000000000
## 25  graph 1   NA   25       Betweenness 0.012903226
## 26  graph 1   NA   26       Betweenness 0.000000000
## 27  graph 1   NA   27       Betweenness 0.270967742
## 28  graph 1   NA    1         Closeness 0.477363121
## 29  graph 1   NA    2         Closeness 0.626203449
## 30  graph 1   NA    3         Closeness 0.673038181
## 31  graph 1   NA    4         Closeness 0.606582924
## 32  graph 1   NA    5         Closeness 0.538209194
## 33  graph 1   NA    6         Closeness 0.644733644
## 34  graph 1   NA    7         Closeness 0.670230567
## 35  graph 1   NA    8         Closeness 0.862892457
## 36  graph 1   NA    9         Closeness 0.857847389
## 37  graph 1   NA   10         Closeness 0.668788006
## 38  graph 1   NA   11         Closeness 0.388123174
## 39  graph 1   NA   12         Closeness 0.747704130
## 40  graph 1   NA   13         Closeness 0.660530300
## 41  graph 1   NA   14         Closeness 0.585943883
## 42  graph 1   NA   15         Closeness 0.530290683
## 43  graph 1   NA   16         Closeness 0.805467434
## 44  graph 1   NA   17         Closeness 0.858363253
## 45  graph 1   NA   18         Closeness 0.704803462
## 46  graph 1   NA   19         Closeness 0.597030244
## 47  graph 1   NA   20         Closeness          NA
## 48  graph 1   NA   21         Closeness 1.000000000
## 49  graph 1   NA   22         Closeness 0.590419895
## 50  graph 1   NA   23         Closeness 0.542651706
## 51  graph 1   NA   24         Closeness 0.433461573
## 52  graph 1   NA   25         Closeness 0.654526396
## 53  graph 1   NA   26         Closeness 0.591782496
## 54  graph 1   NA   27         Closeness 0.692632788
## 55  graph 1   NA    1          Strength 0.115385292
## 56  graph 1   NA    2          Strength 0.245635142
## 57  graph 1   NA    3          Strength 0.338183247
## 58  graph 1   NA    4          Strength 0.329893857
## 59  graph 1   NA    5          Strength 0.345094165
## 60  graph 1   NA    6          Strength 0.305068230
## 61  graph 1   NA    7          Strength 0.411857045
## 62  graph 1   NA    8          Strength 0.512650840
## 63  graph 1   NA    9          Strength 0.640694745
## 64  graph 1   NA   10          Strength 0.464205786
## 65  graph 1   NA   11          Strength 0.097875957
## 66  graph 1   NA   12          Strength 0.461189269
## 67  graph 1   NA   13          Strength 0.386170888
## 68  graph 1   NA   14          Strength 0.338913617
## 69  graph 1   NA   15          Strength 0.149285023
## 70  graph 1   NA   16          Strength 0.584477328
```

```
## 71  graph 1   NA   17          Strength 0.592718588
## 72  graph 1   NA   18          Strength 0.341308310
## 73  graph 1   NA   19          Strength 0.299338770
## 74  graph 1   NA   20          Strength 0.000000000
## 75  graph 1   NA   21          Strength 1.000000000
## 76  graph 1   NA   22          Strength 0.251427149
## 77  graph 1   NA   23          Strength 0.226127629
## 78  graph 1   NA   24          Strength 0.118401123
## 79  graph 1   NA   25          Strength 0.469988330
## 80  graph 1   NA   26          Strength 0.220087712
## 81  graph 1   NA   27          Strength 0.490333225
## 82  graph 1   NA    1 ExpectedInfluence 0.115385292
## 83  graph 1   NA    2 ExpectedInfluence 0.245635142
## 84  graph 1   NA    3 ExpectedInfluence 0.338183247
## 85  graph 1   NA    4 ExpectedInfluence 0.329893857
## 86  graph 1   NA    5 ExpectedInfluence 0.345094165
## 87  graph 1   NA    6 ExpectedInfluence 0.305068230
## 88  graph 1   NA    7 ExpectedInfluence 0.411857045
## 89  graph 1   NA    8 ExpectedInfluence 0.512650840
## 90  graph 1   NA    9 ExpectedInfluence 0.640694745
## 91  graph 1   NA   10 ExpectedInfluence 0.464205786
## 92  graph 1   NA   11 ExpectedInfluence 0.097875957
## 93  graph 1   NA   12 ExpectedInfluence 0.461189269
## 94  graph 1   NA   13 ExpectedInfluence 0.386170888
## 95  graph 1   NA   14 ExpectedInfluence 0.338913617
## 96  graph 1   NA   15 ExpectedInfluence 0.149285023
## 97  graph 1   NA   16 ExpectedInfluence 0.584477328
## 98  graph 1   NA   17 ExpectedInfluence 0.592718588
## 99  graph 1   NA   18 ExpectedInfluence 0.341308310
## 100 graph 1   NA   19 ExpectedInfluence 0.299338770
## 101 graph 1   NA   20 ExpectedInfluence 0.000000000
## 102 graph 1   NA   21 ExpectedInfluence 1.000000000
## 103 graph 1   NA   22 ExpectedInfluence 0.251427149
## 104 graph 1   NA   23 ExpectedInfluence 0.226127629
## 105 graph 1   NA   24 ExpectedInfluence 0.118401123
## 106 graph 1   NA   25 ExpectedInfluence 0.469988330
## 107 graph 1   NA   26 ExpectedInfluence 0.220087712
## 108 graph 1   NA   27 ExpectedInfluence 0.490333225
```

```
cenAlzheimer
```

```
##       graph type node          measure      value
## 1   graph 1   NA    1       Betweenness 0.56321839
## 2   graph 1   NA    2       Betweenness 0.10344828
## 3   graph 1   NA    3       Betweenness 0.05747126
## 4   graph 1   NA    4       Betweenness 0.06896552
## 5   graph 1   NA    5       Betweenness 0.58620690
## 6   graph 1   NA    6       Betweenness 0.00000000
## 7   graph 1   NA    7       Betweenness 0.35632184
## 8   graph 1   NA    8       Betweenness 0.59770115
## 9   graph 1   NA    9       Betweenness 0.05747126
## 10  graph 1   NA   10       Betweenness 0.89655172
## 11  graph 1   NA   11       Betweenness 0.27586207
## 12  graph 1   NA   12       Betweenness 0.03448276
```

```
## 13  graph 1   NA   13       Betweenness 0.16091954
## 14  graph 1   NA   14       Betweenness 0.00000000
## 15  graph 1   NA   15       Betweenness 0.00000000
## 16  graph 1   NA   16       Betweenness 0.42528736
## 17  graph 1   NA   17       Betweenness 0.72413793
## 18  graph 1   NA   18       Betweenness 0.60919540
## 19  graph 1   NA   19       Betweenness 0.00000000
## 20  graph 1   NA   20       Betweenness 0.00000000
## 21  graph 1   NA   21       Betweenness 0.58620690
## 22  graph 1   NA   22       Betweenness 0.37931034
## 23  graph 1   NA   23       Betweenness 1.00000000
## 24  graph 1   NA   24       Betweenness 0.72413793
## 25  graph 1   NA   25       Betweenness 0.16091954
## 26  graph 1   NA   26       Betweenness 0.16091954
## 27  graph 1   NA    1         Closeness 0.78467154
## 28  graph 1   NA    2         Closeness 0.88905936
## 29  graph 1   NA    3         Closeness 0.56339742
## 30  graph 1   NA    4         Closeness 0.55507115
## 31  graph 1   NA    5         Closeness 0.82825435
## 32  graph 1   NA    6         Closeness 0.41644856
## 33  graph 1   NA    7         Closeness 0.76189616
## 34  graph 1   NA    8         Closeness 0.96689989
## 35  graph 1   NA    9         Closeness 0.72700294
## 36  graph 1   NA   10         Closeness 0.97975743
## 37  graph 1   NA   11         Closeness 0.74392397
## 38  graph 1   NA   12         Closeness 0.59476342
## 39  graph 1   NA   13         Closeness 0.72427030
## 40  graph 1   NA   14         Closeness 0.54078551
## 41  graph 1   NA   15         Closeness 0.61947024
## 42  graph 1   NA   16         Closeness 0.94006766
## 43  graph 1   NA   17         Closeness 0.93227898
## 44  graph 1   NA   18         Closeness 0.97014492
## 45  graph 1   NA   19         Closeness 0.47919494
## 46  graph 1   NA   20         Closeness 0.59442480
## 47  graph 1   NA   21         Closeness 0.74617998
## 48  graph 1   NA   22         Closeness 0.71183142
## 49  graph 1   NA   23         Closeness 1.00000000
## 50  graph 1   NA   24         Closeness 0.93572447
## 51  graph 1   NA   25         Closeness 0.59275856
## 52  graph 1   NA   26         Closeness 0.58706430
## 53  graph 1   NA    1          Strength 0.75102896
## 54  graph 1   NA    2          Strength 0.48540705
## 55  graph 1   NA    3          Strength 0.28633164
## 56  graph 1   NA    4          Strength 0.26177396
## 57  graph 1   NA    5          Strength 0.51264870
## 58  graph 1   NA    6          Strength 0.05134798
## 59  graph 1   NA    7          Strength 0.59425700
## 60  graph 1   NA    8          Strength 0.70595426
## 61  graph 1   NA    9          Strength 0.44792958
## 62  graph 1   NA   10          Strength 0.97901730
## 63  graph 1   NA   11          Strength 0.38561234
## 64  graph 1   NA   12          Strength 0.20952542
## 65  graph 1   NA   13          Strength 0.28514466
## 66  graph 1   NA   14          Strength 0.10663309
```

```
## 67  graph 1   NA   15          Strength 0.21386783
## 68  graph 1   NA   16          Strength 0.78836807
## 69  graph 1   NA   17          Strength 0.84391196
## 70  graph 1   NA   18          Strength 0.51010877
## 71  graph 1   NA   19          Strength 0.07269106
## 72  graph 1   NA   20          Strength 0.11428082
## 73  graph 1   NA   21          Strength 0.54238336
## 74  graph 1   NA   22          Strength 0.30592184
## 75  graph 1   NA   23          Strength 1.00000000
## 76  graph 1   NA   24          Strength 0.64036708
## 77  graph 1   NA   25          Strength 0.48851079
## 78  graph 1   NA   26          Strength 0.54422838
## 79  graph 1   NA    1 ExpectedInfluence 0.75102896
## 80  graph 1   NA    2 ExpectedInfluence 0.48540705
## 81  graph 1   NA    3 ExpectedInfluence 0.28633164
## 82  graph 1   NA    4 ExpectedInfluence 0.26177396
## 83  graph 1   NA    5 ExpectedInfluence 0.51264870
## 84  graph 1   NA    6 ExpectedInfluence 0.05134798
## 85  graph 1   NA    7 ExpectedInfluence 0.59425700
## 86  graph 1   NA    8 ExpectedInfluence 0.70595426
## 87  graph 1   NA    9 ExpectedInfluence 0.44792958
## 88  graph 1   NA   10 ExpectedInfluence 0.97901730
## 89  graph 1   NA   11 ExpectedInfluence 0.38561234
## 90  graph 1   NA   12 ExpectedInfluence 0.20952542
## 91  graph 1   NA   13 ExpectedInfluence 0.28514466
## 92  graph 1   NA   14 ExpectedInfluence 0.10663309
## 93  graph 1   NA   15 ExpectedInfluence 0.21386783
## 94  graph 1   NA   16 ExpectedInfluence 0.78836807
## 95  graph 1   NA   17 ExpectedInfluence 0.84391196
## 96  graph 1   NA   18 ExpectedInfluence 0.51010877
## 97  graph 1   NA   19 ExpectedInfluence 0.07269106
## 98  graph 1   NA   20 ExpectedInfluence 0.11428082
## 99  graph 1   NA   21 ExpectedInfluence 0.54238336
## 100 graph 1   NA   22 ExpectedInfluence 0.30592184
## 101 graph 1   NA   23 ExpectedInfluence 1.00000000
## 102 graph 1   NA   24 ExpectedInfluence 0.64036708
## 103 graph 1   NA   25 ExpectedInfluence 0.48851079
## 104 graph 1   NA   26 ExpectedInfluence 0.54422838
```

Plotting the graphs for the three different group adding the size of each node, representing the Expected "Influence" measure.

```
par(mar = c(1, 1, 1, 1))

plot(communityControlPlot, igraph_Control, layout = Layout_Control,
     #vertex.size=hub.score(igraph_Control)$vector*30,
     vertex.size= cenControls[73:96, 5]*30,
     edge.width=1, main=" Controls group")
```

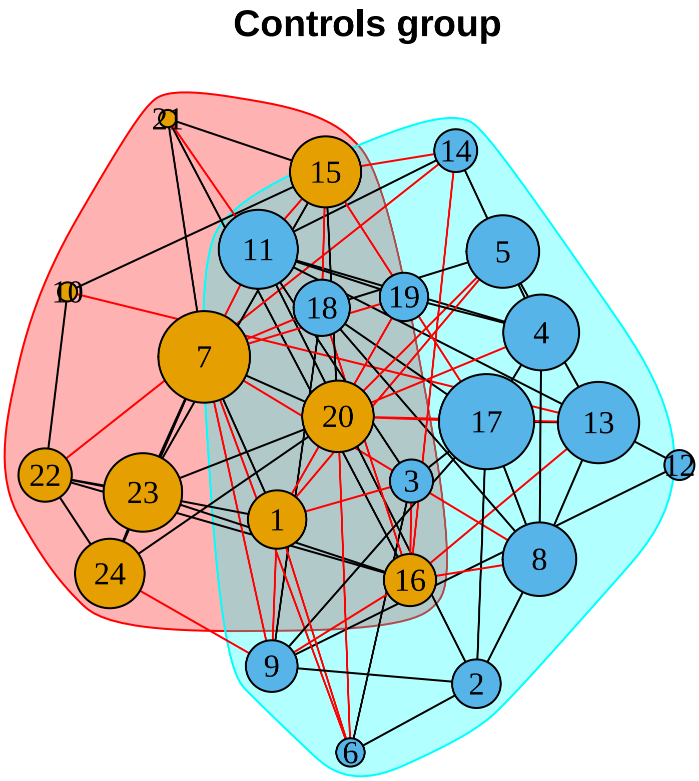


```r
plot(communityMCIPlot, igraph_MCI, layout = Layout_MCI,
     #vertex.size=hub.score(igraph_MCI)$vector*30,
     vertex.size= cenMCI[82:108, 5]*30,
     edge.width=1, main="MCI group")
```

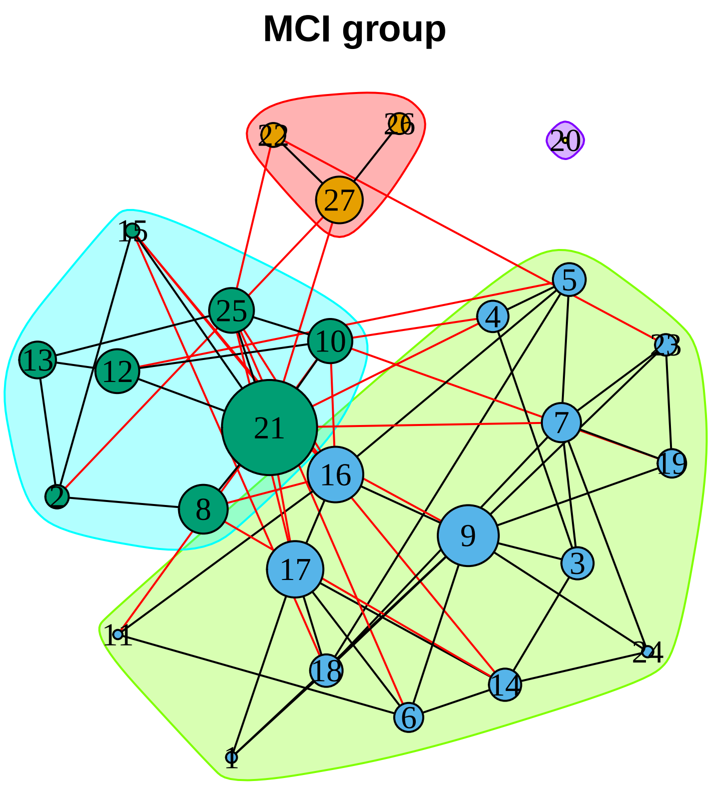


```
plot(communityAlzheimerPlot, igraph_Alzh, layout = Layout_Alzh,
     #vertex.size=hub.score(igraph_Alzh)$vector*30,
     vertex.size= cenAlzheimer[79:104, 5]*30,
     edge.width=1, main="Alzheimer group")
```

Alzheimer group

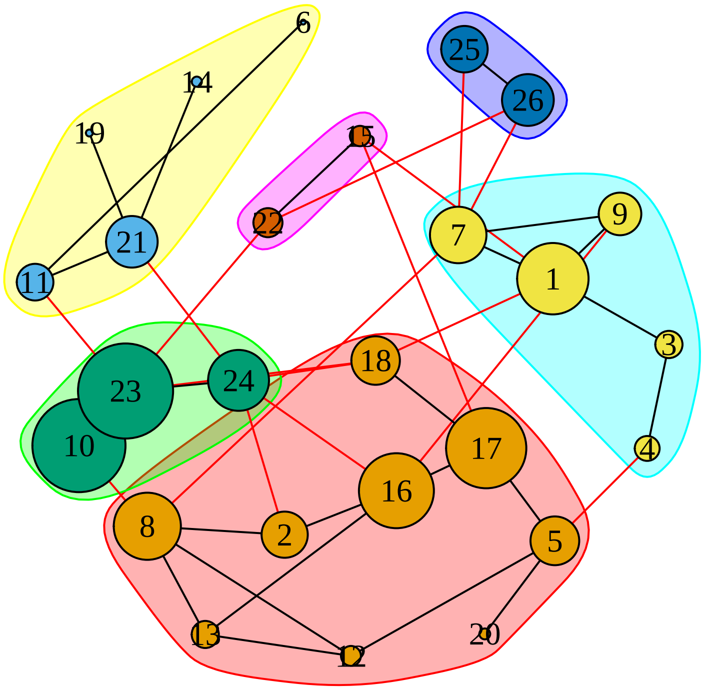

Supplement: Supplementary file 4 — Supplementary Material 4. [file 40359_2024_2239_MOESM4_ESM.pdf]
